# Taxonomic complexity in the genus *Merodon* Meigen, 1803 (Diptera, Syrphidae)

**DOI:** 10.3897/zookeys.1031.62125

**Published:** 2021-04-14

**Authors:** Ante Vujić, Snežana Radenković, Laura Likov, Sanja Veselić

**Affiliations:** 1 University of Novi Sad, Faculty of Sciences, Department of Biology and Ecology, Trg Dositeja Obradovića 2, 21000 Novi Sad, Serbia University of Novi Sad Novi Sad Serbia

**Keywords:** Identification key, integrative taxonomy, lineages, *
Merodon
*, morphology, species groups, Syrphidae

## Abstract

The genus *Merodon* Meigen, 1803 is distributed across the Palaearctic and Afrotropical Regions. The present work summarizes the knowledge from recent taxonomic and systematic revisions and includes an identification key for the five monophyletic lineages (namely *albifrons*, *aureus*, *avidus-nigritarsis*, *desuturinus* and *natans*), 24 species groups, two species subgroups and 10 unplaced species, along with diagnosis and illustrations. A list of 234 taxa, including 194 described and 40 undescribed species, is appended. Most of the species are distributed in the Palaearctic (209 taxa, 181 described), while 27 species (14 described) are known from the Afrotropical Region. Three lineages (*aureus*, *desuturinus* and *natans*) are present in the Afrotropical Region, as well as in the Palaearctic. The Afrotropical *melanocerus* species group of the *desuturinus* lineage and the *bombiformis* species group of the *aureus* lineage are endemic to the Afrotropical Region, and all other species groups belong to the Palaearctic fauna. The *albifrons* lineage contains six species groups (*albifrons*, *constans*, *equestris*, *geniculatus*, *ruficornis* and *rufus*) and two unplaced taxa. The *aureus* lineage includes five species groups (*aureus*, *bombiformis*, *funestus*, *nanus* and *spinitarsis*). The *avidus-nigritarsis* lineage is divided into 10 species groups (*aberrans*, *aurifer*, *avidus*, *clavipes*, *fulcratus*, *italicus*, *nigritarsis*, *pruni*, *serrulatus* and *tarsatus*) and eight unplaced taxa. The *desuturinus* lineage contains two species groups: the Afrotropical *melanocerus* group, with the *melanocerus* and *planifacies* subgroups plus the species *M. cuthbertsoni* Curran, 1939, and the Palaearctic *murorum* species group. The *natans* lineage consists of the *natans* species group plus the species *M. segetum* Fabricius, 1794.

## Introduction

The genus *Merodon* Meigen, 1803 is one of the most species-rich hoverfly genera, distributed across the Palaearctic and Afrotropical Regions (Ståhls et al. 2009; Vujić et al. 2012). It belongs to the tribe Merodontini, formerly named Eumerini. Most recent works used the name Merodontini instead of Eumerini (Skevington and Yeates 2000; Marcos-García et al. 2007; Andrić et al. 2014; Vujić et al. 2015, 2019; Ačanski et al. 2016b; Doczkal et al. 2016; Young et al. 2016; Radenković et al. 2018a; Milić et al. 2019; Šašić Zorić et al. 2019; Likov et al. 2020). However, there is no general consensus on the intratribal structure yet.

The genus *Merodon* was described by Meigen (1803) based on the type species *Syrphus clavipes* Fabricius, 1781. Until now, two synonyms of *Merodon* are recognized: *Lampetia* Meigen, 1800, originally described without included species, was suppressed by ICZN (1963, Opinion 678: 339); and *Exmerodon*, created by Becker (1913) as a subgenus of *Merodon* based on the type species *Exmerodon fulcratus* Becker, 1913, was listed as synonym by Peck (1988). Currently, the genus *Merodon* contains 194 described species and 40 undescribed species listed here. The genus is restricted to the Palaearctic and Afrotropical Regions (Ståhls et al. 2009; Šašić et al. 2016), except for *M. equestris* (Fabricius, 1794) that has been introduced into the Nearctic Region and New Zealand (Speight 2020). The genus is divided in lineages, species groups, species subgroups and species complexes following the system proposed by Šašić et al. (2016) based on different levels of morphological differentiation. Šašić et al. (2016) proposed a system of four levels (ranks) for classification of the genus *Merodon*: (1) the broadest (first) level consists of large monophyletic lineages where each contains multiple morphologically different species groups; (2) the second broadest level involves taxa that constitute morphologically defined species groups within lineages; (3) the third level represents subgroups that include species with very similar morphologies, but exhibiting small, consistent interspecific character variations that facilitate their distinction; (4) the narrowest (fourth) level are species complexes that comprise morphologically inseparable taxa based on classical taxonomical methods, which can only be resolved by employing integrative taxonomy involving molecular markers, morphometry, and ecology.

In Europe, *Merodon* is the most speciose genus with 120 described species (152 including Turkey) (Speight 2020; Vujić, unpublished). The highest species diversity is recorded for the Mediterranean Basin (Vujić et al. 2012), which is associated with a high diversity of bulb plant species that serve as larval host plants (Ricarte et al. 2008, 2017; Andrić et al. 2014; Preradović et al. 2018). Asia Minor and Eastern Europe (especially the Balkan Peninsula) are considered hot spots and regions with high endemism for the genus *Merodon* (Kaloveloni et al. 2015), as documented by several studies in the Eastern Mediterranean Basin (Vujić et al. 2007, 2011, 2013, 2015, 2020a, b, c; Ståhls et al. 2009, 2016; Radenković et al. 2011, 2020; Kaloveloni et al. 2015; Ačanski et al. 2016a, b; Likov et al. 2020). Unlike this area, Afrotropical Region and Eastern Palaearctic are characterized by a low number of species (Vujić, unpublished).

The genus *Merodon* was classified into more than 20 monophyletic species groups, half of which were addressed in Hurkmans’ (1993) monograph. Hurkmans (1993) gave the first and most comprehensive revision of the genus, placing 61 species with tapering abdomen into 11 species groups, namely *alagoezicus*, *alexeji*, *avidus*, *clavipes*, *crassifemoris*, *elegans*, *longicornis*, *nigritarsis*, *pruni*, *tarsatus* and *vandergooti*. Mengual et al. (2006) discerned four species groups (*desuturinus*, *albifrons*, *nigritarsis* and *aureus*) based on molecular data among the species occurring in the Iberian Peninsula. Vujić et al. (2019) recognized five monophyletic lineages within the genus *Merodon*, i.e., *albifrons*, *aureus*, *avidus-nigritarsis*, *desuturinus* and *natans*, condensing previous studies from Šašić et al. (2016) and Radenković et al. (2018a). The *albifrons+desuturinus* lineage *sensu*Vujić et al. (2012) is now divided into two lineages, *albifrons* and *desuturinus*.

Nowadays, with the advent of molecular and morphometric techniques, an integrative taxonomic framework has become the standard to study the taxonomy of genus *Merodon*. Combining molecular characters (mtDNA cytochrome *c* oxidase subunit I (COI) and the nuclear 28S rRNA genes) with morphological traits (geometric wing morphometry, surstylus shape and size, and other morphological characters), a number of cryptic and sibling species have been delineated within different species groups. Notable examples are the *ruficornis* species group (Radenković et al. 2002; Milankov et al. 2008c; Francuski et al. 2009; Vujić et al. 2012), *desuturinus* species group (Milankov et al. 2008b; Vujić et al. 2018b), *aureus* and *cinereus* species subgroups (Milankov et al. 2008a; Francuski et al. 2011; Šašić et al. 2016; Veselić et al. 2017; Radenković et al. 2018b), *avidus* species complex (Milankov et al. 2009; Popović et al. 2015; Ačanski et al. 2016b), *albifrons* species group (Milankov et al. 2013), *nigritarsis* species group (Vujić et al. 2013; Likov et al. 2020), *nanus* species group (Vujić et al. 2015; Kočiš Tubić et al. 2018), *serrulatus* species group (Vujić et al. 2020b), *constans* species group (Vujić et al. 2020a), *rufus* species group (Radenković et al. 2020), and all *Merodon* species of Lesvos Island (Ståhls et al. 2009).

The aim of this work is to summarize the knowledge from recent taxonomic and systematic revisions, to help taxonomists to have a central reference for the recent published literature, and to present an identification key for the identification of lineages, species groups, species subgroups and unplaced species of *Merodon*.

## Material and methods

A total of 255 species belonging to the tribe Merodontini (genera *Azpeytia* Walker, 1865, *Eumerus* Meigen, 1822, *Megatrigon* Johnson, 1898, *Merodon* and *Platynochaetus* Weidemann, 1830) from the Palaearctic and Afrotropical Regions were studied. All specimens were identified by Ante Vujić and Snežana Radenković. Representative specimens are deposited in the collections of the Department of Biology and Ecology, Faculty of Sciences, University of Novi Sad, Serbia (**FSUNS**).

Morphological terminology follows Thompson (1999), except for the male genitalia that follows Marcos-García et al. (2007). We use the terms “fossette”, “hypostomal bridge”, “postalar” and “occipital foramen” from Doczkal and Pape (2009), and “oral margin” from Radenković et al. (2018a). For the pollinose markings on abdominal terga we used the term fasciate maculae. These markings are elongate and usually separated medially. Sometimes the fasciate maculae may have joined medially forming an entire fascia or band, but we consistently referred to them as fasciate maculae.

Male genitalia were extracted from dry specimens previously relaxed in a humidity chamber. After genitalia were pulled out with a hook-tipped entomological pin, they were cleared by boiling in warm 10% potassium hydroxide (KOH) for 3–5 min. Acetic acid was then used to neutralize the KOH during 5 s, and genitalia were immersed briefly in ethanol to remove the acid. Prepared genitalia were stored in microvials containing glycerol.

Photographs were taken using a Leica DFC 320 digital camera, attached to a Leica MZ16 stereomicroscope and Nikon Coolpix D7100 digital camera attached to a Nikon SMZ 745T stereomicroscope. Digital photographs were stacked using CombineZ software (Hadley 2006). A Leica MZ16 binocular microscope was used with an FSA 25 PE drawing tube to make the drawings.

## Results

### Tribe Merodontini

Based on Doczkal and Pape (2009), members of the Merodontini possess six autapomorphic character states: hypostomal bridge close to the occipital foramen with a transverse crest (Suppl. material 1: Fig. S1A: marked with arrow), absent in others (Suppl. material 1: Fig. S1B); presence of a pyramidal tubercle on the postalar wall (Suppl. material 1: Fig. S2A: marked with arrow), flat in others (Suppl. material 1: Fig. S2B: marked with arrow); dorsomedian part of anepimeron setose (Suppl. material 1: Fig. S2C: marked with arrow), bare in others (Suppl. material 1: Fig. S2D: marked with arrow); presence of a well-defined fossette (Suppl. material 1: Fig. S3C: marked with arrow), absent in others (Suppl. material 1: Fig. S3D); wing vein R_1_ joining C beyond 0.6 of the distance from Sc to R_2+3_ (Suppl. material 1: Fig. S3A), different in others (Suppl. material 1: Fig. S3B); and distal end of M_1_ recurrent, forming an acute outer angle with R_4+5_ (Suppl. material 1: Fig. S3A: marked with arrow), obtuse in others (Suppl. material 1: Fig. S3B: marked with arrow). The first two character states are unique to the Merodontini, whereas the remaining character states are homoplasious with occurrences elsewhere in Syrphidae (Doczkal and Pape 2009). Other autapomorphies of the Merodontini, not present in *Lyneborgimyia* Doczkal & Pape, 2009, are the presence of a lateral sclerite of the aedeagus (Suppl. material 2: Fig. S10F: s) and ventral processes and/or invaginations of the hypandrium (Suppl. material 2: Fig. S10F: marked with arrow) (Doczkal and Pape 2009).

#### Genus *Merodon*

*Merodon* Meigen, 1803, Mag. Insektenk, 2, 274. Type-species: *Syrphus clavipes* Fabricius, 1781, by subsequent designation of Guérin-Méneville in Bory de Saint-Vincent 1826: 446.

*Lampetia* Meigen, 1800, Nouvelle classification des mouches à deux ailes (Diptera L.) d’après un plan tout nouveau J.J. Fuchs, Paris: 34. Type-species: *Syrphus clavipes* Fabricius, 1781, by subsequent designation of Coquillett, 1910: 557. Suppressed by ICZN 1963: Opinion 678: 339.

**Differential diagnosis.** The genus can be distinguished by the presence of an antero-ventral triangular lamina above the apex of the metafemur (as in Suppl. material 1: Fig. S15D or Suppl. material 1: Fig. S28E, F), wing vein R_4+5_ with a deep loop into cell r_4+5_, and veins Sc and R_1_ connected with a stigmal crossvein (Suppl. material 1: Fig. S3A: marked with asterisk).

As mentioned earlier, there are five monophyletic lineages within the genus *Merodon*: *albifrons*, *aureus*, *avidus-nigritarsis*, *desuturinus*, and *natans* (Vujić et al. 2019). The main morphological features and the list and number of species are presented in Supplementary materials.

### Identification key to the *Merodon* lineages

In this section and sections below, we provide several identification keys to the 24 species groups, two species subgroups and 10 unplaced species within the genus *Merodon*. For further species identification inside species groups, species subgroups and species complexes, published revisionary works are cited in brackets.

**Table:** 

1	Mesocoxa without long pile posteriorly (Suppl. material 1: Fig. S4B), or if mesocoxa with 1–3 long pile posteriorly then inner side of metafemur with a row of spinae (Suppl. material 1: Fig. S5A)	***avidus-nigritarsis* lineage**
–	Mesocoxa with at least a few long pile posteriorly (Suppl. material 1: Fig. S4A), inner side of metafemur without a row of spinae	**2**
2	Mesocoxa with more than 10 long pile posteriorly	**4**
–	Mesocoxa with a few long pile posteriorly (usually five to seven, or less) (Suppl. material 1: Fig. S5B)	**3**
3	Basoflagellomere elongated, twice as long as wide, narrowed in apical third (Suppl. material 1: Fig. S6A); scutum usually with five well defined pollinose longitudinal vittae (Suppl. material 1: Fig. S6C). Anterior surstyle lobe of male genitalia well-developed, oval, without curved distal prolongation (Suppl. material 2: Fig. S13A: al)	***natans* lineage**
–	Basoflagellomere less than half as long as wide, narrowed in apical half (Suppl. material 1: Fig. S6B); scutum without pollen or with less distinct pollinose longitudinal vittae (Suppl. material 1: Fig. S6D). Anterior surstyle lobe of male genitalia with curved distal prolongation (Suppl. material 2: Fig. S12E, H: al)	***desuturinus* lineage (in part)**
4	Anterior anepisternum with bare area ventral to postpronotum (Suppl. material 1: Fig. S7B)	**6**
–	Anterior anepisternum with many long pile ventral to postpronotum (Suppl. material 1: Fig. S7A)	**5**
5	Postpronotum usually brown or yellow-reddish. Male genitalia: anterior surstyle lobe with curved distal prolongation (Suppl. material 2: Fig. S12E, H: al). Female: pilosity on lateral side of tergum 4 long, medially short and mostly adpressed (Suppl. material 1: Fig. S8A)	***desuturinus* lineage (in part)**
–	Postpronotum black. Male genitalia: anterior surstyle lobe undeveloped (Suppl. material 2: Fig. S4A: al). Female: all the pilosity on tergum 4 approximately of same length (Suppl. material 1: Fig. S8B)	***aureus* lineage**
6	Lateral sclerite of the aedeagus gradually tapered, with the tip curved (Suppl. material 2: Fig. S12C: s); wing microtrichose between veins R_1_ and RS (Suppl. material 1: Fig. S9A)	***desuturinus* lineage (in part)**
–	Lateral sclerite of the aedeagus hammer-like (Suppl. material 2: Fig. S2C: s); wing with bare area in the basal part of wing cell r_1_, between veins R_1_ and RS (Suppl. material 1: Fig. S9B)	***albifrons* lineage**

### Key to the species groups and unplaced species of the *albifrons* lineage

**Table:** 

1	Postpronotum, lateral sides of scutum and face yellowish (Suppl. material 1: Fig. S31)	***Merodon luteihumerus* Marcos-García, Vujić & Mengual, 2007**
–	Postpronotum, lateral sides of scutum and face black or dark	**2**
2	Pro- and mesolegs strongly modified (Suppl. material 1: Fig. S32A, C)	***Merodon mixtum* Vujić, Radenković & Likov, 2019**
–	Pro- and mesolegs with usual shape	**3**
3	Pilosity on the posterior part of abdomen (at least tergum 4) denser and strikingly golden to reddish-yellow (as in Suppl. material 1: Fig. S33A) contrasting with the colour of the pilosity on the rest of the abdomen	***constans* species group (Vujić et al. 2020a)**
–	Pilosity on the posterior part of abdomen not denser compared to the anterior part (Suppl. material 3: Fig. S2B)	**4**
4	Large (11–17 mm) bumble bee-like species with dense and long body pilosity (Suppl. material 3: Fig. S2A, B); males with strong apical dorsal calcar on metatibia (Suppl. material 1: Fig. S34A)	***equestris* species group (Marcos-García et al. 2011)**
–	Species with different characters	**5**
5	Medium to large sized species (9–13 mm) with black, bronze lustre terga (lack pollinose fasciate maculae), except a pair of small, orange, lateral markings on tergum 2 (Suppl. material 1: Fig. S33B); scutum and terga covered with erect, mostly yellowish to reddish pilosity, except few black pile medially on terga 3 and 4 (Suppl. material 1: Fig. 33C). Male: metatrochanter can have a calcar, but metatibia always without calcar (Suppl. material 1: Fig. S34B)	***rufus* species group (Radenković et al. 2020)**
–	Species with different characters; usually with pollinose fasciate maculae on terga	**6**
6	Female (genitalia not visible)	**9**
–	Male (genitalia visible externally)	**7**
7	Metaleg with some modifications on metatrochanter, metafemur and/or metatibia; male genitalia often with hook-like posterior surstyle lobe or cercus with prominence(s)	**8**
–	Metaleg usually without modifications (exception is *Merodon trochantericus* Costa, 1884, see in Suppl. material 1: Fig. S34C); male genitalia with rounded posterior surstyle lobe, biramous anterior surstyle lobe pliers-like (with thorn-like interior accessory lobe process), and cercus without prominences (as in Suppl. material 2: Fig. S2A)	***albifrons* species group**
8	Metatrochanter with blunt calcar apically covered with long pile (Suppl. material 1: Fig. S34D); metatibia with apicomedial carina (Suppl. material 1: Fig. S34E); male genitalia with rounded or acute posterior surstyle lobe, biramous anterior surstyle lobe with apical extension more developed, and cercus with prominence(s) (as in Suppl. material 2: Fig. S2G: marked with arrow)	***geniculatus* species group (Vujić et al. 2018a)**
–	Metatrochanter with sharp apical calcar (Suppl. material 1: Fig. S34F); metafemur usually with ventral tubercle or calcar (as on Suppl. material 1: Fig. S34F: marked with arrow); metatibia usually with apicolateral process (as on Suppl. material 1: Fig. S34F: marked with arrow); male genitalia usually with hook-like posterior surstyle lobe, biramous anterior surstyle lobe with moderately developed interior accessory lobe and apical extension, and cercus without prominences (as in Suppl. material 2: Fig. S3A: c)	***ruficornis* species group (Vujić et al. 2012)**
9	Metatibia narrow, not swollen apically (Suppl. material 1: Fig. S34G); terga 3–5 black	***ruficornis* species group (Vujić et al. 2012)**
–	Metatibia incrassate apically (Suppl. material 1: Fig. S34H); terga 3–5 usually partly reddish	**10**
10	Scutum usually with five distinct pollinose vittae (Suppl. material 1: Fig. S35A); terga 2–4 with well-defined pollinose fasciate maculae (Suppl. material 1: Fig. S35B); metatibia with concave ventral margin in apical half (Suppl. material 1: Fig. S34H)	***geniculatus* species group (Vujić et al. 2018a)**
–	Scutum with indistinct pollinose vittae; terga 2–4 without or with narrower pollinose fasciate maculae (Suppl. material 1: Fig. S35C); metatibia with straight ventral margin in apical half (Suppl. material 1: Fig. S33D)	***albifrons* species group**

### Key to the groups and unplaced species and species of the *aureus* lineage

**Table:** 

1	Female (genitalia not visible)	**6**
–	Male (genitalia visible externally)	**2**
2	Metatrochanter with calcar (Suppl. material 1: Fig. S28B marked with arrow)	**5**
–	Metatrochanter rounded and smooth, without calcar (Suppl. material 1: Fig. S28A)	**3**
3	Hypandrium strongly modified, sinuous in apical half, with subapical ctenidium and stitched theca (cuticle looks like it is sewed) (Suppl. material 2: Fig. S4K)	***spinitarsis* species group**
–	Hypandrium different, but with apical ctenidium and without stitch on theca (as in Suppl. material 2: Fig. S5C)	**4**
4	Pedicel elongated, approximately as long as basoflagellomere (relation pedicel basoflagellomere = 0.9 : 1.1) (Suppl. material 1: Fig. S29A); hypandrium narrowed medially (Suppl. material 2: Fig. S5C: marked with arrow)	***bombiformis* species group (Afrotropical Region)**
–	Pedicel shorter than basoflagellomere (as in Suppl. material 1: Fig. S29B); hypandrium broad, not narrowed medially (Suppl. material 2: Fig. S4G)	***nanus* species group (Vujić et al. 2015; Kočiš Tubić et al. 2018)**
5	Yellow-grey pilosity on terga more dense and striking laterally, as well as on pollinose fasciate maculae of terga 2 and 3, and on tergum 4 (Suppl. material 1: Fig. S30A); pedicel elongated, approximately as long as basoflagellomere (relation pedicel : basoflagellomere = 0.9 : 1.1) (Suppl. material 1: Fig. S29C)	***funestus* species group**
–	Terga evenly covered with upstanding, dense pilosity (can be differently coloured) (Suppl. material 1: Fig. S30B); pedicel shorter than basoflagellomere (Suppl. material 1: Fig. S29D)	***aureus* species group (Šašić et al. 2016, 2018, 2019; Veselić et al. 2017; Radenković et al. 2018b; Vujić et al. 2020c)**
6	Pedicel shorter than basoflagellomere (Suppl. material 1: Fig. S29F)	**8**
–	Pedicel elongated, approximately as long as basoflagellomere (relation pedicel : basoflagellomere = 0.9 : 1.1) (Suppl. material 1: Fig. S29E)	**7**
7	Apical triangular lamina on metafemur weakly serrated, usually with distinct apical dens (Suppl. material 1: Fig. S28E: marked with arrow)	***bombiformis* species group (Afrotropical Region)**
–	Apical triangular lamina on metafemur markedly serrated (Suppl. material 1: Fig. S28F: marked with arrow)	***funestus* species group**
8	Terga without pollinose fasciate maculae, with dense puncta (Suppl. material 1: Fig. S30C)	**9**
–	Terga 2–4 (at least 2) usually with pollinose medial fasciate maculae, with less dense puncta (Suppl. material 1: Fig. S30D)	***aureus* species group (Šašić et al. 2016, 2018, 2019; Veselić et al. 2017; Radenković et al. 2018b; Vujić et al. 2020c)**
9	Tergum 4 covered with short adpressed pile (Suppl. material 1: Fig. S30E); tarsi black (Suppl. material 1: Fig. S28C)	***spinitarsis* species group**
–	Tergum 4 covered with longer semi-erect pile, longer than basoflegellomere (Suppl. material 1: Fig. S30F); tarsi partly reddish (Suppl. material 1: Fig. S28D)	***nanus* species group (Vujić et al. 2015; Kočiš Tubić et al. 2018)**

### Key to the species groups and unplaced species of the *avidus-nigritarsis* lineage

**Table:** 

1	Inner side of metafemur with a row of spinae (Suppl. material 1: Fig. S5A). Male with two fossette (small apical one on the inner side, and a large one on the outer side) (Suppl. material 1: Fig. S10A, B); mesocoxa with 1–3 long pile posteriorly	***Merodon eumerusi* Vujić, Radenković & Likov, 2019**
–	Inner side of metafemur without a row of spinae	**2**
2	Terga partly brown, reddish or yellow	**22**
–	Terga black	**3**
3	Females (genitalia not visible)	**13**
–	Males (genitalia visible externally)	**4**
4	Male genitalia without ctenidium at hypandrium (Suppl. material 2: Fig. S7K: marked with arrow); small sized species (5–9 mm) with metallic shiny body and distinctly dichoptic eyes, separated by distance almost as long as distance between ocelli (Suppl. material 1: Fig. S11A); metafemur with very small apical triangular lamina apicoventrally (Suppl. material 1: Fig. S12A: marked with arrow)	***fulcratus* species group**
–	Male genitalia always with ctenidium at hypandrium (as in Suppl. material 2: Fig. S7C: marked with arrow)	**5**
5	Large species (15–20 mm) with long body pilosity and broad metafemur covered with long pile (Suppl. material 1: Fig. S12B); basoflagellomere elongated; terga usually covered with pile in different combinations of colours (white, yellow or black) (Suppl. material 3: Fig. S9A, B); surstyle with well-defined and large anterior and posterior lobes (Suppl. material 2: Fig. S7A: al, pl)	***clavipes* species group**
–	Species with shorter pilosity and different combinations of characters	**6**
6	Sternum 4 medially clearly divided with membranous structure and with posterolateral tubercles or laminate extensions (Suppl. material 1: Fig. S11C: marked with arrow); sternum 4 from lateral view usually fin-form (Suppl. material 1: Fig. S11D: marked with arrow); basotarsomere of metatarsus usually expanded (Suppl. material 1: Fig. S12C, D) and/or with strong setae ventrally (Suppl. material 1: Fig. S12C, D: marked with arrow)	***tarsatus* species group**
–	Sternum 4 and basotarsomere of metatarsus without such modifications	**7**
7	Male genitalia: posterior surstyle lobe divided into two branches (Suppl. material 2: Fig. S9J: pl); eyes slightly dichoptic, distance between eyes about two facets long (Suppl. material 1: Fig. S11B)	***Merodon hirtus* Sack, 1932**
–	Male genitalia: posterior surstyle lobe not divided into branches; eyes holoptic	**8**
8	Abdomen elongated and narrow; terga black; terga 2–4 with a pair of pollinose fasciate maculae (Suppl. material 1: Fig. S11E); metafemur usually long and narrow. Male genitalia: hypandrium with very long lingula (Suppl. material 2: Fig. S6C: l); posterior surstyle lobe with inner lobe covered with long and strong setae (Suppl. material 2: Fig. S6B)	***aberrans* species group**
–	Species with different combinations of characters	**9**
9	Basoflagellomere elongated, at least three times as long as wide (Suppl. material 1: Fig. S13A); posterior surstyle lobe quadratic (Suppl. material 2: Fig. S7D: pl)	***italicus* species group (in part)**
–	Basoflagellomere less elongated; posterior surstyle lobe different	**10**
10	Posterior surstyle lobe with basolateral protrusion (lateral hump) (Suppl. material 2: Fig. S9G: marked with arrow). Metafemur with shorter pilosity ventrally, shorter than width of metafemur (Suppl. material 1: Fig. S14A); basoflagellomere narrow and elongated, two times longer as pedicel (Suppl. material 1: Fig. S13B)	***serrulatus* species group (in part) (Vujić et al. 2020b)**
–	Posterior surstyle lobe of male genitalia without basolateral protrusion (lateral hump)	**11**
11	Basoflagellomere reddish-yellow (Suppl. material 1: Fig. S13C); tarsus of metaleg yellow (at least basotarsomere) (Suppl. material 1: Fig. S14B); metatarsus long, more than three times longer than wide (Suppl. material 1: Fig. S14B); metafemur less incrassate, ca. four times longer than wide (Suppl. material 1: Fig. S14B)	***Merodon ottomanus* Hurkmans, 1993**
–	Basoflagellomere brown to black; tarsi of metaleg dark; metatarsus shorter, two times longer than wide (Suppl. material 1: Fig. S14D); metafemur more incrassate, ca. three times longer than wide (Suppl. material 1: Fig. S14C)	**12**
12	Basoflagellomere with convex dorsal margin (Suppl. material 1: Fig. S13D); posterior surstyle lobe with the apical hump directed towards cercus (Suppl. material 2: Fig. S10G: marked with arrow)	***Merodon clunipes* Sack, 1913 (in part)**
–	Basoflagellomere with concave dorsal margin (Suppl. material 1: Fig. S13E); posterior surstyle lobe without the apical hump directed towards cercus (Suppl. material 2: Fig. S10A: pl)	***Merodon auronitens* Hurkmans, 1993**
13	Metafemur more incrassate, ca. three times longer than wide, covered with dense pilosity (Suppl. material 1: Fig. S15A)	**14**
–	Metafemur less incrassate, at least three times longer than wide (as in Suppl. material 1: Fig. S15B)	**15**
14	Basoflagellomere shorter, 1.3 times as long as wide, with convex dorsal margin (Suppl. material 1: Fig. S16A); pile on ventral margin of metafemur shorter, ca. one third of width of metafemur (Suppl. material 1: Fig. S15A)	***Merodon clunipes* Sack, 1913 (in part)**
–	Basoflagellomere elongated, two times as long as wide (Suppl. material 1: Fig. S16B); pile on ventral margin of metafemur longer, ca. half of width of metafemur (Suppl. material 1: Fig. S15C)	***clavipes* species group (in part)**
15	Small sized species (8-11 mm) with metallic shiny body; scutum and terga strongly punctate, without or with very weak pollinose areas (Suppl. material 1: Fig. S17A, B); metafemur with very small apical triangular lamina apicoventrally (Suppl. material 1: Fig. S15D marked with arrow)	***fulcratus* group**
–	Species with different combinations of characters	**16**
16	Metafemur with short pilosity (Suppl. material 1: Fig. S15E)	***serrulatus* species group (in part) (Vujić et al. 2020b)**
–	Metafemur with longer pile (as in Suppl. material 1: Fig. S15B)	**17**
17	Abdomen broad, oval (Suppl. material 1: Fig. S17C); terga without pollinosity or with very weak pollinose fasciate maculae; tarsus of metaleg yellow (at least basotarsomere) (Suppl. material 1: Fig. S15B)	***Merodon ottomanus* Hurkmans, 1993**
–	Species with different combinations of characters	**18**
18	Basotarsomere of metatarsus elongated, four times as long as wide (Suppl. material 1: Fig. S15F); basoflagellomere elongated, 2.5 times as long as wide (Suppl. material 1: Fig. S16C); tarsi yellow, tibiae mostly yellowish, except medially brown (Suppl. material 1: Fig. S15F)	***Merodon murinus* Sack, 1913 (in part)**
–	Species with different combinations of characters	**19**
19	Abdomen narrow, elongated (Suppl. material 1: Fig. S17D); metaleg usually narrow (as in Suppl. material 1: Fig. S18A)	***aberrans* species group**
–	Species with broader abdomen and metaleg	**20**
20	Tergum 2 without or with indistinct narrow pollinose fasciate maculae (Suppl. material 1: Fig. S19A, B); basotarsomere of metatarsus usually expanded (Suppl. material 1: Fig. S18B) or with strong setae ventrally (Suppl. material 1: Fig. S18B marked with arrow)	***tarsatus* species group**
–	Tergum 2 with broad pollinose fasciate maculae (Suppl. material 1: Fig. S19C); basotarsomere of metatarsus not expanded and without strong setae ventrally (Suppl. material 1: Fig. S18C)	**21**
21	Terga 2–4 strongly punctate; second and third tarsomeres similar in size (Suppl. material 1: Fig. S18D marked with arrow); sterna shiny	***Merodon auronitens* Hurkmans, 1993**
–	Terga 2–4 finely punctate; second tarsomere longer than third (Suppl. material 1: Fig. S18C marked with arrow); sterna dull	***Merodon hirtus* Sack, 1932**
22	Females (genitalia not visible)	**31**
–	Males (genitalia visible externally)	**23**
23	Metatibia swollen in apical half (Suppl. material 1: Fig. S20A); basotarsomere of metatarsus strongly modified (Suppl. material 1: Fig. S20A)	***Merodon caudatus* Sack, 1913**
–	Metaleg without such modifications	**24**
24	Posterior surstyle lobe with basolateral protrusion (lateral hump) (Suppl. material 2: Fig. S9G: marked with arrow)	***serrulatus* species group (in part) (Vujić et al. 2020b)**
–	Posterior surstyle lobe without basolateral protrusion	**25**
25	Face with a bulge below antennae (Suppl. material 1: Fig. S21A: marked with arrow); posterior surstyle lobe hook-like (Suppl. material 2: Fig. S10J: pl)	***Merodon crassifemoris* Paramonov, 1925**
–	Face without a bulge below antennae	**26**
26	Metatrochanter without calcar	**28**
–	Metatrochanter with distinct calcar (Suppl. material 1: Fig. S20B: marked with arrow)	**27**
27	Basoflagellomere 1.2 times as long as wide (Suppl. material 1: Fig. S23A); body pilosity very short; terga 3–4 dark (Suppl. material 1: Fig. S22A)	***aurifer* species group**
–	Basoflagellomere short, as long as wide (Suppl. material 1: Fig. S23B); body pilosity longer; terga 3–4 mostly yellow-red (Suppl. material 1: Fig. S22B)	***pruni* species group**
28	Basoflagellomere elongated, at least three times as long as wide (Suppl. material 1: Fig. S13A); posterior surstyle lobe quadratic (Suppl. material 2: Fig. S7D: pl)	***italicus* species group** (in part)
–	Basoflagellomere shorter, less than three times as long as wide (as in Suppl. material 1: Fig. S23C); posterior surstyle lobe different	**29**
29	Eye contiguity very short, approximately four to five facets long (Suppl. material 1: Fig. S21B); male genitalia in Suppl. material 2: Fig. S11D–F	***Merodon murinus* Sack, 1913 (in part)**
–	Eye contiguity more than 10 facets long (as in Suppl. material 1: Fig. S21C); male genitalia different	**30**
30	Tarsi yellow dorsally and ventrally (Suppl. material 1: Fig. S20C, D)	***avidus* species group (Popović et al. 2015; Ačanski et al. 2016b; Likov et al. 2020)**
–	Tarsi dark brown/black dorsally and orange/brown ventrally (Suppl. material 1: Fig. S20E, F)	***nigritarsis* species group (Vujić et al. 2013; Likov et al. 2020)**
31	At least terga 2 and 3 with brown, reddish or yellow markings	**36**
–	Only tergum 2 with brown, reddish or yellow maculae, other terga dark	**32**
32	Metatibia swollen in apical half (Suppl. material 1: Fig. S24A); tarsomeres of mesotarsus with strong, black lateral setae (Suppl. material 1: Fig. S24B)	***Merodon caudatus* Sack, 1913**
–	Metatibia of normal shape (as in Suppl. material 1: Fig. S24C); tarsomeres of mesotarsus without such lateral setae	**33**
33	Pile on ventral margin of metafemur dense and long, the longest as long as width of metafemur (Suppl. material 1: Fig. S15C)	***clavipes* species group**
–	Pile on ventral margin of metafemur shorter, maximum as long as half of width of metafemur (as in Suppl. material 1: Fig. S15A)	**34**
34	Basoflagellomere shorter, 1.3 times as long as wide, with convex dorsal margin (Suppl. material 1: Fig. S16A); metafemur incrassate or swollen, ca. three times longer than wide (Suppl. material 1: Fig. S15A)	***Merodon clunipes* Sack, 1913 (in part)**
–	Basoflagellomere longer, with straight or concave dorsal margin (Suppl. material 1: Fig. S16C); metafemur less incrassate	**35**
35	Tarsi yellow, tibiae mostly yellowish, only medially brown; frons and vertex usually partly reddish to yellow (Suppl. material 1: Fig. S25A)	***Merodon murinus* Sack, 1913 (in part)**
–	Legs mostly black, at least tarsi dark; frons black	***serrulatus* species group (in part) (Vujić et al. 2020b)**
36	Basoflagellomere elongated, more than 1.5 times as long as wide (Suppl. material 1: Fig. S26B); metatrochanter with rounded ventral margin (as in Suppl. material 1: Fig. S24D)	**38**
–	Basoflagellomere shorter, less than 1.3 times as long as wide (Suppl. material 1: Fig. S26A); metatrochanter with angular ventral margin (Suppl. material 1: Fig. S24C: marked with arrow)	**37**
37	Basoflagellomere very short, as long as wide (Suppl. material 1: Fig. S26A); metafemur dorsally and ventrally covered with longer outstanding pile (Suppl. material 1: Fig. S24C)	***pruni* species group**
–	Basoflagellomere longer, 1.2 times as long as wide (Suppl. material 1: Fig. S26C); metafemur covered with short and adpressed pilosity (Suppl. material 1: Fig. S24E)	***aurifer* species group**
38	Face with a bulge below antennae (Suppl. material 1: Fig. S25B: marked with arrow)	***Merodon crassifemoris* Paramonov, 1925**
–	Face without a bulge below antennae	**39**
39	Basoflagellomere elongated, at least 2.7 times as long as wide (Suppl. material 1: Fig. S26D); terga 2 and 3 reddish (Suppl. material 1: Fig. S25C)	***italicus* species group (in part)**
–	Basoflagellomere shorter, less than 2.5 times as long as wide (as in Suppl. material 1: Fig. S26E)	**40**
40	Tarsi yellow dorsally and ventrally (as in Suppl. material 1: Fig. S20C, D)	***avidus* species group (Popović et al. 2015; Ačanski et al. 2016b; Likov et al. 2020)**
–	Tarsi dark brown/black dorsally and orange/brown ventrally (as in Suppl. material 1: Fig. S20E, F)	***nigritarsis* species group (Vujić et al. 2013; Likov et al. 2020)**

### Key to the species group, species subgroups and unplaced species of the *desuturinus* lineage

**Table:** 

1	Oral margin reduced, covered by microtrichia (Suppl. material 1: Fig. S37A). Distribution: western, central and southern Africa	***planifacies* species subgroup (Djan et al. 2020)**
–	Oral margin notched, slightly produced forward (as in Suppl. material 1: Fig. S37B)	**2**
2	Metatrochanter with sparse pale pile (Suppl. material 1: Fig. S37C)	**3**
–	Metatrochanter with dense and strong yellow to red brush of pile (Suppl. material 1: Fig. S37D). Distribution: South Africa	***melanocerus* species subgroup (Radenković et al. 2018a)**
3	Apical fourth of tibiae and all tarsi bright yellow; Afrotropical species (Zimbabwe)	***Merodon cuthbertsoni* Curran, 1939**
–	Tarsi partly brown or black; Palaearctic species	***murorum* species group (Vujić et al. 2018b)**

### Key to the species group and unplaced species of the *natans* lineage

**Table:** 

1	Scutum with distinct pollinose ornamentation, vittae and fasciae (Suppl. material 1: Fig. S27D); terga 2–4 with broad pollinose fasciate maculae (Suppl. material 1: Fig. S27A)	***natans* species group**
–	Scutum with indistinct pollinose vittae (Suppl. material 1: Fig. S27E); terga 2–4 without or with narrow pollinose fasciate maculae (Suppl. material 1: Fig. S27B)	***Merodon segetum* Fabricius, 1794**

## Systematic summary

### *Merodon albifrons* lineage

**Diagnosis.** From small to large sized species (7–19 mm) with non-tapering abdomen and a characteristic structure of male genitalia. It is defined by having the mesocoxa pilose posteriorly (> 10 pile) (Suppl. material 1: Fig. S4A), anterior anepisternum with bare area ventral to postpronotum (Suppl. material 1: Fig. S7B), and male genitalia with a biramous anterior surstyle lobe having an apical extension and interior accessory lobe, and a hammer-like lateral sclerite of the aedeagus (except for the *rufus* species group where the lateral sclerite of the aedeagus is not enlarged apically, but with a slightly curved apex) (Suppl. material 2: Fig. S2C: s).

The *albifrons* lineage comprises 65 species (61 described + 4 undescribed) distributed in six species groups (*albifrons*, *constans*, *equestris*, *geniculatus*, *ruficornis*, and *rufus*) and two unplaced species: *M. luteihumerus* Marcos-García, Vujić & Mengual, 2007 and *M. mixtum* Vujić, Radenković & Likov, 2019 (Suppl. material 5: Table S1).

#### 1) *albifrons* species group (Suppl. material 3: Fig. S1A, B)

**Diagnosis**. Small to medium-sized species (7–11 mm); abdominal terga at least partly reddish; terga 2–4 usually each with a pair of pollinose fasciate maculae; male metaleg without projections, calcars or spina, except *M. trochantericus* Costa, 1884 on metatrochanter, metafemur and apex of metatibia (Suppl. material 1: Fig. S34C). Male genitalia with characteristic thorn-like interior accessory lobe on the anterior surstyle lobe, and lateral sclerite of the aedeagus hammer-like with pointed end (Suppl. material 2: Fig. S2C: s).

**Diversity and distribution.** The *albifrons* species group contains eight described species (Suppl. material 5: Table S1) distributed in the Mediterranean Basin, with its highest diversity in the western part.

**Identification.** An identification key to the species of this group is in preparation (Vujić, unpublished).

#### 2) *constans* species group (Suppl. material 3: Fig. S1C, D)

**Diagnosis.** Medium to large-sized species (9–18 mm); posterior part of abdomen (at least tergum 4) covered with golden to reddish-yellow pile (as in Suppl. material 1: Fig. S33A); terga from black (continental species and populations) to reddish (Mediterranean species and populations); terga 2–4 (at least tergum 4) each with a pair of usually elongated pollinose fasciate maculae (Suppl. material 1: Fig. S33A); scutum often with black pile between wing bases; male with tubercle, calcar or lamina on metalegs (on apex of metatibia and ventral margin of metafemur) (Suppl. material 1: Fig. S34I–J). Male genitalia with characteristic rabbit ear-like posterior surstyle lobe, biramous anterior surstyle lobe with moderately developed interior accessory lobe and apical extension, cercus can be with pointed apex (Suppl. material 2: Fig. S1A: c) and lateral sclerite of the aedeagus hammer-like with usually tapering end (Suppl. material 2: Fig. S1F: s).

**Diversity and distribution.** Predominantly northern and eastern Mediterranean distribution, with no representatives on the Iberian Peninsula (Marcos-García et al. 2007). Its highest diversity is in the Caucasus Region and on the Balkan Peninsula.

**Identification.**Vujić et al. (2020a) provided an identification key for 15 species of the *constans* species group and distribution maps.

#### 3) *equestris* species group (Suppl. material 3: Fig. S2A, B)

**Diagnosis.** Medium to large-sized species (11–17 mm) characterised by bumble bee mimicry, with long body pile (Suppl. material 3: Fig. S2A, B); male metatibia with a conspicuous apical calcar (Suppl. material 1: Fig. S34A). Male genitalia with biramous anterior surstyle lobe and with well-developed apical extension curved internally (Suppl. material 2: Fig. S2D: al); lateral sclerite of the aedeagus hammer-like with oval margins (Suppl. material 2: Fig. S2F: s); cercus triangular-shaped (Suppl. material 2: Fig. S2D: c).

**Diversity and distribution.** Three species belong to the *equestris* species group: *M. confusus* Marcos-García, Vujić, Ricarte & Ståhls, 2011, *M. equestris* and *M. flavus* Sack, 1913, all native to South Europe. *Merodon equestris* has been introduced elsewhere, including Japan, North America and New Zealand (Speight 2020).

**Identification.**Marcos-García et al. (2011) provided a taxonomic revision of the group with an identification key.

#### 4) *geniculatus* species group (Suppl. material 3: Fig. S2C, D)

**Diagnosis.** Tergum 2 with reddish lateral maculae; terga 2–4 with distinct pollinose fasciate maculae (Suppl. material 1: Fig. S35B); metatibia in apical third strongly curved, with broad tip (Suppl. material 1: Fig. S34E); metatrochanter in male with a blunt calcar, usually covered with a pile-tuft (Suppl. material 1: Fig. S34D). Male genitalia with biramous anterior surstyle lobe with apical extension more developed, posterior surstyle lobe oval or triangular, cercus with prominence(s) and lateral sclerite of the aedeagus hammer-like with oval margins (Suppl. material 2: Fig. S2I: s).

**Diversity and distribution.** The *geniculatus* species group comprises 11 described species. Marcos-García et al. (2007) described three new species from the Iberian Peninsula (*M. antonioi* Marcos-García, Vujić & Mengual, 2007, *M. crypticus* Marcos-García, Vujić & Mengual, 2007 and *M. longispinus* Marcos-García, Vujić & Mengual, 2007), in addition to the four previously known Iberian taxa (*M. eques* Fabricius, 1805, *M. escorialensis* Strobl in Czerny and Strobl 1909, *M. geniculatus* Strobl in Czerny and Strobl 1909 and *M. teruelensis* van der Goot, 1966). Vujić et al. (2018a) revealed four species from the Eastern Mediterranean: *M. albifasciatus* Macquart, 1842, *M. chalybeatus* Sack, 1913, *M. luteofasciatus* Vujić, Radenković & Ståhls, 2018 and *M. neofasciatus* Ståhls & Vujić, 2018. In addition, there are four undescribed species in the Western Mediterranean (Suppl. material 5: Table S1).

**Identification.** A taxonomic revision of the Eastern Mediterranean species is provided by Vujić et al. (2018a), and the revision for the Western Mediterranean species is in preparation (Vujić, unpublished).

#### 5) *ruficornis* species group (Suppl. material 3: Fig. S3A, B)

**Diagnosis.** Metatrochanter, metafemur and metatibia usually with tubercle, calcar or lamina in the male (Suppl. material 1: Fig. S34F). Male genitalia usually with characteristic hook-like posterior surstyle lobe, biramous anterior surstyle lobe with moderately developed interior accessory lobe and apical extension, cercus without prominences and lateral sclerite of the aedeagus hammer-like with oval margins (Suppl. material 2: Fig. S3C: s). In females, tergum 4 usually with transversal depression (Suppl. material 1: Fig. S36C); terga dark, except tergum 2 with a pair of lateral red-orange maculae; terga 2–4 usually with a pair of white pollinose fasciate maculae; tergum 5 with two small lateral depressions (Suppl. material 1: Fig. S36C); vertex at the level of ocellar triangle shiny black (Suppl. material 1: Fig. S36A).

**Diversity and distribution.** A total of 18 species are recognized in the *ruficornis* species group (Vujić et al. 2012).The group has a predominantly Eastern Mediterranean distribution with a very high level of endemism. Among the 18 taxa (Suppl. material 5: Table S1), 12 are limited-range endemics and are only found in a few mountain areas or in a small part of the total range of the group. Two regions with a high level of endemism are the Anatolian Peninsula and the Caucasus Region.

**Identification.** Distributional data and an identification key for 18 species are provided by Vujić et al. (2012).

#### 6) *rufus* species group (Suppl. material 3: Fig. S3C, D)

**Diagnosis.** In general appearance similar to the members of the *ruficornis* species group. This group comprises black species with bronze reflections that are covered with golden-yellow erect pile, shiny terga and sterna without any trace of pollinosity, and tergum 2 with a pair of small lateral orange maculae (Suppl. material 1: Fig. S33B). Males lack the extensions on the metafemur and metatibia (contrary to the species of the *ruficornis* species group). The male genitalia have biramous anterior surstyle lobe consisting of an interior accessory lobe carrying two spines and protruded apical extension, cercus without prominences (Suppl. material 2: Fig. S3D: c), and with lateral sclerite of the aedeagus curved apically (Suppl. material 2: Fig. S3F: s). In females, the tergum 4 is without a transversal depression (contrary to the female of the *ruficornis* species group), whereas the frons and vertex are shiny, black, and without any pollinosity, with the exception of a narrow line along the eye margin (Suppl. material 1: Fig. S36B).

**Diversity and distribution.** The European *rufus* species group is composed of four species, three of which belong to recently described species from Mediterranean mountains, namely *M. kozufensis* Radenković & Vujić, 2020, *M. olympius* Vujić & Radenković, 2020, and *M. orjensis* Radenković & Vujić, 2020 (Radenković et al. 2020). The fourth species is *M. rufus* Meigen, 1838.

**Identification.**Radenković et al. (2020) recognized this group for the first time and provided a revised identification key.

#### Unplaced species of the *albifrons* lineage

*Merodon luteihumerus* (Suppl. material 3: Fig. S4A, B) is a very distinctive species with yellowish humeri, postalar calli, antennae, tibiae and tarsi of pro- and mesolegs. This is a large species (14–19 mm) with relatively short body pilosity, small basoflagellomere; whitish pile on frons and face, pollinose vittae on scutum, red-yellow lateral maculae on tergum 2 and a pair of pollinose fasciate maculae on terga 2–4. Male genitalia presented in Suppl. material 2: Fig. S3G–I. *Merodon luteihumerus* is distributed in the Iberian Peninsula and Palaearctic Africa.

*Merodon mixtum* (Suppl. material 3: Fig. S4C, D) has a unique combination of characters on the legs, including: apomorphic modifications on pro- and mesotibiae and pro- and metafemora in males (Suppl. material 1: Fig. S32A, C, E), less expressed in females (Suppl. material 1: Fig. S32B, D, F); males with small dens on the metatrochanter; ventral margin of metafemur undulating, with basal tubercle and oval central calcar (Suppl. material 1: Fig. S32E). This is a medium sized species (11–13mm), with fascia of black pile between wing bases; tergum 4 with golden pilosity (Suppl. material 1: Fig. S36D). The species was recently described from the Irano–Anatolian Mountains (Vujić et al. 2019).

### *Merodon aureus* lineage

**Diagnosis.** Posterior part of the mesocoxa pilose (as in Suppl. material 1: Fig. S4A), anterior anepisternum below postpronotum with a pile patch (as in Suppl. material 1: Fig. S7A). Male genitalia with an undeveloped anterior surstyle lobe (as in Suppl. material 2: Fig. S4A: al) and lateral sclerites of the aedeagus very small or absent (as in Suppl. material 2: Fig. S4D: marked with arrow).

The *aureus* lineage contains five species groups: *aureus*, *bombiformis*, *funestus*, *nanus*, and *spinitarsis* with 61 species, 48 of which are described and 13 undescribed (Suppl. material 5: Table S1).

#### 1) *aureus* species group (Suppl. material 3: Figs S5A–D, S6A, B)

**Diagnosis.** Small to medium sized species (8–12 mm) with a short rounded abdomen, a distinct calcar on the metatrochanter in males. Male genitalia have a characteristic posterior surstyle lobe with parallel margins and rounded apex (as in Suppl. material 2: Fig. S4A: pl) and a narrow, elongated, sickle-shaped hypandrium without lateral sclerite of the aedeagus (as in Suppl. material 2: Fig. S4D: marked with arrow).

**Diversity and distribution.** The *aureus* species group comprises a large number of previously known and recently discovered taxa distributed mostly in the Mediterranean Region and South Europe with a high number of local endemics. Šašić et al. (2016) defined six species subgroups within the *aureus* species group: *aureus*, *bessarabicus*, *cinereus*, *chalybeus*, *caerulescens* and *dobrogensis*, and one unplaced species (*M. unguicornis* Strobl in Czerny and Strobl 1909). Each of these species subgroups comprises at least one species complex of cryptic species (Šašić et al. 2019), although they may contain multiple complexes of species such as the *bessarabicus* species subgroup (see Veselić et al. 2017). Recent publications (Veselić et al. 2017; Radenković et al. 2018b; Šašić Zorić et al. 2019; Vujić et al. 2020c) increased the number of known species in the *aureus* species group to 45, including eight undescribed cryptic species of the *ambiguus*, *bessarabicus*, and *sapphous* species complexes (Suppl. material 5: Table S1).

Šašić et al. (2016) defined species complexes as morphologically inseparable species, which can only be resolved by employing an integrative taxonomy approach including different data types such as molecular, geometric morphometry, and ecological niche modelling (ENM). Applying this approach for the *aureus* species group has led to the discovery of previously unknown species complexes. In the *cinereus* species subgroup, Šašić et al. (2016) resolved the *atratus* species complex and found that it consisted of three species, two of which were undescribed. Veselić et al. (2017) provided evidence for the presence of four species complexes in the *bessarabicus* species subgroup. Radenković et al. (2018b) resolved *M. luteomaculatus* Vujić, Ačanski & Šašić, 2018 as a complex of six cryptic species. Additionally, the same approach was used to resolve the *caerulescens* species complex (Šašić et al. 2018). *Merodon dobrogensis* Brădescu, 1982, *M. puniceus* Vujić, Radenković & Pérez-Bañón, 2011 and *M. rojoi* Radenković & Vujić, 2019 are distinct species belonging to the *dobrogensis* species complex within the *dobrogensis* species subgroup (Šašić Zorić et al. 2019).

**Identification.** The identification keys for the various species subgroups have already been published: *aureus* species subgroup (Vujić et al. 2020c), *bessarabicus* species subgroup (Veselić et al. 2017), *cinereus* species subgroup (Šašić et al. 2016), *caerulescens* species subgroup (Šašić et al. 2018) and *dobrogensis* species subgroup (Šašić Zorić et al. 2019). A taxonomic revision of the *chalybeus* species subgroup is in preparation (Vujić, unpublished).

#### 2) *bombiformis* species group (Suppl. material 3: Fig. S6D)

**Diagnosis.** Elongated pedicel, approximately as long as basoflagellomere (relation pedicel: basoflagellomere = 0.9 : 1.1) (Suppl. material 1: Fig. S29A); broad abdomen (Suppl. material 3: Fig. S6D); metafemur with less serrated apicoventral triangular lamina, usually only the apical dens is distinct (as in Suppl. material 1: Fig. S28H); metatrochanter of males smooth, without calcar. Male genitalia with posterior surstyle lobe usually bent (as in Suppl. material 2: Fig. S5D: pl), and hypandrium narrowed medially (as in Suppl. material 2: Fig. S5F: marked with arrow).

**Diversity and distribution.** The *bombiformis* species group consists of six related, though clearly morphologically different species distributed in central and southern Africa, of which three have been described (*M. bombiformis* Hull, 1944, *M. multifasciatus* Curran, 1939, and *M. nasicus* Bezzi, 1915) and three remain undescribed (Suppl. material 5: Table S1).

**Identification.** A taxonomic revision of this species group is in preparation (Vujić, unpublished).

#### 3) *funestus* species group (Suppl. material 3: Fig. S6C)

**Diagnosis.** The *funestus* and the *bombiformis* species groups differ from other species and species groups of the *aureus* lineage by the elongated pedicel, approximately as long, or even longer, than basoflagellomere (Suppl. material 1: Fig. S29A, C) and the small lateral sclerite of the aedeagus (Suppl. material 2: Fig. S5I: s). The *funestus* species group can be distinguished from the *bombiformis* species group by the presence of a calcar on the metatrochanter in males (Suppl. material 1: Fig. S28G), which is absent in the males of the *bombiformis* species group, and a strongly dentate apicoventral triangular lamina on the metafemur in both sexes (Suppl. material 1: Fig. S28F), which is less dentate in the members of the *bombiformis* species group and usually has a distinct apical dens (Suppl. material 1: Fig. S28E, H).

**Diversity and distribution.** The *funestus* species group (Suppl. material 3: Fig. S6C) contains two species, *M. funestus* (Fabricius, 1794) and an undescribed species (Suppl. material 5: Table S1). The species group is distributed in South Europe, Turkey, Israel and Libya.

**Identification.** A taxonomic revision is currently being prepared (Vujić, unpublished).

#### 4) *nanus* species group (Suppl. material 3: Fig. S7B)

**Diagnosis.** Small to medium-sized species (6–12 mm) with a short rounded abdomen. Differs from the members of the *aureus* species group by the absence of a calcar on the metatrochanter in males and abdominal terga with transverse fasciae of pale pile instead of pollinose fasciate maculae (rarely with indistinct pollinosity). Male genitalia with a broad hypandrium (as in Suppl. material 2: Fig. S4G) with the apical part of the aedeagus large, in a form of biramous pliers (as in Suppl. material 2: Fig. S4H). The studied morphological characters show high morphological similarity in all taxa, with the exception of *M. telmateia* Hurkmans, 1987, which has completely pale and unicoloured tarsi (this character clearly separates this taxon from all other members of the *nanus* species group). The five other species can be distinguished by differences in the partly black to brown tarsi and structure of male genitalia (see Vujić et al. 2015; Kočiš Tubić et al. 2018).

**Diversity and distribution.** We recognized six taxa within the *nanus* species group (Suppl. material 5: Table 1). All species from the *nanus* species group are widely distributed across the Anatolian Peninsula, which holds the highest diversity for this species group. Besides the Anatolian Peninsula, this species group occurs to the west including Greece, North Macedonia and Serbia, to the north to the Caucasus Region and Crimean Peninsula, and to the east and south to Syria, Lebanon, Israel and Iran.

**Identification.**Vujić et al. (2015) and Kočiš Tubić et al. (2018) revised the taxonomy of this species group.

#### 5) *spinitarsis* species group (Suppl. material 3: Fig. S7A)

**Diagnosis.** Members of this species group resemble in their overall appearance species of the *nanus* species group, from which they can be easily distinguished by black tibiae and tarsi (mostly pale in the *nanus* species group), and the structure of the male genitalia: hypandrium of male genitalia strongly modified, anfractuous in apical half, with subapical ctenidium and stitched theca (Suppl. material 2: Fig. S4K), and posterior surstyle lobe narrow and pointed (Suppl. material 2: Fig. S4I: pl). Additionally, males of the *spinitarsis* species group have a basoventral lamina on the metatarsus.

**Diversity and distribution.** Only two species are known, *M. spinitarsis* Paramonov, 1929, and an undescribed species (Suppl. material 5: Table S1). *Merodon spinitarsis* is distributed in Greece, Romania and Turkey, while the undescribed species is found in Israel and Palestine (Vujić, unpublished).

**Identification.** A taxonomic revision is currently being prepared (Vujić, unpublished).

### *Merodon avidus-nigritarsis* lineage

**Diagnosis.** Medium to large-sized species (11–20 mm) usually with white pollinose vittae on scutum (Suppl. material 3: Fig. S8C) and white pollinose fasciate maculae on terga (Suppl. material 3: Fig. S8C); anterior anepisternum bare below the postpronotum (Suppl. material 1: Fig. S7B); abdomen elongate, usually narrow and tapering, longer than scutum and scutellum together (Suppl. material 3: Fig. S10C); posterior part of mesocoxa usually without long pile (except in *M. eumerusi* Vujić, Radenković & Likov, 2019) (Suppl. material 1: Fig. S4B); basoflagellomere usually at most twice as long as wide (Suppl. material 1: Fig. S16B); legs without calcar, spina(e) (except in *M. eumerusi*) or tubercle (Suppl. material 1: Fig. S15C). Male genitalia: anterior surstyle lobe usually of rhomboid shape, covered with dense short pile; posterior surstyle lobe usually longer than anterior one; interior accessory lobe of posterior surstyle lobe narrow and long; cercus rectangular, without prominences; hypandrium usually narrow, elongate and sickle-shaped; posterior end of lateral sclerite of the aedeagus tapering; theca of hypandrium usually with a pair of lateral projections; lingula developed (as in Suppl. material 2: Figs S6I, S8F).

The *avidus-nigritarsis* lineage is divided into 10 species groups (*aberrans*, *aurifer*, *avidus*, *clavipes*, *fulcratus*, *italicus*, *nigritarsis*, *pruni*, *serrulatus*, and *tarsatus*) and eight unplaced taxa: *M. auronitens* Hurkmans, 1993, *M. caudatus* Sack, 1913, *M. clunipes* Sack, 1913, *M. crassifemoris* Paramonov, 1925, *M. eumerusi*, *M. hirtus* Sack, 1932, *M. murinus* Sack, 1913 and *M. ottomanus* Hurkmans, 1993. This lineage comprises 79 species, 66 of which are described and 13 undescribed (Suppl. material 5: Table S1).

#### 1) *aberrans* species group (Suppl. material 3: Fig. S8A)

**Diagnosis.** Abdomen elongated and narrow with black shiny terga; terga 2–4 with a pair of white pollinose fasciate maculae (Suppl. material 1: Fig. S11E); metafemur usually long and narrow; hypandrium with very long lingula (Suppl. material 2: Fig. S6C: l).

**Diversity and distribution.** This species group consists of four described species: (*Merodon aberrans* Egger, 1860, *Merodon brevis*Paramonov 1925, *Merodon flavitibius* Paramonov, 1926 and *Merodon hamifer*Sack 1913) and four undescribed species (Suppl. material 5: Table S1) distributed in the Mediterranean and in the east to the Caucasus and Pakistan.

**Identification.** A taxonomic revision including an identification key and descriptions for the four new species is in preparation (Vujić, unpublished).

#### 2) *aurifer* species group (Suppl. material 3: Fig. S8B)

**Diagnosis.** Species with short body pilosity, basoflagellomere 1.2 times as long as wide (Suppl. material 1: Fig. S23A), metafemur covered with short and adpressed pile (Suppl. material 1: Fig. S20B).

**Diversity and distribution.** Besides *M. aurifer* Loew, 1862 distributed in the north Mediterranean and Turkey, the species group consists of at least one additional taxon, an undescribed species from Turkey and Azerbaijan.

**Identification.** A nomenclatural revision of the species group and the description of the new species is in preparation (Vujić, unpublished).

#### 3) *avidus* species group (Suppl. material 3: Fig. S8C)

**Diagnosis.** Species with elongated and tapering abdomen (Suppl. material 1: Fig. 22C), at least tergum 2 with reddish-yellow lateral maculae, and reddish-yellow tarsi (Suppl. material 1: Fig. S20C–D).

**Diversity and distribution.** The *avidus* species group is composed of the *avidus* species complex with four species, and the species *M. femoratus* Sack, 1913 and *M. rutitarsis* Likov, Vujić & Radenković, 2020 (Suppl. material 5: Table S1). This species group is distributed all across Europe, mainly in central and southern zones, and less diverse in the Near and Middle East and in North Africa (Algeria and Libya).

**Identification.** A taxonomic revision with an identification key are presented in Likov et al. (2020).

#### 4) *clavipes* species group (Suppl. material 3: Fig. S9A, B)

**Diagnosis.** Large bumble bee-like species (15–20 mm) with long body pilosity and broad metafemur with long pile (Suppl. material 1: Fig. S12B); basoflagellomere elongated; terga usually covered with pile in different combinations of colours (white, yellow or black) (Suppl. material 3: Fig. S9A, B). Male genitalia with well-defined and large anterior and posterior surstyle lobes (Suppl. material 2: Fig. S7A: al, pl).

**Diversity and distribution.** The *clavipes* species group contains four species (Suppl. material 5: Table 1) distributed in the Mediterranean Region and up to Iran in the east.

**Identification.** A taxonomic revision of this species group is under preparation (Vujić, unpublished).

#### 5) *fulcratus* species group (Suppl. material 3: Fig. S9C)

**Diagnosis.** They are small sized species (5–9 mm) with metallic shiny bodies; scutum and terga strongly punctate, without or with very weak pollinose areas (Suppl. material 1: Fig. S17A, B); metafemur with very small apical triangular lamina apicoventrally (Suppl. material 1: Fig. S12A). Males of this species group are clearly separated from other species groups of the *avidus-nigritarsis* lineage by distinctly dichoptic eyes and lack of ctenidium at hypandrium.

**Diversity and distribution.** Two species are known, *M. dichopticus* Stackelberg, 1968 from Turkey and *M. fulcratus* (Becker, 1913) from Iran.

**Identification.** A taxonomic revision of this group is under preparation (Vujić, unpublished).

#### 6) *italicus* species group (Suppl. material 3: Fig. S8D)

**Diagnosis.** Species with elongate basoflagellomere, at least 2.7 times as long as wide (Suppl. material 1: Fig. S13A) and quadratic posterior surstyle lobe (Suppl. material 2: Fig. S7D: pl).

**Diversity and distribution.** Two species share these morphological features and belong to this species group: *M. italicus* Rondani, 1845 recorded from most of the Mediterranean and *M. erivanicus* Paramonov, 1925 distributed from Croatia to Azerbaijan and Israel.

**Identification.** A taxonomic revision of this group is in preparation (Vujić, unpublished).

#### 7) *nigritarsis* species group (Suppl. material 3: Fig. S10C)

**Diagnosis.** Species with elongate, narrow and tapering abdomen, tarsi dark brown/black dorsally and partly orange ventrally. Male genitalia: anterior surstyle lobe more or less rhomboid shape (Suppl. material 2: Fig. S8D: al), except in *alagoezicus* species subgroup where the anterior surstyle lobe is transformed into a narrow, elongate, strongly curved projection (Suppl. material 2: Fig. S8A: al); hypandrium with a pair of apical thorns on the ventral margin directed backwards but often with a pair of lateral projections near the base and well-developed lingula (Suppl. material 2: Fig. S8F: l).

**Diversity and distribution.** The *nigritarsis* species group includes 17 species revised in Vujić et al. (2013) and Likov et al. (2020) grouped into two species subgroups. Six of them belong to the *alagoezicus* species subgroup (*M. alagoezicus* Paramonov, 1925, *M. hakkariensis* Vujić & Radenković in Vujić et al. 2013, *M. lucasi* Hurkmans, 1993, *Merodon nitidifrons* Hurkmans, 1993, *M. satdagensis* Hurkmans, 1993 and *M. schachti* Hurkmans, 1993) and the other 11 species are members of the *nigritarsis* species subgroup (Suppl. material 5: Table S1). The *nigritarsis* species group comprises taxa with a mainly mountainous distribution, mostly on the Balkan, Anatolian, Apennine and Iberian Peninsulas, in central Europe as well as the Middle and Near East (Likov et al. 2020).

**Identification.** A taxonomic revision is provided by Vujić et al. (2013) and Likov et al. (2020).

#### 8) *pruni* species group (Suppl. material 3: Fig. S10A, B)

**Diagnosis.** Medium to large-sized species (10–18 mm) characterised by short body pilosity (scutum and abdomen); short basoflagellomere, as long as broad (Suppl. material 1: Fig. S23B); metafemur dorsally and ventrally covered with medium long outstanding pile (Suppl. material 1: Fig. S20G); and metatrochanter with distinct calcar (Suppl. material 1: Fig. S20H).

**Diversity and distribution.** Four species belong to this species group: *M. cupreus* Hurkmans, 1993, *M. pallidus* Macquart, 1842 and *M. pruni* Rossi, 1790 and one undescribed taxon from Israel. *Merodon pruni* is distributed in most of the Mediterranean Basin, but the other two described species are more allocated to the east, from Turkey to Israel and Pakistan.

**Identification.** A taxonomic revision of this group is in preparation (Vujić, unpublished).

#### 9) *serrulatus* species group (Suppl. material 3: Fig. S9D)

**Diagnosis.** Species with characteristic basolateral protrusion on the posterior surstyle lobe at outer surface (Suppl. material 2: Fig. S9G: marked with arrow); legs mostly black; terga black, tergum 2 usually with a pair of reddish orange lateral maculae; metafemur usually with shorter pilosity ventrally, less than width of metafemur (Suppl. material 1: Fig. S14A); basoflagellomere usually narrow and elongated, dark brown, two times longer as pedicel. They are medium-large (11–15 mm) species with a dark scutum and white pollinose fasciate maculae (at least in females) on the dark olive brown terga 2–4 (Suppl. material 1: Fig. S22D).

**Diversity and distribution.** This species group includes 13 species (Vujić et al. 2020b). *Merodon serrulatus* Wiedemann in Meigen, 1822 is the species of the genus *Merodon* with the largest distributional range being distributed from the Iberian Peninsula in the south-west, along the Mediterranean and Balkan Peninsula, through Turkey and southern Russia to Siberia and Mongolia in the north-east. Other species of the *serrulatus* species group can be found at the edges of this distributional range, albeit with a much more restricted distribution (see Vujić et al. 2020b).

**Identification.** This species group is revised by Vujić et al. (2020b), who gave descriptions of seven new species and provided an identification key.

#### 10) *tarsatus* species group (Suppl. material 3: Fig. S11A, B)

**Diagnosis.** Small to medium sized species (8–14 mm) with usually expanded basotarsomere of metatarsus (Suppl. material 1: Figs S12C, S18B) and/or with strong setae ventrally (Suppl. material 1: Fig. S12D); males with sternum 4 medially clearly divided with membranous structure and lateral tubercles or laminate extensions (Suppl. material 1: Fig. S11C); sternum 4 from lateral view usually fin-form (Suppl. material 1: Fig. S11D).

**Diversity and distribution.** The *tarsatus* species group consists of seven described and six undescribed species (Suppl. material 5: Table S1). This group of species is geographically restricted to the Near and Middle East, and Central Asia.

**Identification.**Vujić et al. (2019) and Likov et al. (2020) mentioned this group of species but did not give diagnostic features. A taxonomic revision of the *tarsatus* species group is in preparation (Vujić, unpublished).

### Unplaced species of *avidus-nigritarsis* lineage

*Merodon auronitens* (Suppl. material 3: Fig. S10D) is a species with dark terga, basoflagellomere with concave dorsal margin (Suppl. material 1: Fig. S13E); posterior surstyle lobe with triangular basal extension (Suppl. material 2: Fig. S10A: marked with arrow); in females terga 2–4 strongly punctate; posterior half of tergum 4 with longer whitish, mostly adpressed pile (Suppl. material 1: Fig. S19D). Species has distribution in Turkey and Israel.

*Merodon caudatus* (Suppl. material 3: Fig. S11C, D) belongs to species with partly reddish terga and unique modification of legs among *avidus-nigritarsis* lineage: metatibia twisted medially in apical half, basotarsomere of metatarsus strongly modified (Suppl. material 1: Fig. S20A); tarsomere of mesotarsus with strong, black lateral setae (Suppl. material 1: Fig. S24B). This species is known from Israel and Palestine.

*Merodon clunipes* (Suppl. material 3: Fig. S12A, B) is a species with broad metatibiae and dark terga, and has clear apomorphic diagnostic characters, including antennal shape: fossette large, extended from dorsal side to outer, covering half of lateral surface (Suppl. material 1: Fig. S13D), and the characteristic shape of the posterior surstyle lobe with the apical hump directed toward cercus (Suppl. material 2: Fig. S10G: marked with arrow). This species has a North Mediterranean distribution.

*Merodon crassifemoris* (Suppl. material 3: Fig. S12C, D) is a taxon with tubercle on the face below the antenna (Suppl. material 1: Figs S21A, S25B), and a hook-like posterior surstyle lobe (Suppl. material 2: Fig. S10J: pl) unique among all other taxa of the *avidus-nigritarsis* lineage. It was recently revised and excluded from *M. nigritarsis* group (Likov et al. 2020). The distribution of *M. crassifemoris* extends from the eastern Balkans through the Anatolian Peninsula as far as Ukraine and Azerbaijan.

*Merodon eumerusi* (Suppl. material 3: Fig. S13A) possesses a line of spinae on the inner side of the apical quarter of metafemur (Suppl. material 1: Fig. S5A), representing a unique character that is absent in all other species of the genus; male genitalia (Suppl. material 2: Fig. S11A–C) similar to *M. ottomanus* (Suppl. material 2: Fig. S11G–I). Differs from other known species of the *M. avidus-nigritarsis* lineage in having 1–4 fine pile (usually one) on the posterior side of the mesocoxa. In males, the basoflagellomere is elongated with an angular apex, bearing a very large outer fossette and a second inner fossette (Suppl. material 1: Fig. S10A, B), which are absent in almost all other species of the genus except *M. serrulatus* (Vujić et al. 2020b). This species is recently described from high mountain ranges in Tajikistan, Uzbekistan and Kyrgyzstan (Vujić et al. 2019).

*Merodon hirtus* (Suppl. material 3: Fig. S13B) belongs to species with dark terga, males with posterior surstyle lobe divided in two branches (Suppl. material 2: Fig. S9J: pl); eyes slightly dichoptic, distance between eyes about two facets wide (Suppl. material 1: Fig. S11B); in females terga 2–4 finely punctate; posterior half of tergum 4 with shorter, mostly black and adpressed pile (Suppl. material 1: Fig. S19E). This is an extreme eastern Mediterranean species with a range extending from Turkey to Iran and Israel, as well as Cyprus.

*Merodon murinus* (Suppl. material 3: Fig. S13C) is a medium to large-sized species (12–15 mm) with yellow tarsi, and tibiae mostly yellowish, except medially where brown (Suppl. material 1: Fig. S15F); basotarsomere of metatarsus elongated, three times as long as wide (Suppl. material 1: Fig. S15F); basoflagellomere elongated, 2.5 times as long as wide (Suppl. material 1: Fig. S16C); males with eye contiguity very short, approximately four to five facets long (Suppl. material 1: Fig. S21B); male genitalia with elongated compact surstyle lobe (Suppl. material 2: Fig. S11D: pl). *Merodon murinus* is a rare species recorded from Turkey and Turkmenistan.

*Merodon ottomanus* (Suppl. material 3: Fig. S13D) is a species with dark abdomen, reddish-yellow basoflagellomere and yellow tarsi of metaleg (at least basotarsomere); posterior surstyle lobe large, rounded, while anterior surstyle lobe small (Suppl. material 2: Fig. S11G: al, pl). This species has a fragmented distribution including the Iberian Peninsula, Peloponnesus (Greece), Turkey and Iran. It will be taxonomically revised in the future (Vujić, unpublished).

### *Merodon desuturinus* lineage

**Diagnosis.** The specific shape of the lateral sclerite of the aedeagus (gradually tapered, with the tip curved downwards) is the main synapomorphic character that connects all species from the group (as in Suppl. material 2: Fig. S12I: s). Moreover, the species in this species group have pile on posterior side of mesocoxa; a curved distal prolongation of anterior surstyle lobe (as in Suppl. material 2: Fig. S12E: al); basoflagellomere less than two times as long as wide (Suppl. material 1: Fig. S6B); scutum without pollen or with less distinct pollinose longitudinal vittae (Suppl. material 1: Fig. S6D); wing microtrichose between veins R_1_ and RS (Suppl. material 1: Fig. S9A); postpronotum usually brown or yellow-reddish; pilosity on lateral side of tergum 4 in female long, medially short and mostly adpressed (Suppl. material 1: Fig. S8A). The *desuturinus* lineage is closely related to the *albifrons* lineage, which was named *albifrons+desuturinus* clade in Radenković et al. (2018a).

The *desuturinus* lineage contains the Afrotropical *melanocerus* species group with two species subgroups (*melanocerus* and *planifacies*) and the species *M. cuthbertsoni* Curran, 1939 (Radenković et al. 2018a; Djan et al. 2020) (Suppl. material 3: Fig. S14C), and the Palaearctic *murorum* species group with four species (Vujić et al. 2018b). The *desuturinus* lineage comprises 14 described and 10 still undescribed species (Suppl. material 5: Table S1).

#### 1) *melanocerus* species group (Suppl. material 3: Fig. S14A)

**Diagnosis.** Species with patch of dense yellow pile (dense and strong yellow to red brush of pile) on metatrochanter (Suppl. material 1: Fig. S37D). The *melanocerus* species subgroup has the oral margin notched, slightly produced forward (Suppl. material 1: Fig. S37B) and the *planifacies* species subgroup has the oral margin reduced, covered with microtrichia (Suppl. material 1: Fig. S37A). *Merodon cuthbertsoni*, with an unclear position within the species group, has apical fourth of tibiae and all tarsi bright yellow.

**Diversity and distribution.** Distribution of the *melanocerus* species subgroup is limited to South Africa, while the *planifacies* species subgroup has broader range: western, central and southern Africa. *Merodon cuthbertsoni* occurs in Zimbabwe.

**Identification.** Recent revision of the *melanocerus* species subgroup (Radenković et al. 2018a) resulted in the delimitation of five species: *M. capensis* Hurkmans, 2018, *M. commutabilis* Radenković & Vujić, 2018, *M. drakonis* Vujić & Radenković, 2018, *M. flavocerus* Hurkmans, 2018 and *M. melanocerus* Bezzi, 1915. Part of the *planifacies* species subgroup was the subject of a recent molecular analysis, which supported the monophyly of the subgroup (Djan et al. 2020). According to their integrative approach, three species are found within the *planifacies* species subgroup in South Africa: *M. planifacies* Bezzi, 1915, and two species of the *capi* species complex characterized by smooth thecal ridge in male genitalia, namely *M. capi* Vujić & Radenković, 2020 and *M. roni* Radenković & Vujić, 2020. The fourth known species from the species *planifacies* subgroup, *M. stevensoni* Curran, 1939, was described based on one female from Zimbabwe, and its taxonomic status remains unclear until the discovery of additional material, especially male specimens (Djan et al. 2020). Within the *planifacies* species subgroup, populations with folded thecal ridge of hypandrium in male genitalia could represent a group of geographically isolated species, which needs additional taxonomic research based on integrative approach (Djan et al. 2020). Ten undescribed species are already recognized (Suppl. material 5: Table S1) and descriptions are in preparation (Vujić, unpublished).

#### 2) *murorum* species group (Suppl. material 3: Fig. S14B)

**Diagnosis.** Species without patch of dense yellow pile (dense and strong yellow to red brush of pile) on metatrochanter.

**Diversity and distribution.** This species group includes four endemo-relicts: *M. cabanerensis* Marcos-García, Vujić & Mengual, 2007, known only from a restricted area in central Spain and Morocco; *M. desuturinus* Vujić, Šimić & Radenković, 1995 (Suppl. material 3: Fig. S14B) localized on high mountains in the Balkans; *M. murorum* Fabricius, 1794 from North-West Africa; and *M. neolydicus* Vujić, 2018, present in several countries in the Eastern Mediterranean (Greece, Turkey, Syria, Lebanon, Israel).

**Identification.**Vujić et al. (2018b) recently revised this species groups and provided an identification key for the *desuturinus* lineage, including the *murorum* species group.

### *Merodon natans* lineage

**Diagnosis.** Species with few pile on posterior side of mesocoxa; pile on anterior anepisternum reduced; anterior lobe of surstylus well developed, oval, rounded, pilose, without curved distal prolongation (Suppl. material 2: Fig. S13A: al); basoflagellomere elongated, two times as long as wide, narrowed in apical third (Suppl. material 1: Fig. S27C); scutum usually with five well-defined pollinose longitudinal vittae (Suppl. material 1: Fig. S27D).

The *natans* lineage contains the *natans* species group with three described species (Radenković et al. 2011), one undescribed species (Vujić et al. in prep.), and *M. segetum* Fabricius, 1794 (Suppl. material 5: Table S1). Species belonging to the *natans* lineage have Mediterranean distribution, except for one population of *M. calcaratus* (Fabricus, 1794) recorded in Kenya (Vujić, unpublished).

#### 1) *natans* species group (Suppl. material 3: Fig. S15A)

**Diagnosis.** Small to medium-sized species (8–13 mm) with distinct pollinose ornamentation, vittae and fasciae, on scutum (Suppl. material 1: Fig. S27D); terga 2–4 with broad pollinose fasciate maculae (Suppl. material 1: Fig. S27A).

**Diversity and distribution.** The *natans* species group is distributed around the Mediterranean Basin and there is one isolated record in Kenya (Vujić, unpublished).

**Identification.** A taxonomic revision of this species group is in preparation (Vujić, unpublished).

### Unplaced species of the *natans* lineage

*Merodon segetum* is a large species (14–17 mm) (Suppl. material 3: Fig. S15B), with the scutum with indistinct pollinose vittae (Suppl. material 1: Fig. S27E), and terga 2–4 without or with narrow pollinose fasciate maculae (Suppl. material 1: Fig. S27B). This is a western Mediterranean species occurring in the south of Spain, Algeria, Tunisia and Libya.

## Discussion

Out of 194 described species (234 in total including undescribed taxa), 180 (209) species are distributed in the Palaearctic Region and 14 (27) are known from the Afrotropical Region. Three lineages (*aureus*, *desuturinus*, and *natans*) have representatives in both the Afrotropical and the Palaearctic Regions. The Afrotropical *melanocerus* species group of the *desuturinus* lineage and the *bombiformis* species group of the *aureus* lineage are exclusive to the Afrotropical Region, while all other species groups belong to Palaearctic fauna.

The *albifrons* lineage, with 65 species (61 described taxa), contains six species groups (*albifrons*, *constans*, *equestris*, *geniculatus*, *ruficornis*, and *rufus*) and two unplaced taxa.

The *aureus* lineage, with 61 species (48 described), contains five species groups (*aureus*, *bombiformis*, *funestus*, *nanus*, and *spinitarsis*).

The *avidus-nigritarsis* lineage, with 79 species (67 described), is divided into 10 species groups (*aberrans*, *aurifer*, *avidus*, *clavipes*, *fulcratus*, *italicus*, *nigritarsis*, *pruni*, *serrulatus*, and *tarsatus*) and eight unplaced species.

The *desuturinus* lineage, with 24 species (14 described), contains two species groups: the Afrotropical *melanocerus* species group with two species subgroups (*melanocerus* and *planifacies*) and the unplaced species *M. cuthbertsoni*; and the Palaearctic *murorum* species group with four species.

The *natans* lineage contains the *natans* species group, with four species (three described), and the unplaced species *M. segetum*.

At present and based on our results, the regions with the highest species richness are the Mediterranean Peninsulas: Iberian, Balkan and especially Anatolian. Certain areas in the Palaearctic (regions of Pakistan, Central Asia and eastern part of the Middle East) and Afrotropical Regions (Central and Eastern Africa) have been under-sampled and they need additional collecting efforts. Central Asia and Pakistan are characterised by numerous endemics with potential significance to understand the evolutionary scenario of the genus *Merodon*. Finally, the genetic diversity is extremely high in the *aureus* species group and more taxonomic research still needs to be done in this species group and some others, like the *ruficornis*, *avidus* and *equestris* species groups.

## Supplementary materials

Supplementary material 1 Figures S1–S37: Figures of morphological characters Data type multimedia Explanation note **Figure S1.** Head of male, posterior view. **A***Platynochaetus setosus* (Fabricius, 1794), hypostomal bridge marked with arrow **B***Sericomyia silentis* (Harris, 1776). Scale bar: 0.5 mm. **Figure S2.** Parts of thorax of male, lateral view. **A***Merodon megavidus* Vujić & Radenković, 2016, tubercle **B***Eristalinus megacephalus* (Rossi, 1794), tubercle **C***Merodon megavidus*, anepimeron **D***Sericomyia silentis*, anepimeron. **A, B** tubercle marked with arrow **C, D** dorsomedian part of anepimeron marked with arrow. Scale bar: 0.5 (**A–C**); 1 mm (**D**). **Figure S3. A, B** Wing of male, dorsal view **C–D** Antenna of male, lateral view. **A**Merodon aff. nasicus, short vein marked with asterisk **B***Sericomyia silentis***C***Merodon ottomanus*, fossette marked with arrow **D***Microdon analis* (Macquart, 1842). **A, B** outer angle between R_4+5_ and M_1_ marked with arrow. Scale bar: 0.5 mm (**A, B, D**); 0.2 mm (C). **Figure S4.** Metacoxa of male. **A***Merodon albifrons* Meigen, 1822, lateral view **B***M. nigritarsis* Rondani, 1845, lateral view. **A, B** pile marked with arrow. Scale bar: 0.25 mm. **Figure S5. A** Metafemur of *Merodon eumerusi*, male, lateroventral view, row of spinae marked with arrow **B** Mesocoxa of *M. segetum*, male, lateral view, pile marked with arrow. Scale bar: 1 mm. **Figure S6. A, B** Antenna of male, lateral view **C, D** Thorax of male, dorsal view. **A, C***Merodon natans* (Fabricius, 1794) **B, D***M. desuturinus*. Scale bar: 1 mm. **Figure S7.** Thorax (anepisternum) of male, lateral view. **A***Merodon geniculatus***B***M. legionensis* Marcos-García, Vujić & Mengual, 2007. **A, B** pile marked with arrow. **Figure S8.** Abdomen of female, lateral view. **A***Merodon desuturinus***B***M. aureus* Fabricius, 1805. **A**, pile marked with arrow. Scale bar: 1 mm. **Figure S9.** Part of wing of male, dorsal view. **A***Merodon desuturinus***B***M. albifrons*. **A–B** microtrichia marked with arrow. Scale bar: 1 mm. **Figure S10.** Antenna of *Merodon eumerusi*, male, lateral view. **A** outer side **B** inner side. Scale bar: 1 mm. **Figure S11. A, B** Eye contiguity of male, anterior view **C, D** Sternum 4 of male, dorsal (**C**) and lateral (**D**) view **E** Abdomen of male, dorsal view. **A***Merodon fulcratus***B***M. hirtus***C, D***M. tarsatus***E***M. aberrans*. **C, D** membranous structure and laminate extension marked with arrow. Scale bar: 1 mm (**A, B**); 2 mm (**C–E**). **Figure S12.** Parts of male metaleg, lateral view. **A***Merodon fulcratus*, metaleg **B***M. clavipes* (Fabricius, 1781), metafemur and metatibia **C***M. tarsatus*, metatarsus **D***M. oidipous* Hurkmans, 1993, metatarsus. **C, D** setae marked with arrrow. Scale bar: 2 mm (**A, B**); 0.5 mm (**C, D**). **Figure S13.** Male antenna, lateral view. **A***Merodon italicus***B***M. serrulatus***C***M. ottomanus***D***M. clunipes***E***M. auronitens*. Scale bar: 2 mm. **Figure S14.** Parts of male metaleg, lateral view. **A***Merodon serrulatus*, metafemur and metatibia **B***M. ottomanus*, metaleg **C***M. clunipes*, metafemur **D***M. clunipes*, metatarsus. Scale bar: 2 mm. **Figure S15.** Parts of female metaleg, lateral view. **A***Merodon clunipes*, metafemur and metatibia **B***M. ottomanus*, metaleg **C***M. clavipes*, metafemur **D***M. fulcratus*, metafemur **E***M. serrulatus*, metafemur **F***M. murinus*, metatibia and metatarsus. **D**triangular lamina marked with arrow. Scale bar: 2 mm. **Figure S16.** Female antenna, lateral view. **A***Merodon clunipes***B***M. clavipes***C***M. murinus*. Scale bar: 2 mm. **Figure S17. A** Female thorax, dorsal view **B–D** Abdomen of female, dorsal view. **A, B***Merodon fulcratus***C***M. ottomanus***D***M. aberrans*. Scale bar: 2 mm. **Figure S18.** Parts of female metaleg, lateral view. **A***Merodon aberrans*, metaleg **B***M. tarsatus*, metatarsus **C***M. hirtus*, metatarsus **D***M. auronitens*, metatarsus. **B** setae marked with arrow **C-D** tarsomerae marked with arrow. Scale bar: 1 mm (**A**); 2 mm (**B–D**). **Figure S19.** Abdomen of female. **A***Merodon oidipous*, dorsal view **B***M. tarsatus*, dorsal view **C***M. auronitens*, dorsal view **D***M. auronitens*, lateral view **E***M. hirtus*, lateral view. Scale bar: 2 mm. **Figure S20.** Parts of male metaleg. **A***Merodon caudatus*, metatibia and metatarsus, lateral view **B***M. aurifer*, metatrochanter and metafemur, lateral view **C***M. avidus* (Rossi, 1790), metatarsus, dorsal view **D***M. avidus*, metatarsus, ventral view **E***M. nigritarsis*, metatarsus, dorsal view **F***M. nigritarsis*, metatarsus, ventral view **G***M. pruni*, part of metafemur, lateral view **H***M. pruni*, metatrochanter, lateral view. **B** calcar marked with arrow. Scale bar: 0.5 mm (**A–B**); 1mm (**C–G**); 0.25 mm (**H**). **Figure S21. A***Merodon crassifemoris*, male, head, lateral view **B***M. murinus*, male, eye contiguity, anterior view **C***M. nigritarsis*, male, eye contiguity, anterior view. **A** bulge marked with arrow. Scale bar: 1 mm. **Figure S22.** Abdomen, dorsal view. **A***Merodon aurifer***B***M. pruni***C***M. avidus***D***M. serrulatus*. **A–C** male **D** female. Scale bar: 2 mm. **Figure S23.** Male antenna, lateral view. **A***Merodon aurifer***B***M. pruni***C***M. nigritarsis*. Scale bar: 1 mm (**A, C**); 0.5 mm (**B**). **Figure S24.** Parts of metaleg of female. **A***Merodon caudatus*, metafemur and metatibia, lateral view **B***M. caudatus*, metatarsus, dorsal view **C***M. pruni*, metatrochanter, metafemur and metatibia **D***M. crassifemoris*, metatrochanter, lateral view **E***M. aurifer*, metafemur and metatibia. **C** metatrochanter marked with arrow. Scale bar: 1 mm (**A, C, E**); 0.5 mm (**B**); 0.75 mm (**D**). **Figure S25. A***Merodon murinus*, female, head, dorsal view **B***M. crassifemoris*, female, head, dorsolateral view **C***M. italicus*, female, abdomen, dorsal view. **B** bulge marked with arrow. Scale bar: 1 mm (**A–B**); 2 mm (**C**). **Figure S26.** Antenna of female, lateral view. **A***Merodon pruni***B***M. crassifemoris***C***M. aurifer***D***M. italicus***E***M. avidus*. Scale bar: 0.5 mm (**A–C, E**); 1 mm (**D**). **Figure S27. A***Merodon natans*, male, abdomen, dorsal view **B***M. segetum*, male, abdomen, dorsal view **C***M. natans*, male, antenna, lateral view **D***M. natans*, male, thorax, dorsal view **E***M. segetum*, male, thorax, dorsal view. Scale bar: 2 mm (**A–C**); 1 mm (**D**); 0.5 mm (**E**). **Figure S28.** Parts of metaleg. **A***Merodon bombiformis*, male, metatrochanter, lateral view **B***M. aureus*, male, metatrochanter, lateral view **C***M. spinitarsis*, female, metatarsus, dorsal view **D***M. nanus* (Sack, 1931), female, metatarsus, dorsal view **E***M. bombiformis*, female, metafemur, lateral view **F***M. funestus*, female, metafemur, lateral view **G***M. funestus*, male, metafemur and metatrochanter, lateral view **H***M. bombiformis*, male, metafemur and metatrochanter, lateral view. **B** calcar on the metatrochanter marked with arrow **E** triangular lamina marked with arrow. Scale bar: 2 mm. **Figure S29.** Antenna, lateral view. **A***Merodon bombiformis*, male **B***M. nanus*, male **C***M. funestus*, male **D***M. aureus*, male **E***M. funestus*, female **F***M. aureus*, female. Scale bar: 2 mm. **Figure S30.** Abdomen. **A***Merodon funestus*, male, dorsolateral view **B***M. aureus*, male, lateral view **C***M. nanus*, female, dorsal view **D***M. aureus*, female, dorsal view **E***M. spinitarsis*, female, lateral view **F***M. nanus*, female, lateral view. Scale bar: 2 mm. **Figure S31.***Merodon luteihumerus*, male. **A** head, anterior view **B** thorax, dorsolateral view. Scale bar: 2 mm. **Figure S32.***Merodon mixtum*, legs, lateral view. **A** proleg, male **B** proleg, female **C** mesoleg, male **D** mesoleg, female **E** metaleg, male **F** metaleg, female. Scale bar: 1 mm. **Figure S33. A–C** Abdomen of male **D** Metatibia. **A***Merodon gudaurensis* Portschinsky, 1877, dorsal view **B***M. rufus*, dorsal view **C***M. rufus*, lateral view **D***M. albifrons*, lateral view. Scale bar: 2 mm. **Figure S34.** Parts of metaleg, lateral view. **A***Merodon equestris*, male, metatibia **B***M. rufus*, male, metaleg **C***M. trochantericus*, male, metaleg **D***M. albifasciatus*, male, metatrochanter **E***M. albifasciatus*, male, metatibia **F***M. trebevicensis* Strobl, 1900, male, metatrochanter, metafemur and metatibia **G***M. ruficornis* Meigen, 1822, female, metatibia **H***M. albifasciatus*, female, metatibia **I***M. gudaurensis*, male, metafemur **J***M. gudaurensis*, male, metatibia. **E** apicomedial carina marked with arrow **F** ventral tubercle on metafemur and apicolateral process on metatibia marked with arrow. Scale bar: 0.5 mm (**A, B**); 2 mm (**C**); 1 mm (**D–E**); 2 mm (**F**); 2 mm (**G–H**); 2 mm (**I-J**). **Figure S35. A***Merodon albifasciatus*, female, thorax, dorsal view **B***M. albifasciatus*, female, abdomen, dorsal view **C***M. albifrons*, female, abdomen, dorsal view. Scale bar: 2 mm. **Figure S36. A, B** Head of female, dorsal view **C, D** Tip of abdomen, dorsal view. **A***Merodon ruficornis***B***M. rufus***C***M. ruficornis*, female **D***M. mixtum*, male. **B** pollinosity along eye margin marked with arrow **C** transversal depression on tergum 4 and lateral depressions on tergum 5 marked with arrow. Scale bar: 2 mm. **Figure S37. A–B** Head of male, lateral view **C–D** Metatrochanter of male, lateral view. **A***Merodon planifacies***B***M. neolydicus***C***M. desuturinus***D***M. draconis* Vujić & Radenković, 2018. Scale bar: 1 mm (**A–B**); 2 mm (**C–D**). https://binary.pensoft.net/file/531631This dataset is made available under the Open Database License (http://opendatacommons.org/licenses/odbl/1.0/). The Open Database License (ODbL) is a license agreement intended to allow users to freely share, modify, and use this Dataset while maintaining this same freedom for others, provided that the original source and author(s) are credited.Ante Vujić, Snežana Radenković, Laura Likov, Sanja Veselić

Supplementary material 2 Figures S1–S13: Figures of male genitalia Data type multimedia Explanation note **Figure S1.** Male genitalia. **A–C***Merodon constans* (Rossi, 1794) **D–F***M. chrysotrichos* Vujić, Radenković & Likov, 2020 **G–I***M. triangulum* Vujić, Radenković & Hurkmans, 2020. **A, B, D–E, G, H** epandrium **C, F, I** hypandrium. **A, C, D, F, G, I** lateral view **B, E, H** ventral view. Abbreviations: c-cercus, s-lateral sclerite of the aedeagus. Scale bar: 0.5 mm. **Figure S2**. Male genitalia. **A–C***Merodon albifrons* Meigen, 1822 **D–F***M. equestris***G–I***M. albifasciatus*. **A–B, D–E, G–H** epandrium **C, F, I** hypandrium. **A, C, D, F, G, I** lateral view **B, E, H** ventral view. Abbreviations: al-anterior surstyle lobe, c-cercus, s-lateral sclerite of the aedeagus. Scale bar: 0.4 mm (**A–C**); 0.2 mm (**D–F**); 0.4 mm (**G–I**). **Figure S3.** Male genitalia. **A–C***Merodon ruficornis* Meigen, 1822 **D–F***M. rufus***G–I***M. luteihumerus***J–L***M. mixtum*. **A–B, D–E, G–H, J–K** epandrium **C, F, I, L** hypandrium. **A, C, D, F, G, I, J, L** lateral view **B, E, H, K** ventral view. Abbreviations: c-cercus, s-lateral sclerite of the aedeagus. Scale bar: 0.4 mm (**A–C**); 0.25 mm (**D–F**); 0.2 mm (**G–I**); 0.5 mm (**J–L**). **Figure S4.** Male genitalia. **A–D***Merodon aureus* Fabricius, 1805 **E–H***M. nanus* (Sack, 1931) **I–K***M. spinitarsis*. **A, B, E, F, I, J** epandrium **C, G, K** hypandrium **D, H** part of aedeagus. **A, C, D, E, G, I, K** lateral view **B, F, H, J** ventral view. Abbreviations: al-anterior surstyle lobe, pl-posterior surstyle lobe. **D** place of lateral sclerite of the aedeagus marked with arrow. Scale bar: 0.25 mm (**A–D**); 0.25 mm (**E–H**); 0.5 mm (**I–K**). **Figure S5.** Male genitalia. **A–C***Merodon bombiformis***D–F***M. nasicus***G–I***M. funestus*. **A–B, D–E, G–H** epandrium **C, F, I** hypandrium. **A, C, D, F, G, I** lateral view **B, E, H** ventral view. Abbreviations: pl-posterior surstyle lobe, s-lateral sclerite of the aedeagus. **C, F** medially narrowed hypandrium marked with arrow. Scale bar: 0.5 mm. **Figure S6.** Male genitalia. **A–C***Merodon aberrans***D–F***M. aurifer***G–I***M. avidus* (Rossi, 1790) **J–L***M. rutitarsis*. **A–B, D–E, G–H, J–K** epandrium **C, F, I, L** hypandrium. **A, C, D, F, G, I, J, L** lateral view **B, E, H, K** ventral view. Abbreviations: l-lingula. Scale bar: 0.2 mm (**A–C, G–I**); 0.5 mm (**D–F, J–L**). **Figure S7.** Male genitalia. **A–C***Merodon clavipes* (Fabricius, 1781) **D–F***M. italicus***G–I***M. dichopticus***J–K***M. fulcratus*. **A–B, D–E, G–H, J–K** epandrium **C, F, I, L** hypandrium. **A, C, D, F, G, I, J, L** lateral view **B, E, H, K** ventral view. Abbreviations: al-anterior surstyle lobe, pl-posterior surstyle lobe. **C, K** place of ctenidium marked with arrow. Scale bar: 0.2 mm (**A–F, J–L**); 0.5 mm (**G–I**). **Figure S8.** Male genitalia. **A–C***Merodon alagoesicus* Paramonov, 1925 **D–F***M. nigritarsis* Rondani, 1845 **G–I***M. obstipus* Vujić, Radenković & Likov, 2020. **A–B, D–E, G–H** epandrium **C, F, I** hypandrium. **A, C, D, F, G, I** lateral view **B, E, H** ventral view. Abbreviations: al-anterior surstyle lobe. Scale bar: 0.5 mm. **Figure S9.** Male genitalia. **A–C***Merodon pruni***D–F***M. hypochrysos* Hurkmans, 1993 **G–I***M. serrulatus***J–L***M. hirtus*. **A–B, D–E, G–H, J–K** epandrium **C, F, I, L** hypandrium. **A, C, D, F, G, I, J, L** lateral view **B, E, H, K** ventral view. Abbreviations: pl-posterior surstyle lobe. **G** basolateral protrusion marked with arrow. Scale bar: 0.5 mm (**A–C**); 0.2 mm (**D–L**). **Figure S10.** Male genitalia. **A–C***Merodon auronitens***D–F***M. caudatus***G–I***M. clunipes***J–L***M. crassifemoris*. **A–B, D–E, G–H, J–K** epandrium **C, F, I, L** hypandrium. **A, C, D, F, G, I, J, L** lateral view **B, E, H, K** ventral view. Abbreviations: pl-posterior surstyle lobe, s-lateral sclerite of the aedeagus. **A** triangular basal extension marked with arrow **F** ventral processes of the hypandrium marked with arrow **G** apical hump marked with arrow. Scale bar: 0.2 mm (**A–C, G–L**); 0.5 mm (**D–F**). **Figure S11.** Male genitalia. **A–C***Merodon eumerusi***D–F***M. murinus***G–I***M. ottomanus*. **A–B, D–E, G–H** epandrium **C, F, I** hypandrium. **A, C, D, F, G, I** lateral view **B, E, H** ventral view. Abbreviations: al-anterior surstyle lobe, pl-posterior surstyle lobe. Scale bar: 0.2 mm. **Figure S12.** Male genitalia. **A–C***Merodon capensis***D–F***M. desuturinus***G–I***M. neolydicus***J–L***M. planifacies*. **A–B, D–E, G–H, J–K** epandrium **C, F, I, L** hypandrium. **A, C, D, F, G, I, J, L** lateral view **B, E, H, K** ventral view. Abbreviations: al-anterior surstyle lobe, s-lateral sclerite of the aedeagus. Scale bar: 0.2 mm. **Figure S13.** Male genitalia. **A–C***Merodon natans* (Fabricius, 1794) **D–F***M. segetum*. **A, B, D, E** epandrium **C, F** hypandrium. **A, C, D, F** lateral view **B, E** ventral view. Abbreviations: al-anterior surstyle lobe. Scale bar: 0.2 mm. https://binary.pensoft.net/file/531632This dataset is made available under the Open Database License (http://opendatacommons.org/licenses/odbl/1.0/). The Open Database License (ODbL) is a license agreement intended to allow users to freely share, modify, and use this Dataset while maintaining this same freedom for others, provided that the original source and author(s) are credited.Ante Vujić, Snežana Radenković, Laura Likov, Sanja Veselić

Supplementary material 3 Figures S1–S15: Figures of adults Data type Adobe PDF file Explanation note **Figure S1.** Body of male. **A, B***Merodon albifrons* Meigen, 1822 **C, D***M. constans* (Rossi, 1794). **A, C** dorsal view **B, D** lateral view. Scale bar: 1 mm. **Figure S2.** Body of male. **A, B***Merodon equestris***C, D***M. geniculatus*. **A, C** dorsal view **B, D** lateral view. Scale bar: 3 mm (**A, B**); 1 mm (**C, D**). **Figure S3.** Body of male. **A, B***Merodon ruficornis* Meigen, 1822 **C, D***M. rufus*. **A, C** dorsal view **B, D** lateral view. Scale bar: 1.5 mm. **Figure S4.** Body of male. **A, B***Merodon luteihumerus***C, D***M. mixtum*. **A, C** dorsal view **B, D** lateral view. Scale bar: 2 mm. **Figure S5.** Body of male, dorsal view. **A***Merodon aureus* Fabricius, 1805 **B***M. bessarabicus* Paramonov, 1924 **C***M. cinereus* (Fabricius, 1794) **D***M. chalybeus* Wiedemann, 1822. Scale bar: 2 mm (**A–C**); 1.5 mm (**D**). **Figure S6.** Body of male, dorsal view. **A***Merodon caerulescens* Loew, 1869 **B***M. dobrogensis***C***M. funestus***D***M. bombiformis*. Scale bar: 1.5 mm (**A, B**), 2 mm (**C, D**). **Figure S7.** Body of male, dorsal view. **A***Merodon spinitarsis***B***M. nanus* (Sack, 1931). Scale bar: 1 mm. **Figure S8.** Body of male, dorsal view. **A***Merodon aberrans***B***M. aurifer***C***M. avidus* (Rossi, 1790) **D***M. italicus*. Scale bar: 2 mm (**A–C**); 1.5 mm (**D**). **Figure S9.** Body of male. **A–B***Merodon clavipes* (Fabricius, 1781) **C***M. fulcratus***D***M. serrulatus*. **A, C, D** dorsal view **B** lateral view. Scale bar: 1 mm. **Figure S10.** Body of male. **A, B***Merodon pruni***C***M. nigritarsis* Rondani, 1845 **D***M. auronitens*. **A, C, D** dorsal view **B** lateral view. Scale bar: 2 mm (**A–C**); 1.5 mm (**D**). **Figure S11.** Body of male. **A, B***Merodon tarsatus***C, D***M. caudatus*. **A, C** dorsal view **B, D** lateral view. Scale bar: 1.5 mm. **Figure S12.** Body of male. **A, B***Merodon clunipes***C, D***M. crassifemoris*. **A, C** dorsal view **B, D** lateral view. Scale bar: 2 mm. **Figure S13.** Body of male, dorsal view. **A***Merodon eumerusi***B** M. *hirtus***C***M. murinus***D***M. ottomanus*. Scale bar: 1.5 mm (**A, C**); 2 mm (**B, D**). **Figure S14.** Body of male, dorsal view. **A***Merodon melanocerus***B***M. desuturinus***C***M. cuthbertsoni*. Scale bar: 2 mm (**A–B**). **Figure S15.** Body of male, dorsal view. **A***Merodon natans* (Fabricius, 1794) **B***M. segetum*. Scale bar: 1.5 mm (**A**); 2 mm (**B**). https://binary.pensoft.net/file/531633This dataset is made available under the Open Database License (http://opendatacommons.org/licenses/odbl/1.0/). The Open Database License (ODbL) is a license agreement intended to allow users to freely share, modify, and use this Dataset while maintaining this same freedom for others, provided that the original source and author(s) are credited.Ante Vujić, Snežana Radenković, Laura Likov, Sanja Veselić

Supplementary material 4 List of *Merodon* species grouped by *lineage* and species group Data type species data https://binary.pensoft.net/file/531634This dataset is made available under the Open Database License (http://opendatacommons.org/licenses/odbl/1.0/). The Open Database License (ODbL) is a license agreement intended to allow users to freely share, modify, and use this Dataset while maintaining this same freedom for others, provided that the original source and author(s) are credited.Ante Vujić, Snežana Radenković, Laura Likov, Sanja Veselić

Supplementary material 5 Table S1. Classification of the genus *Merodon* in lineages and species groups, including an actual number of species within each Data type species data https://binary.pensoft.net/file/531635This dataset is made available under the Open Database License (http://opendatacommons.org/licenses/odbl/1.0/). The Open Database License (ODbL) is a license agreement intended to allow users to freely share, modify, and use this Dataset while maintaining this same freedom for others, provided that the original source and author(s) are credited.Ante Vujić, Snežana Radenković, Laura Likov, Sanja Veselić
