# Supplementary material for: Taxonomic complexity in the genus Merodon Meigen, 1803 (Diptera, Syrphidae)
Source: Zookeys. 2021 Apr 14;1031:85–124. doi: 10.3897/zookeys.1031.62125 (PMC8060246; doi:10.3897/zookeys.1031.62125)
Supplement: Supplementary material 1 — Figures S1–S37: Figures of morphological characters [file zookeys-1031-085-s001.pdf]

Supplementary file S1: Figures of morphological characters

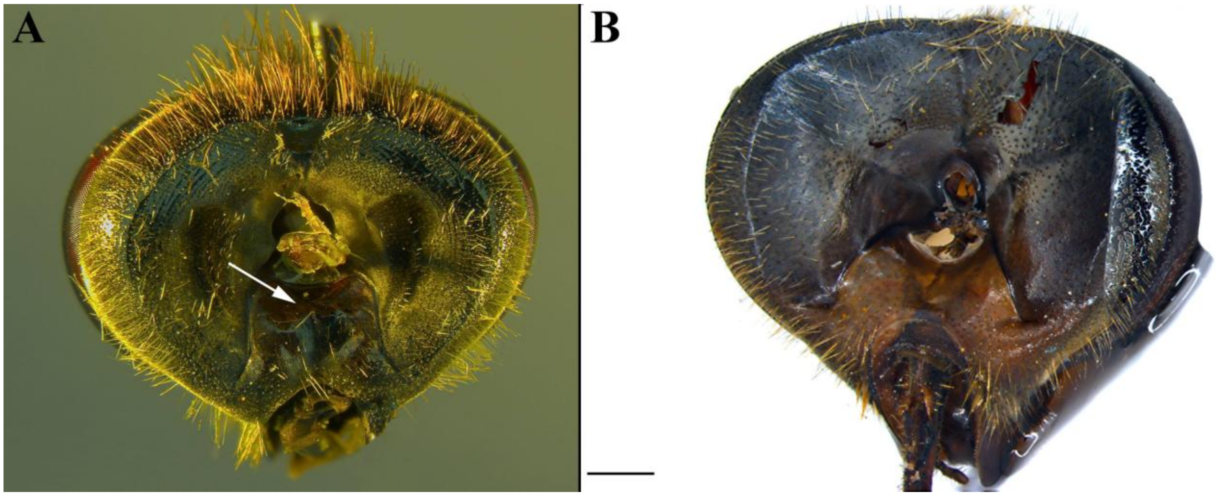

Figure 1. Head of male, posterior view. **A** *Platynochaetus setosus* (Fabricius, 1794), hypostomal bridge marked with arrow **B** *Sericomyia silentis* (Harris, 1776). Scale bar: 0.5 mm.

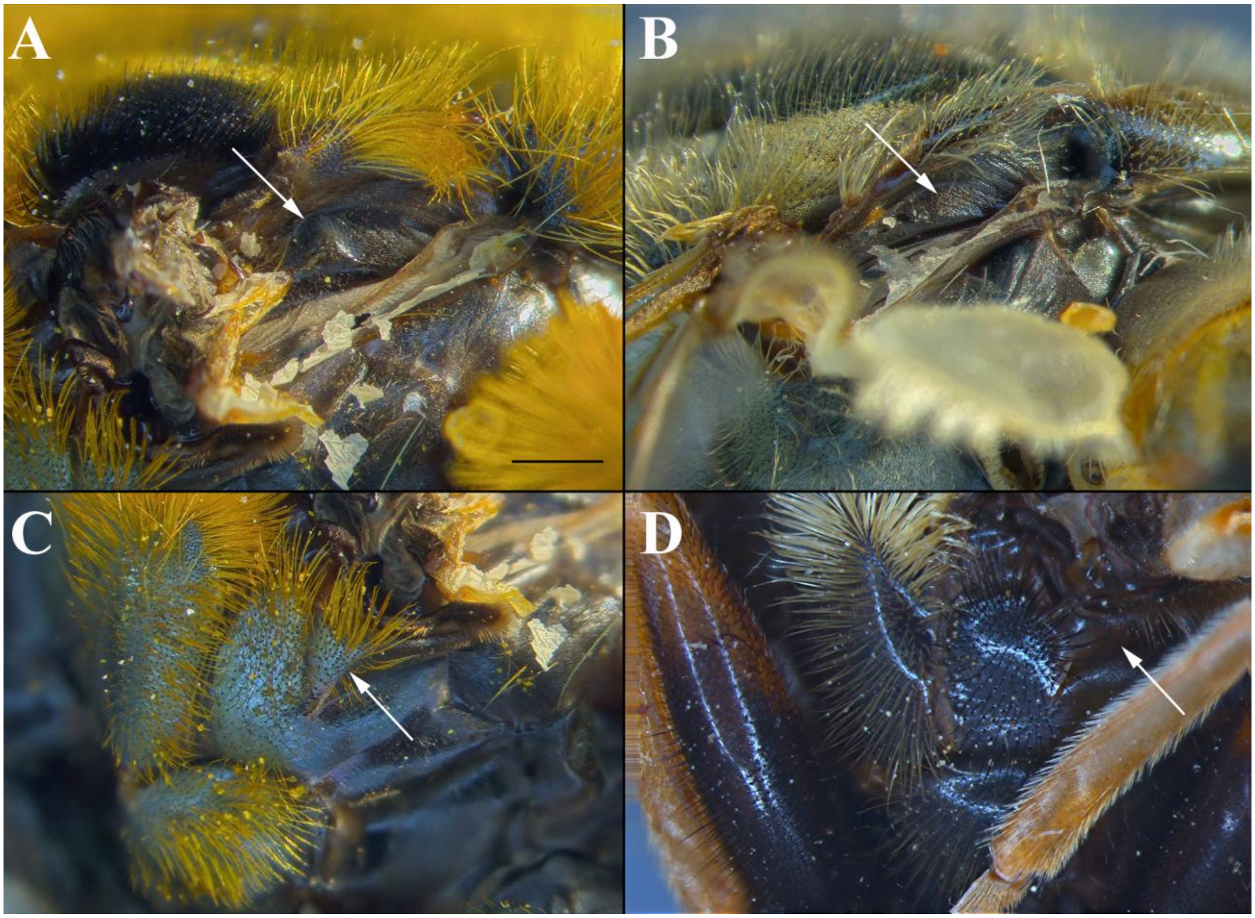

Figure 2. Parts of thorax of male, lateral view. **A** *Merodon megavidus* Vujić & Radenković, 2016, tubercle **B** *Eristalinus megacephalus* (Rossi, 1794), tubercle **C** *Merodon megavidus*, anepimeron **D** *Sericomyia silentis*, anepimeron. **A–B** tubercle marked with arrow **C–D** dorsomedian part of anepimeron marked with arrow. Scale bar: 0.5 (**A–C**); 1 mm (**D**).

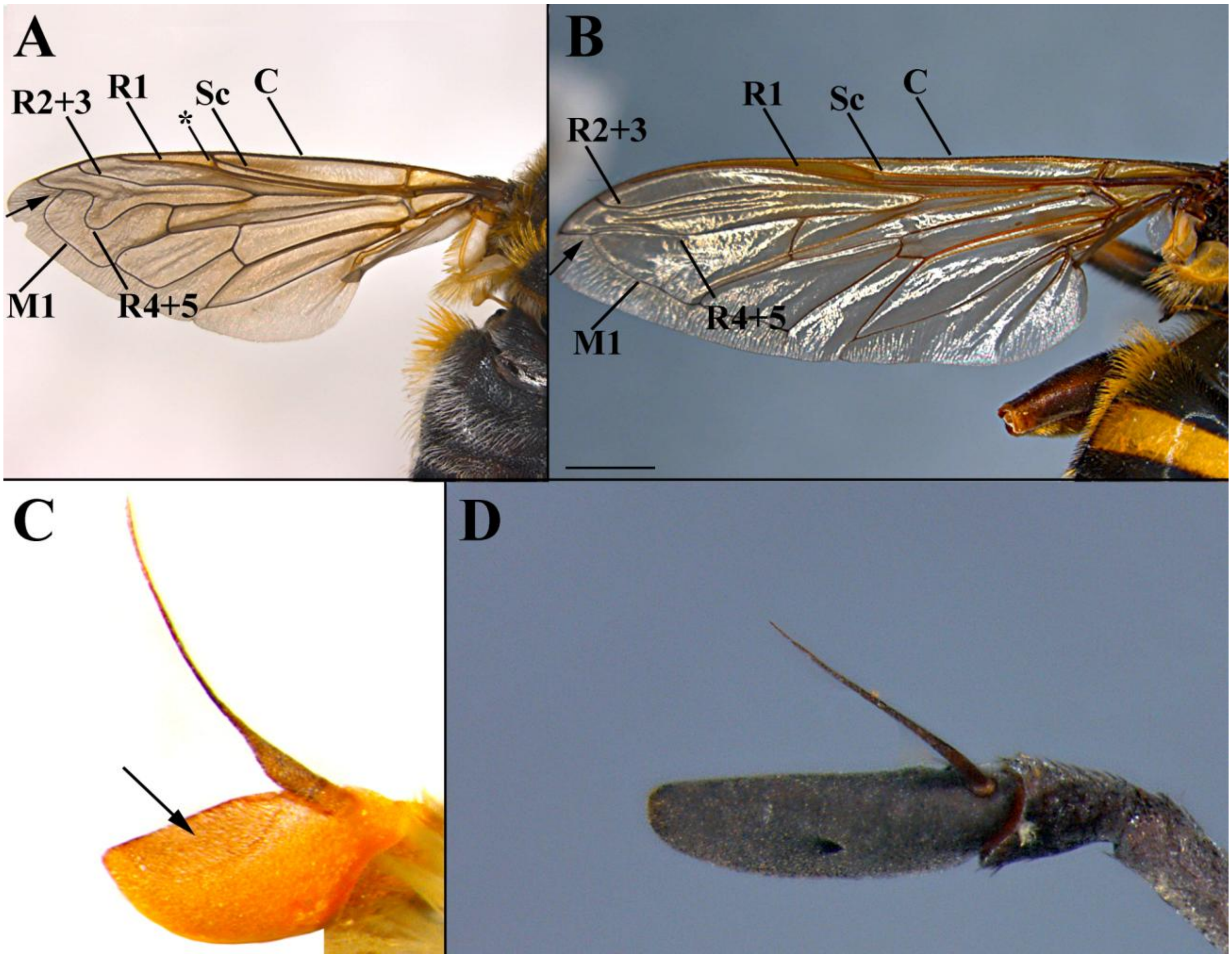

Figure 3. **A**–**B** Wing of male, dorsal view **C**–**D** Antenna of male, lateral view. **A** *Merodon* aff. *nasicus*, short vein marked with asterisk **B** *Sericomyia silentis* **C** *Merodon ottomanus*, fossette marked with arrow **D** *Microdon analis*. **A**–**B** outer angle between R4+5 and M1 marked with arrow. Scale bar: 0.5 mm (**A**–**B, D**); 0.2 mm (C).

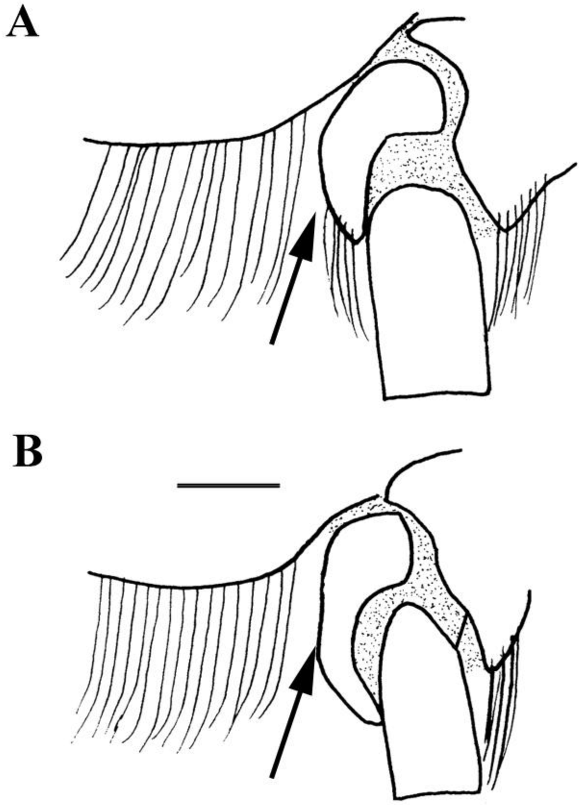

Figure 4. Metacoxa of male. **A** *Merodon albifrons* Meigen, 1822, lateral view **B** *M. nigritarsis* Rondani, 1845, lateral view. **A–B** pile marked with arrow. Scale bar: 0.25 mm.

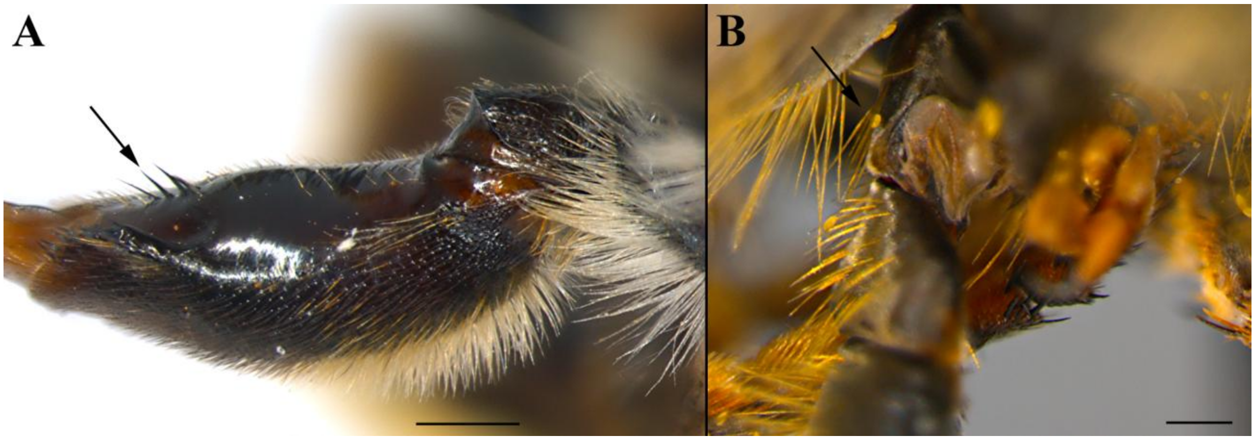

Figure 5. A Metafemur of *Merodon eumerusi*, male, lateroventral view, row of spinae marked with arrow **B** Mesocoxa of *M. segetum*, male, lateral view, pile marked with arrow. Scale bar: 1 mm.

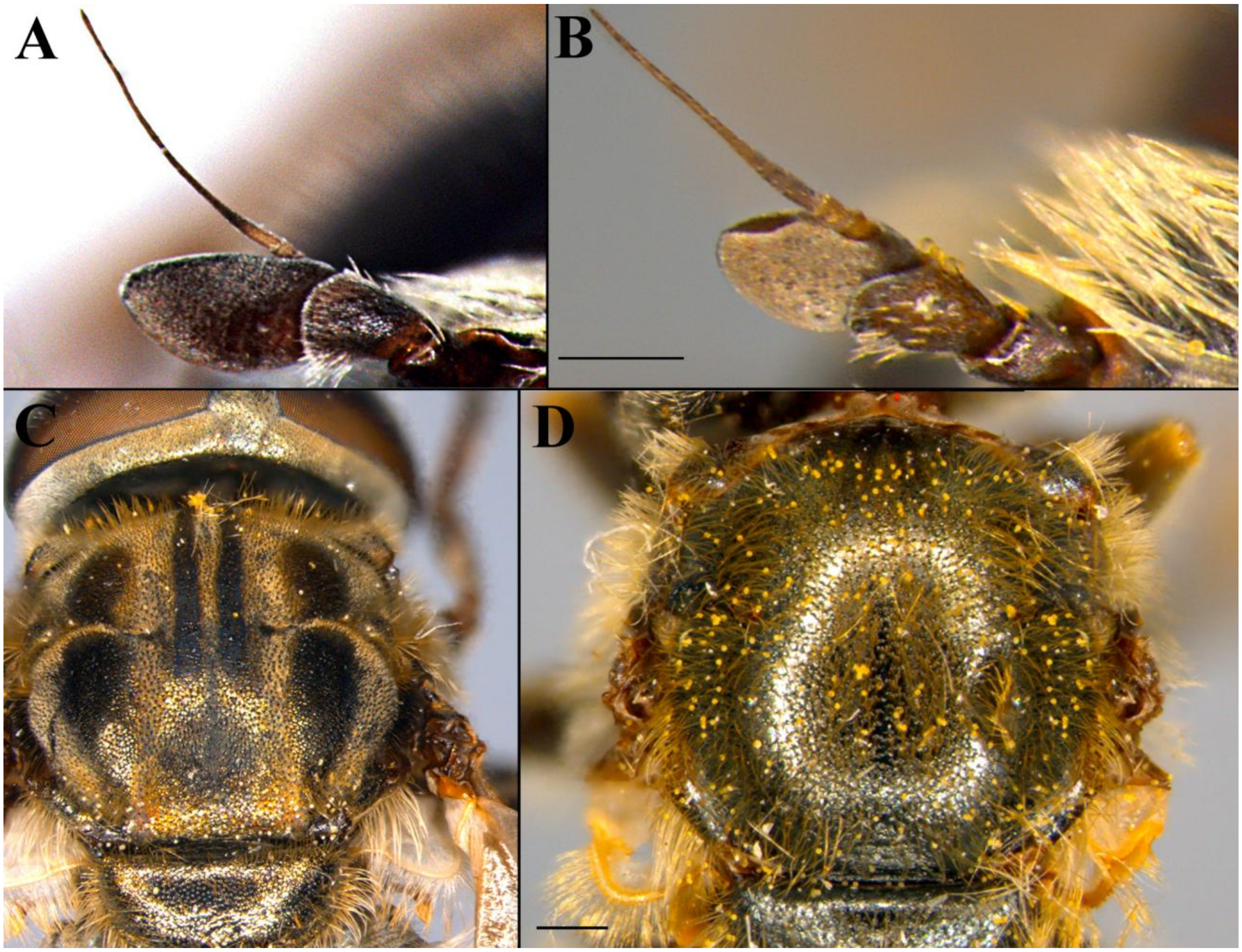

Figure 6. A–B Antenna of male, lateral view **C–D** Thorax of male, dorsal view. **A, C** *Merodon natans* (Fabricius, 1794) **B, D** *M. desuturinus*. Scale bar: 1 mm.

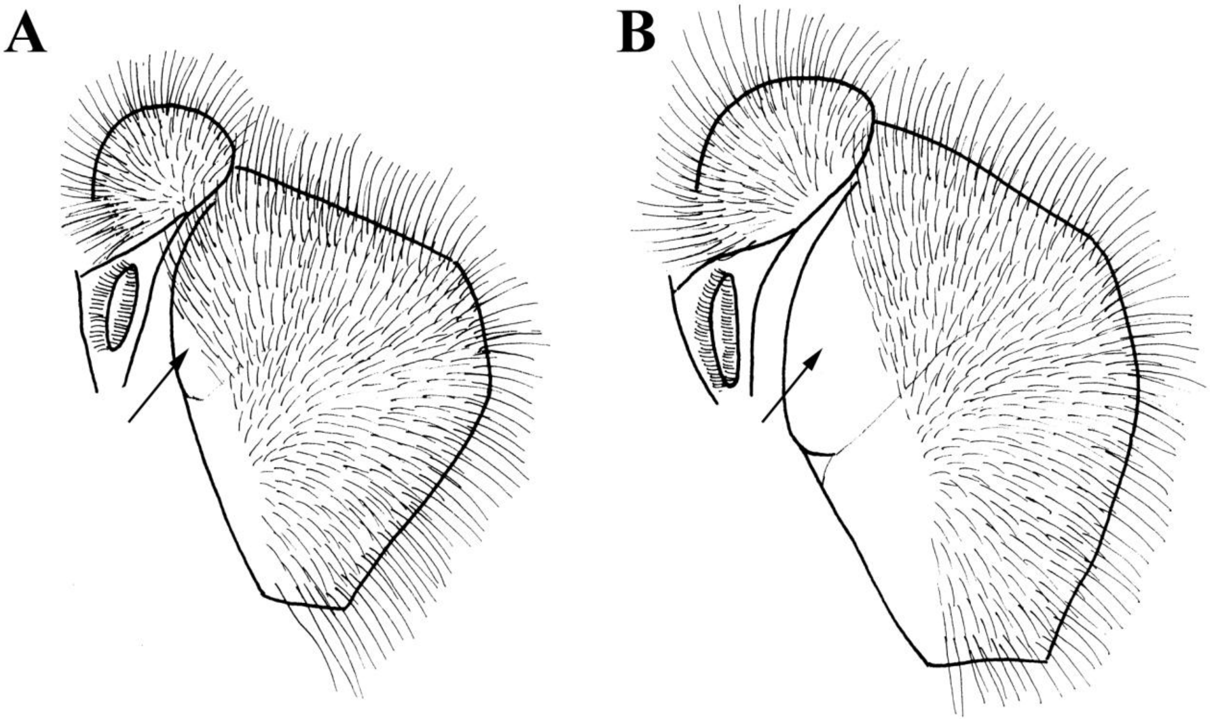

Figure 7. Thorax (anepisternum) of male, lateral view. **A** *Merodon geniculatus* **B** *M. legionensis* Marcos-García, Vujić & Mengual, 2007. **A–B** pile marked with arrow.

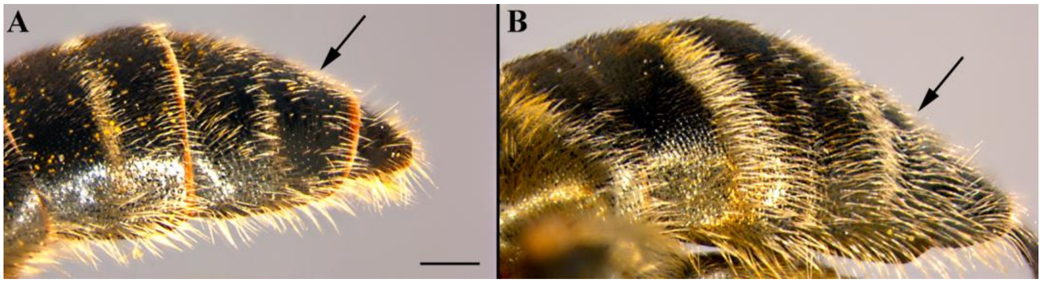

Figure 8. Abdomen of female, lateral view. **A** *Merodon desuturinus* **B** *M. aureus* Fabricius, 1805. **A–B** pile marked with arrow. Scale bar: 1 mm.

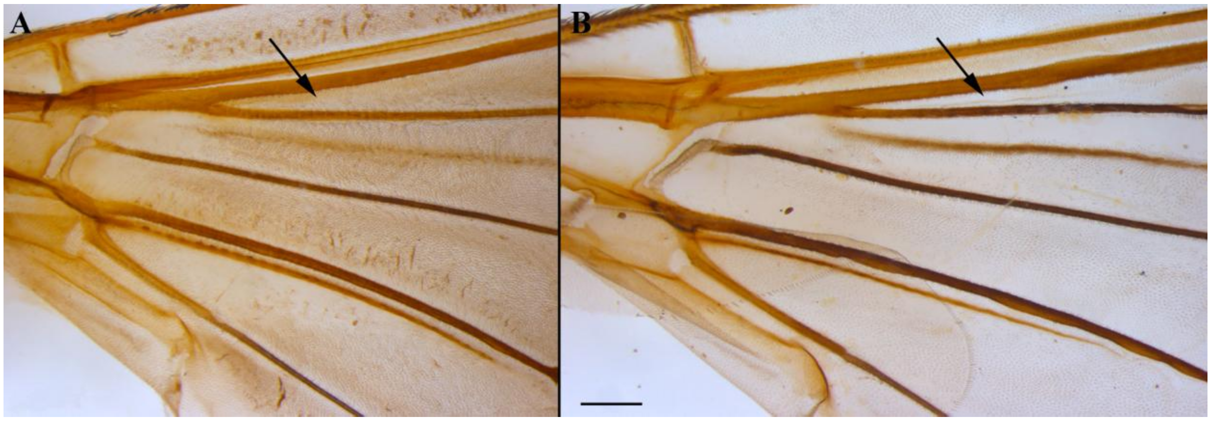

Figure 9. Part of wing of male, dorsal view. **A** *Merodon desuturinus* **B** *M. albifrons*. **A–B** microtrichia marked with arrow. Scale bar: 1 mm.

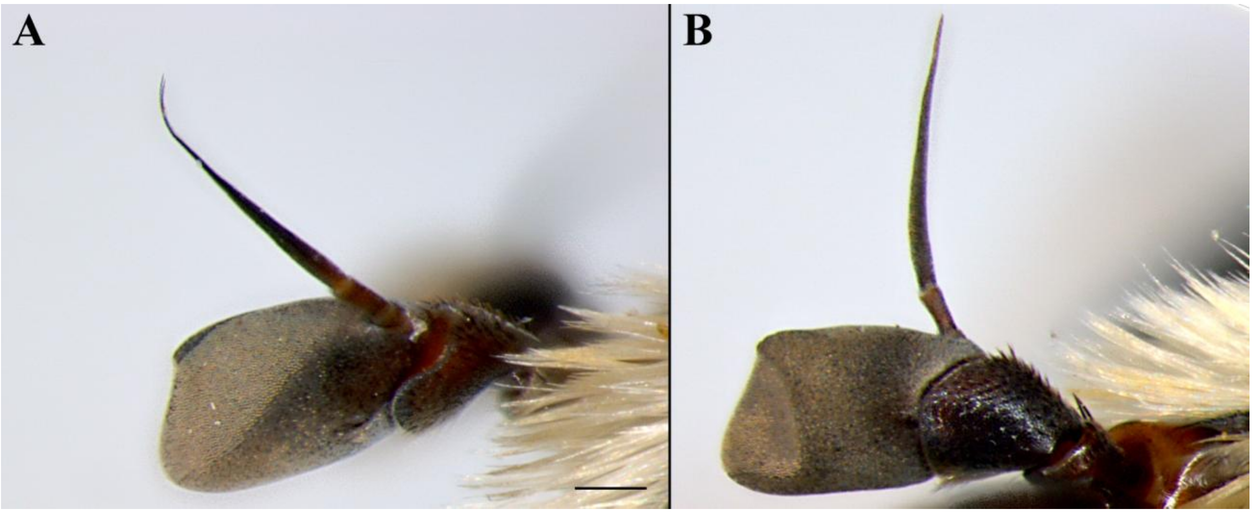

Figure 10. Antenna of *Merodon eumerusi*, male, lateral view. **A** outer side **B** inner side. Scale bar: 1 mm.

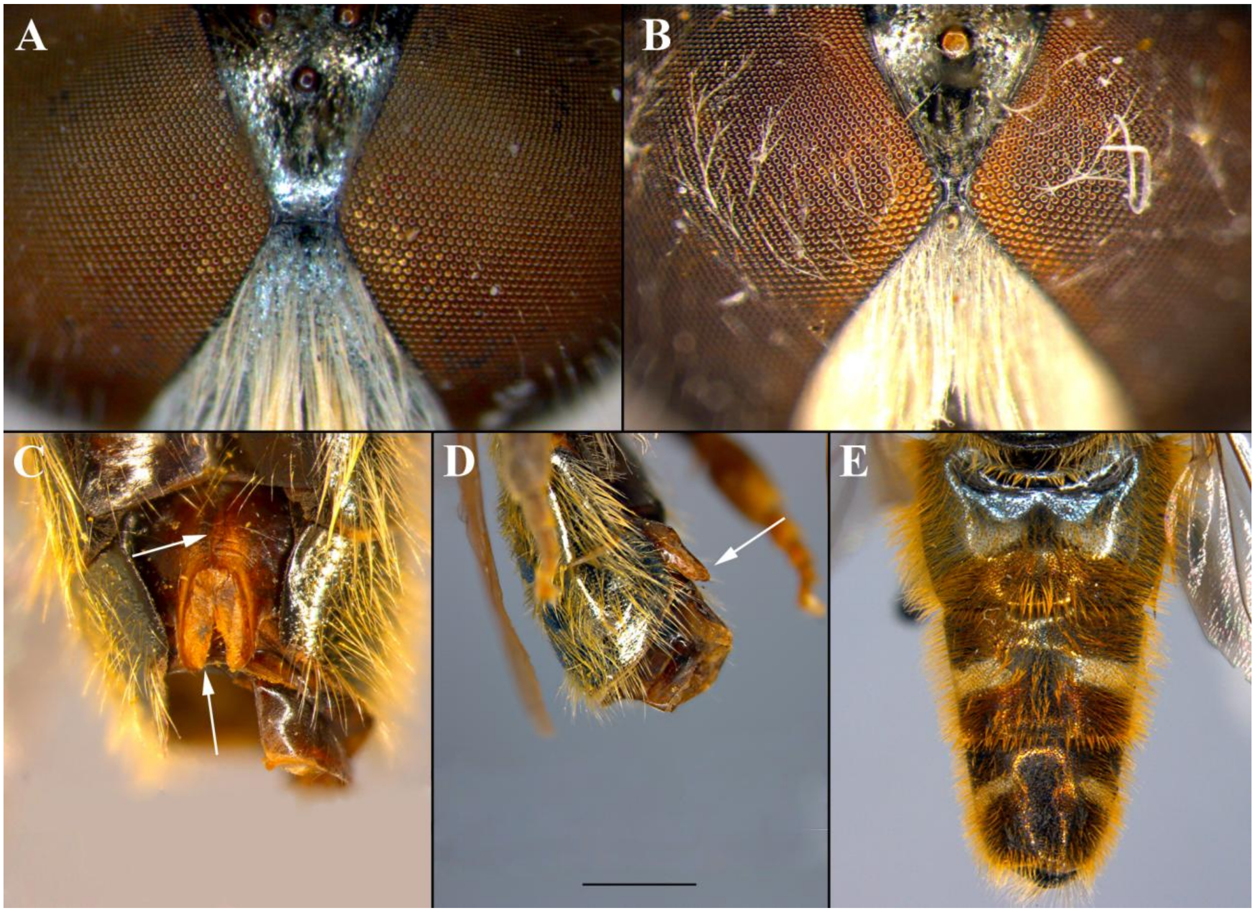

Figure 11. **A–B** Eye contiguity of male, anterior view **C–D** Sternum 4 of male, dorsal (**C**) and lateral (**D**) view **E** Abdomen of male, dorsal view. **A** *Merodon fulcratus* **B** *M. hirtus* **C-D** *M. tarsatus* **E** *M. aberrans*. **C–D** membranous structure and laminate extension marked with arrow. Scale bar: 1 mm (**A–B**); 2 mm (**C–E**).

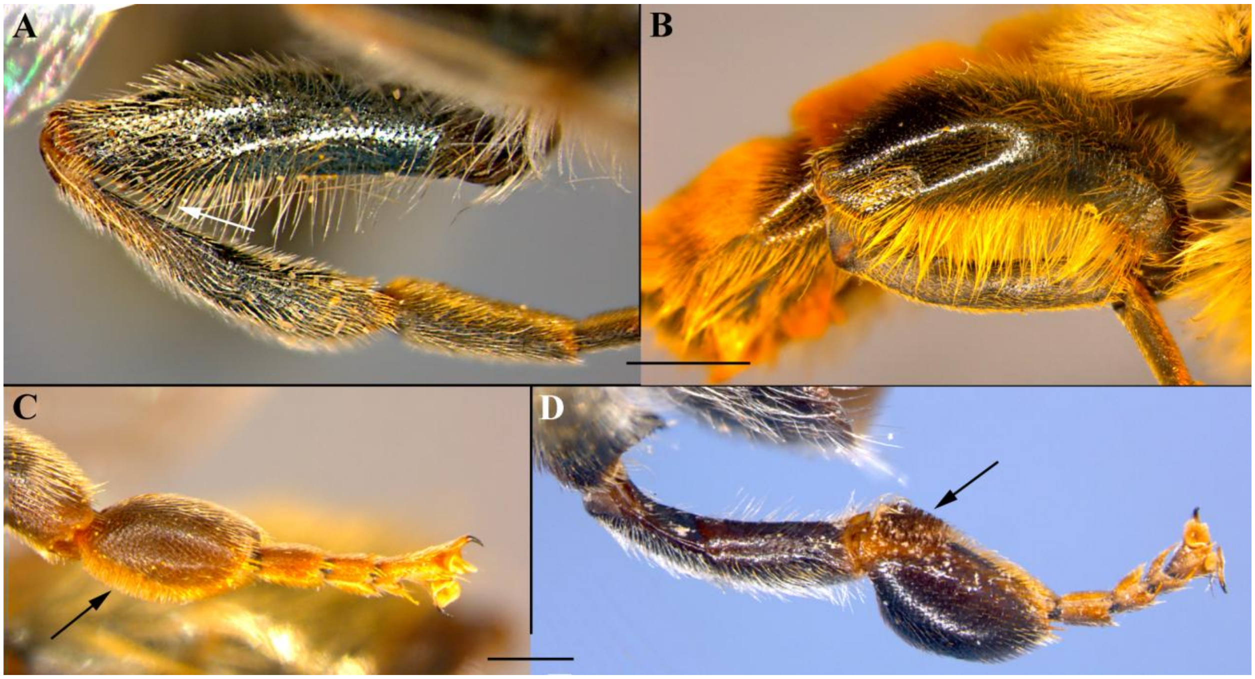

Figure 12. Parts of male metaleg, lateral view. **A** *Merodon fulcratus*, metaleg **B** *M. clavipes* (Fabricius, 1781), metafemur and metatibia **C** *M. tarsatus*, metatarsus **D** *M. oidipous* Hurkmans, 1993, metatarsus. **C–D** setae marked with arrrow. Scale bar: 2 mm (**A–B**); 0.5 mm (**C–D**).

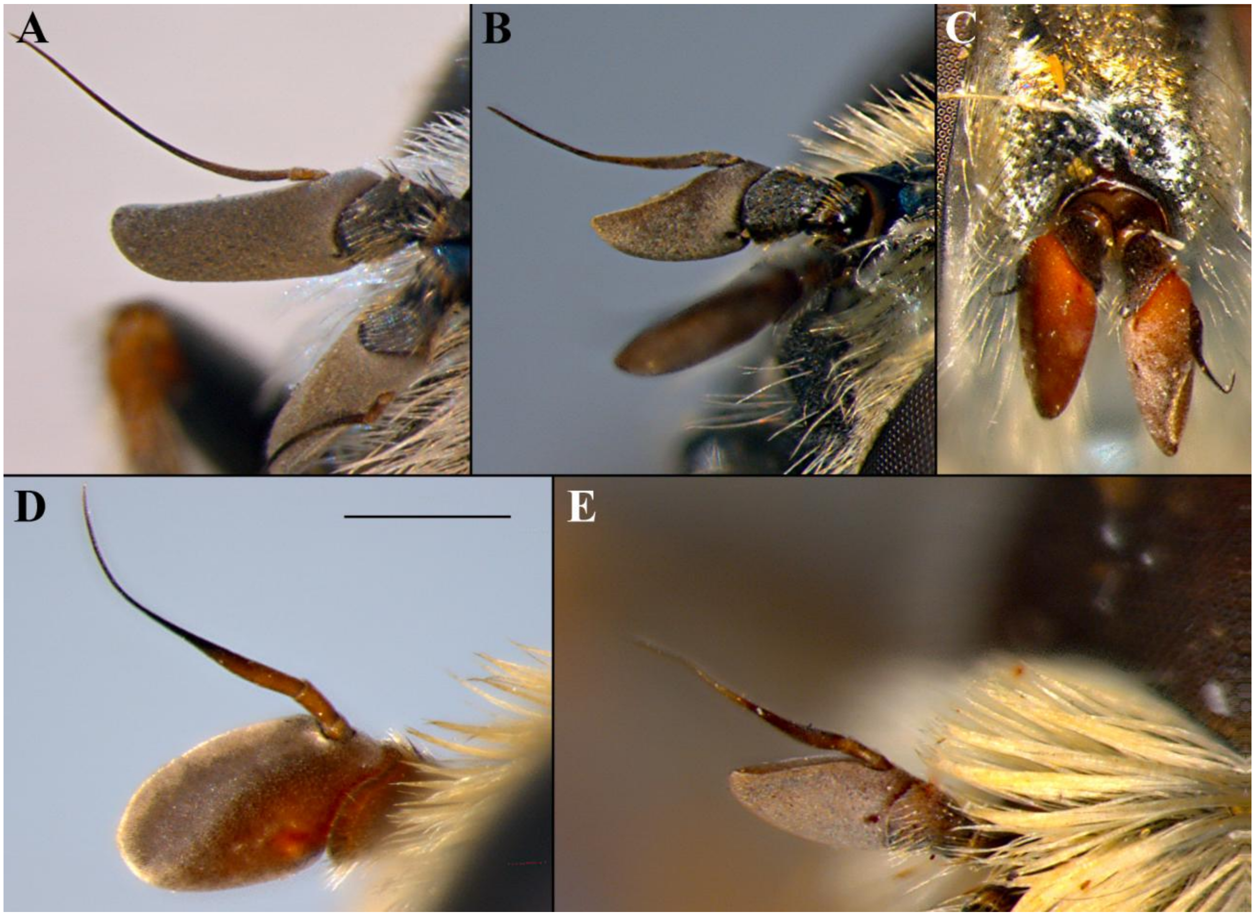

Figure 13. Male antenna, lateral view. **A** *Merodon italicus* **B** *M. serrulatus* **C** *M. ottomanus* **D** *M. clunipes* **E** *M. auronitens*. Scale bar: 2 mm.

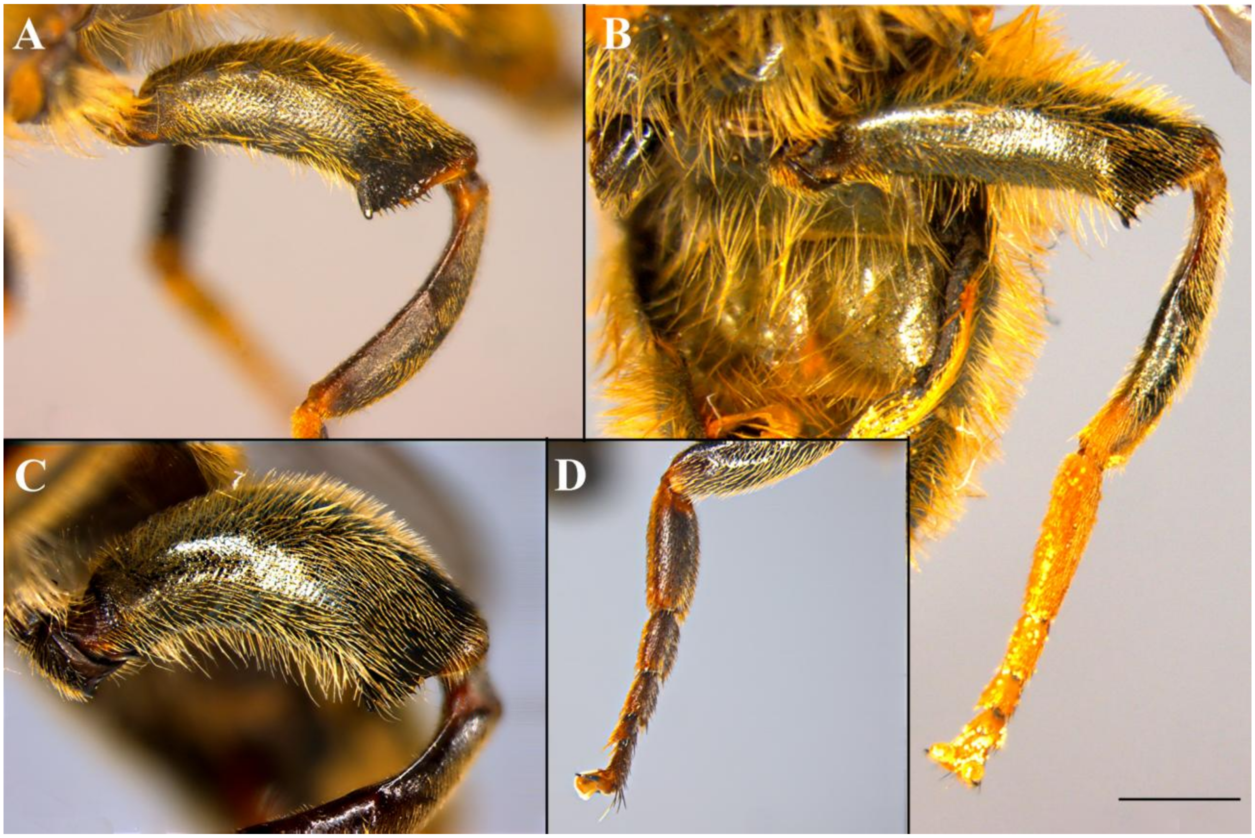

Figure 14. Parts of male metaleg, lateral view. **A** *Merodon serrulatus*, metafemur and metatibia **B** *M. ottomanus*, metaleg **C** *M. clunipes*, metafemur **D** *M. clunipes*, metatarsus. Scale bar: 2 mm.

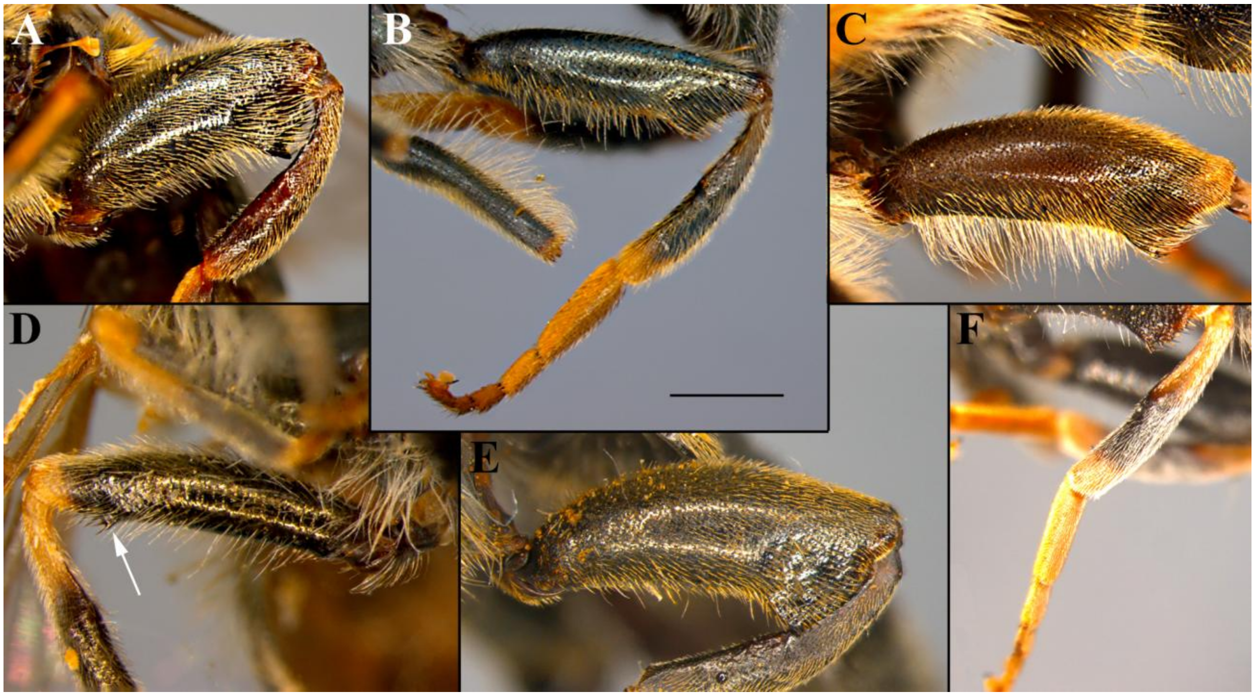

Figure 15. Parts of female metaleg, lateral view. **A** *Merodon clunipes*, metafemur and metatibia **B** *M. ottomanus*, metaleg **C** *M. clavipes*, metafemur **D** *M. fulcratus*, metafemur **E** *M. serrulatus*, metafemur **F** *M. murinus*, metatibia and metatarsus. **D** triangular lamina marked with arrow. Scale bar: 2 mm.

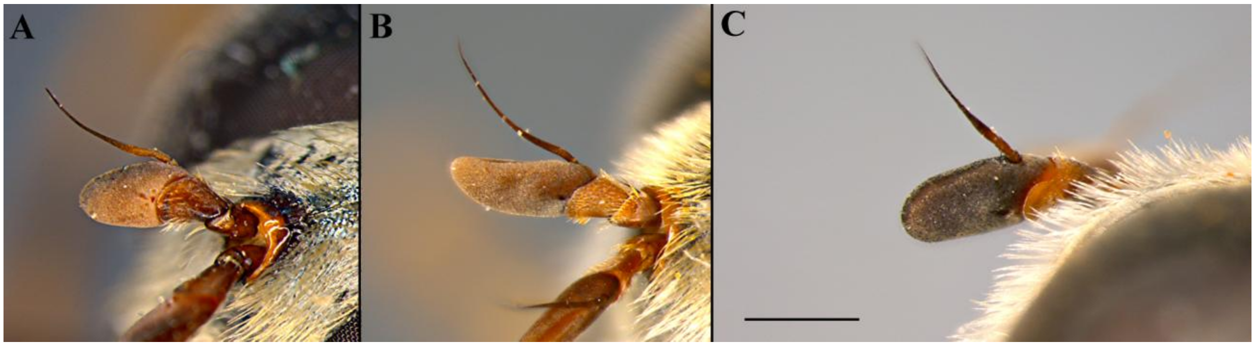

Figure 16. Female antenna, lateral view. **A** *Merodon clunipes* **B** *M. clavipes* **C** *M. murinus*. Scale bar: 2 mm.

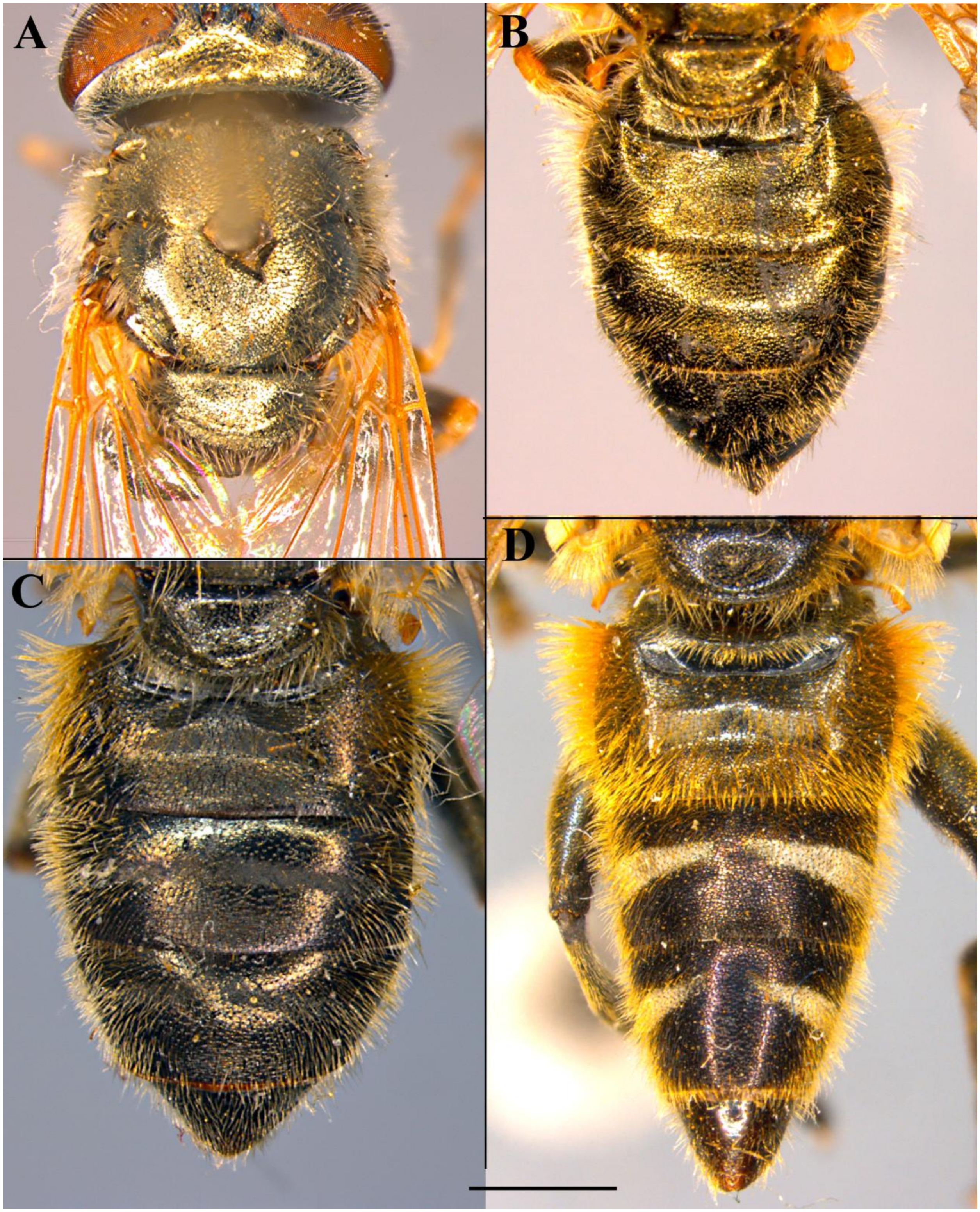

Figure 17. A Female thorax, dorsal view **B–D** Abdomen of female, dorsal view. **A–B** *Merodon fulcratus* **C** *M. ottomanus* **D** *M. aberrans*. Scale bar: 2 mm.

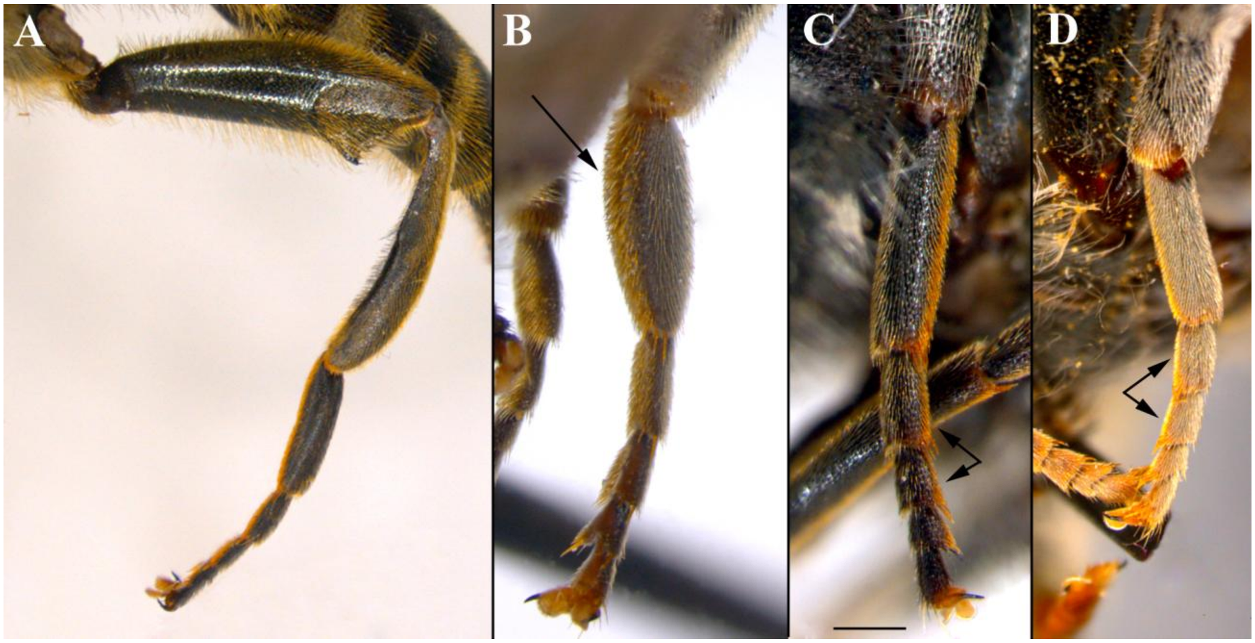

Figure 18. Parts of female metaleg, lateral view. **A** *Merodon aberrans*, metaleg **B** *M. tarsatus*, metatarsus **C** *M. hirtus*, metatarsus **D** *M. auronitens*, metatarsus. **B** setae marked with arrow **C-D** tarsomerae marked with arrow. Scale bar: 1 mm (**A**); 2 mm (**B**–**D**).

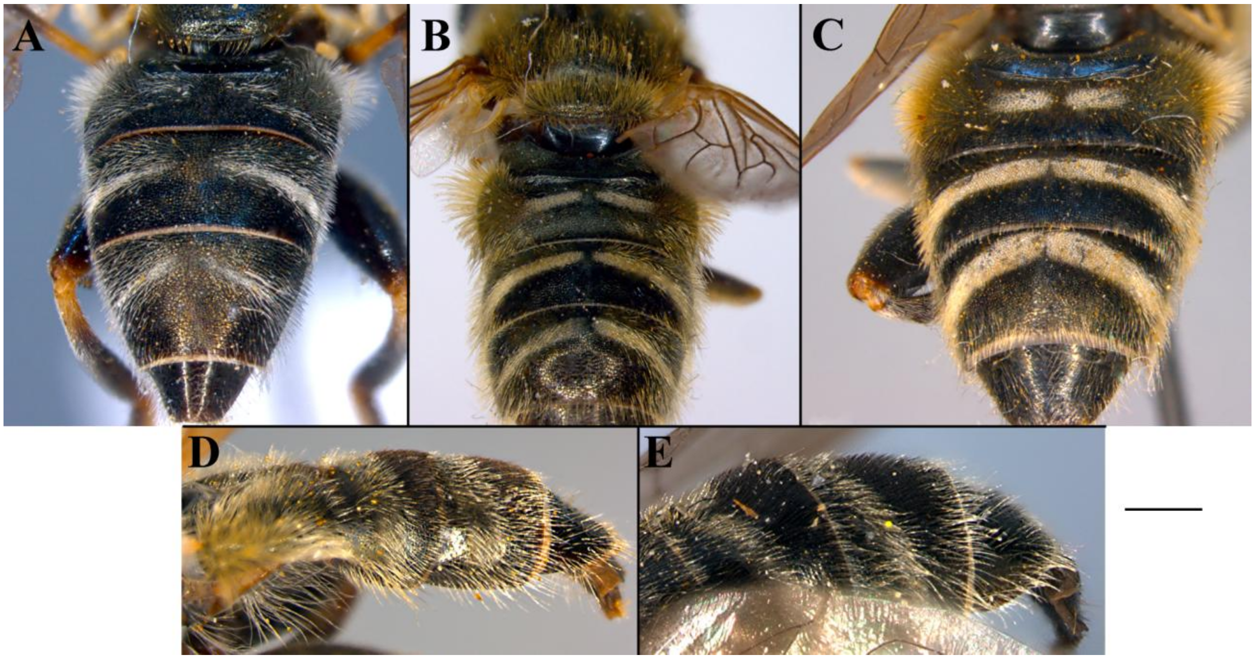

Figure 19. Abdomen of female. **A** *Merodon oidipous*, dorsal view **B** *M. tarsatus*, dorsal view **C** *M. auronitens*, dorsal view **D** *M. auronitens*, lateral view **E** *M. hirtus*, lateral view. Scale bar: 2 mm.

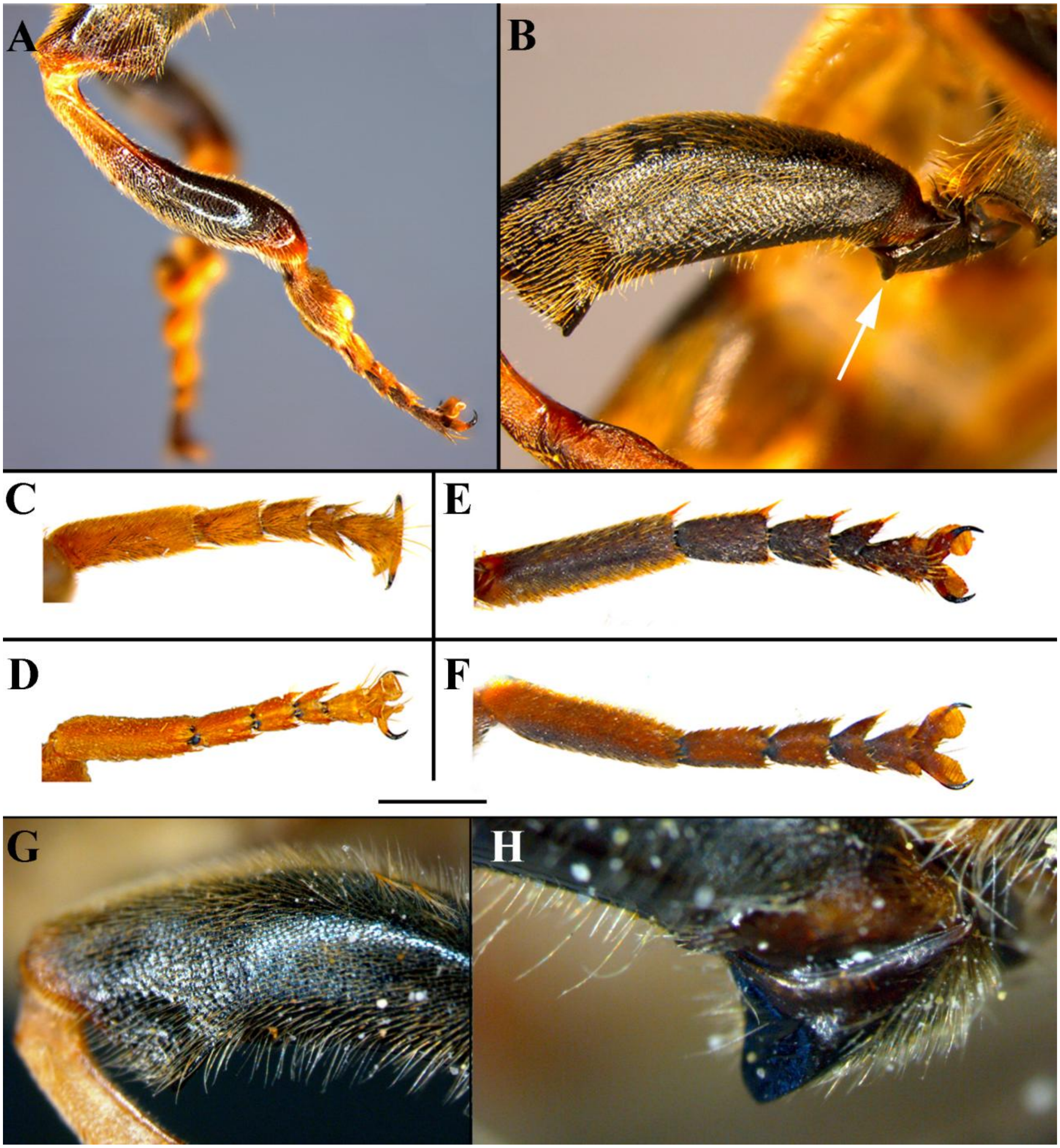

Figure 20. Parts of male metaleg. **A** *Merodon caudatus*, metatibia and metatarsus, lateral view **B** *M. aurifer*, metatrochanter and metafemur, lateral view **C** *M. avidus* (Rossi, 1790), metatarsus, dorsal view **D** *M. avidus*, metatarsus, ventral view **E** *M. nigritarsis*, metatarsus, dorsal view **F** *M. nigritarsis*, metatarsus, ventral view **G** *M. pruni*, part of metafemur, lateral view **H** *M. pruni*, metatrochanter, lateral view. **B** calcar marked with arrow. Scale bar: 0.5 mm (**A**–**B**); 1mm (**C**–**G**); 0.25 mm (**H**).

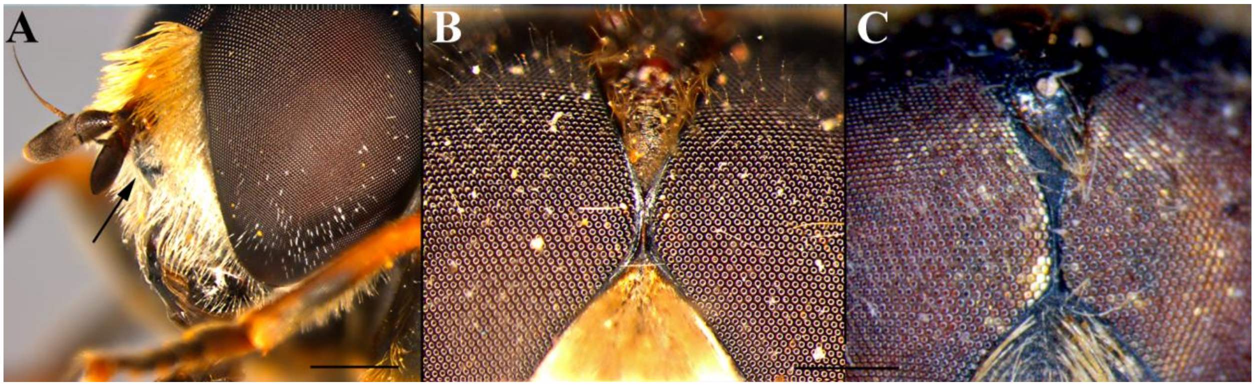

Figure 21. **A** *Merodon crassifemoris*, male, head, lateral view **B** *M. murinus*, male, eye contiguity, anterior view **C** *M. nigritarsis*, male, eye contiguity, anterior view. **A** bulge marked with arrow. Scale bar: 1 mm.

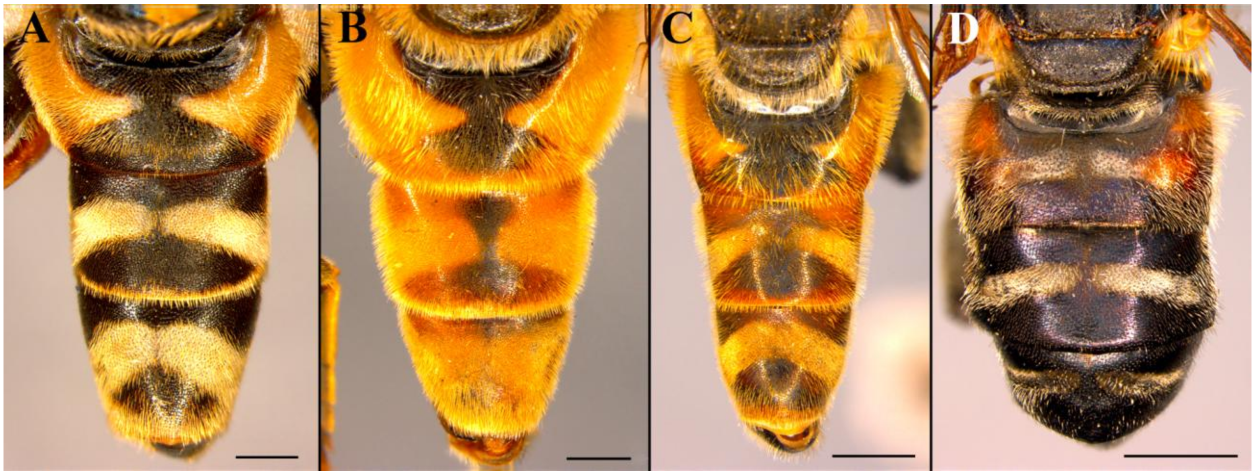

Figure 22. Abdomen, dorsal view. **A** *Merodon aurifer* **B** *M. pruni* **C** *M. avidus* **D** *M. serrulatus*. **A–C** male **D** female. Scale bar: 2 mm.

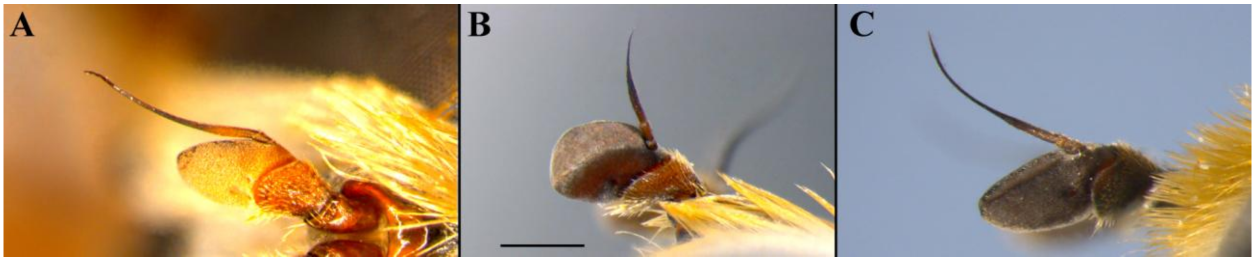

Figure 23. Male antenna, lateral view. **A** *Merodon aurifer* **B** *M. pruni* **C** *M. nigritarsis*. Scale bar: 1 mm (**A, C**); 0.5 mm (**B**).

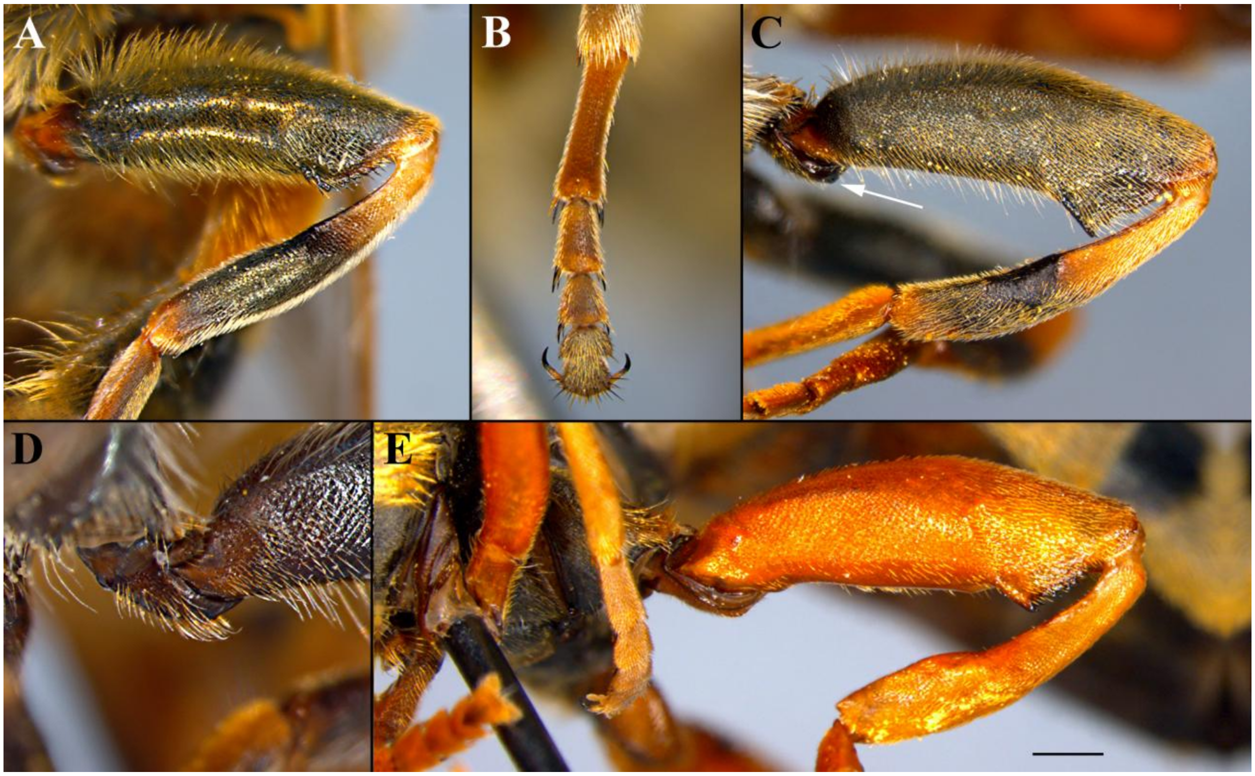

Figure 24. Parts of metaleg of female. **A** *Merodon caudatus*, metafemur and metatibia, lateral view **B** *M. caudatus*, metatarsus, dorsal view **C** *M. pruni*, metatrochanter, metafemur and metatibia **D** *M. crassifemoris*, metatrochanter, lateral view **E** *M. aurifer*, metafemur and metatibia. **C** metatrochanter marked with arrow. Scale bar: 1 mm (**A, C, E**); 0.5 mm (**B**); 0.75 mm (**D**).

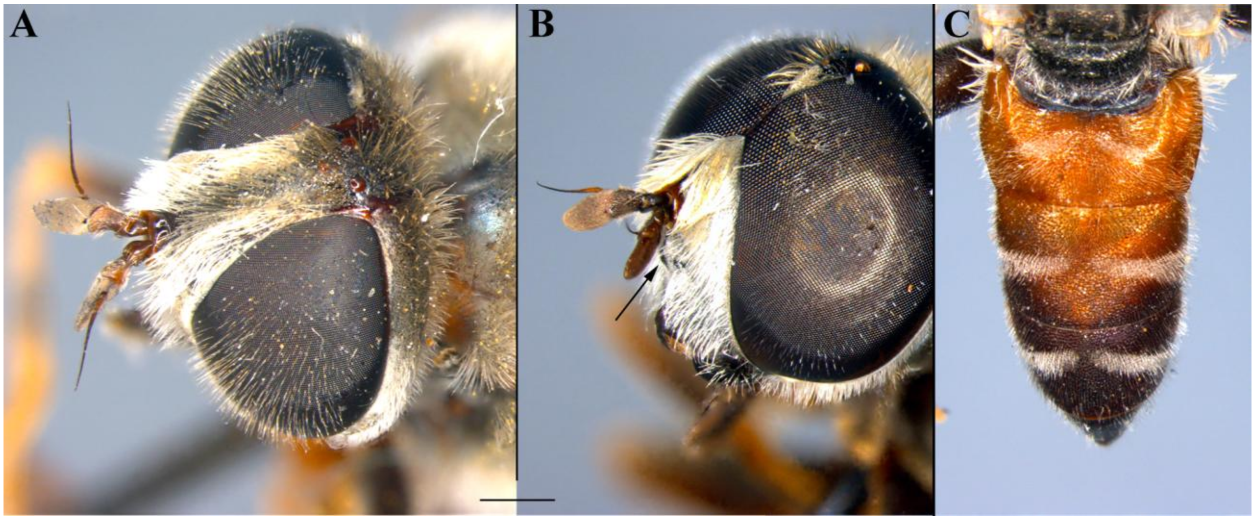

Figure 25. A *Merodon murinus*, female, head, dorsal view **B** *M. crassifemoris*, female, head, dorsolateral view **C** *M. italicus*, female, abdomen, dorsal view. **B** bulge marked with arrow. Scale bar: 1 mm (**A–B**); 2 mm (**C**).

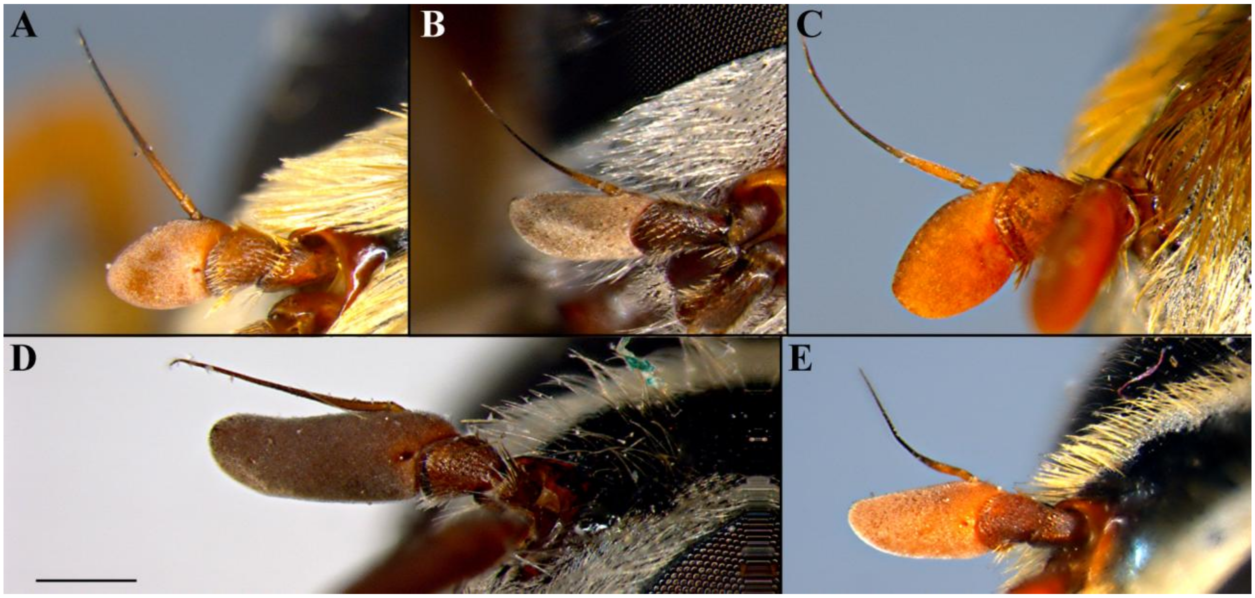

Figure 26. Antenna of female, lateral view. **A** *Merodon pruni* **B** *M. crassifemoris* **C** *M. aurifer* **D** *M. italicus* **E** *M. avidus*. Scale bar: 0.5 mm (**A–C, E**); 1 mm (**D**).

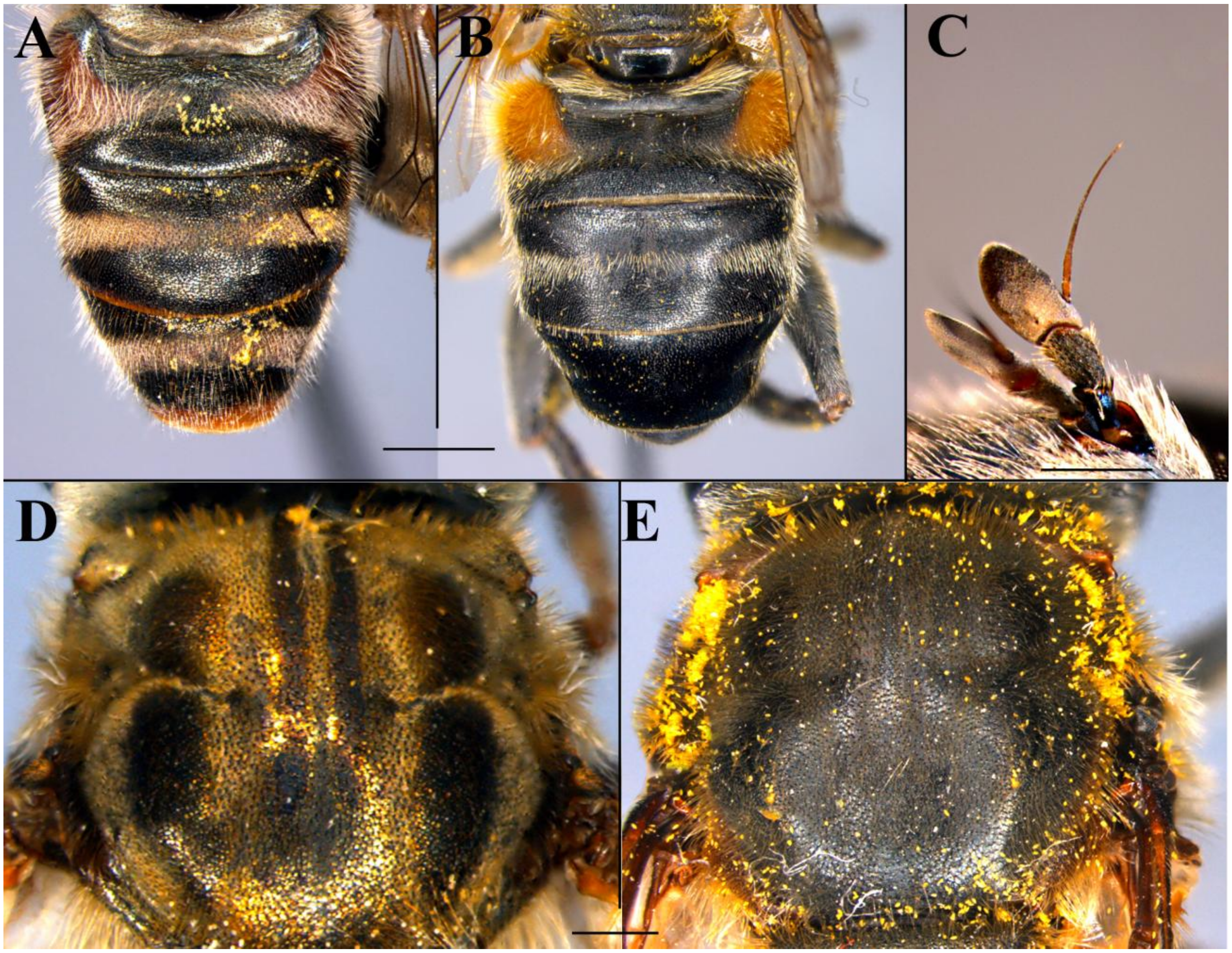

Figure 27. A *Merodon natans*, male, abdomen, dorsal view **B** *M. segetum*, male, abdomen, dorsal view **C** *M. natans*, male, antenna, lateral view **D** *M. natans*, male, thorax, dorsal view **E** *M. segetum*, male, thorax, dorsal view. Scale bar: 2 mm (**A–C**); 1 mm (**D**); 0.5 mm (**E**).

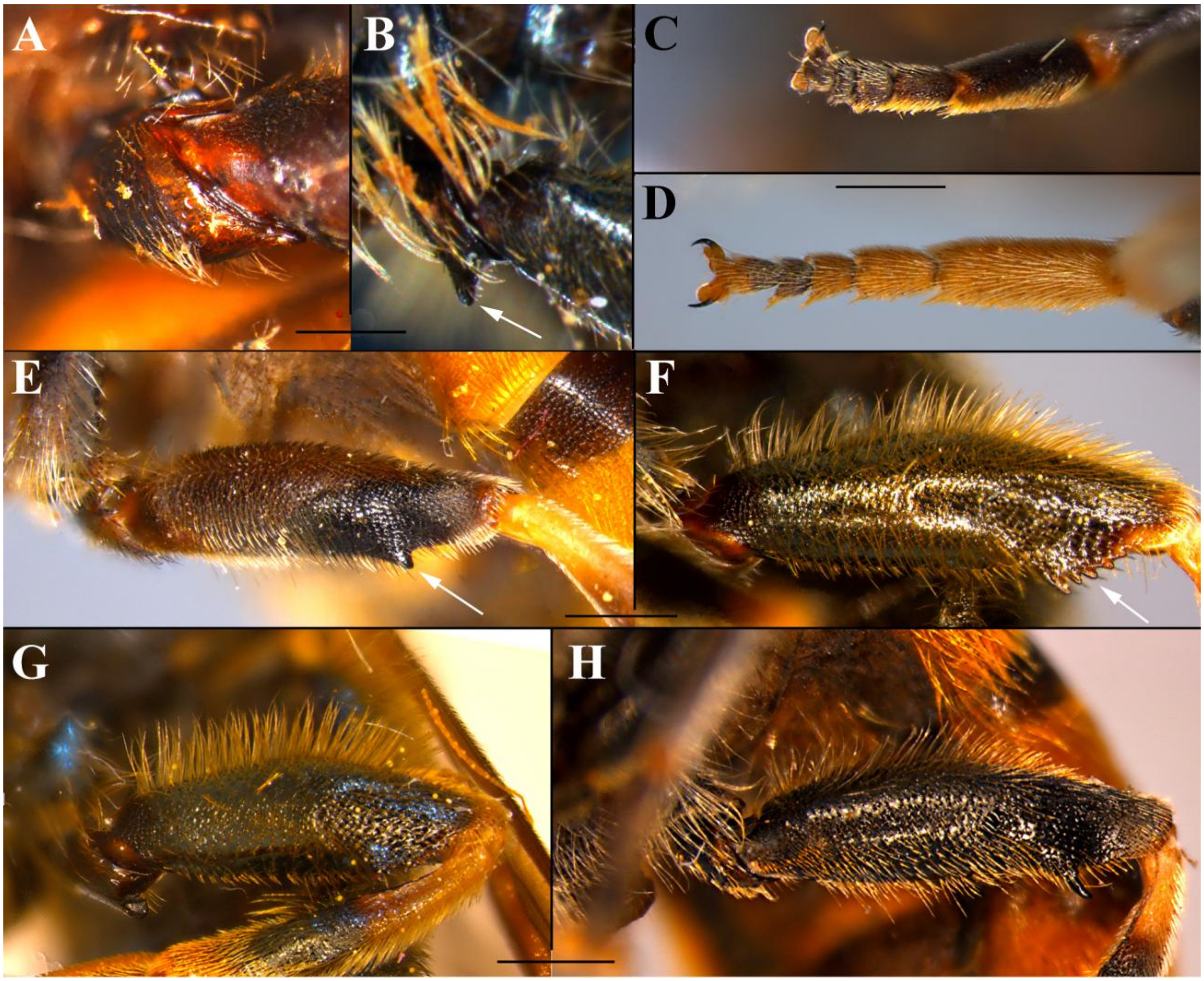

Figure 28. Parts of metaleg. **A** *Merodon bombiformis*, male, metatrochanter, lateral view **B** *M. aureus*, male, metatrochanter, lateral view **C** *M. spinitarsis*, female, metatarsus, dorsal view **D** *M. nanus* (Sack, 1931), female, metatarsus, dorsal view **E** *M. bombiformis*, female, metafemur, lateral view **F** *M. funestus*, female, metafemur, lateral view **G** *M. funestus*, male, metafemur and metatrochanter, lateral view **H** *M. bombiformis*, male, metafemur and metatrochanter, lateral view. **B** calcar on the metatrochanter marked with arrow **E** triangular lamina marked with arrow. Scale bar: 2 mm.

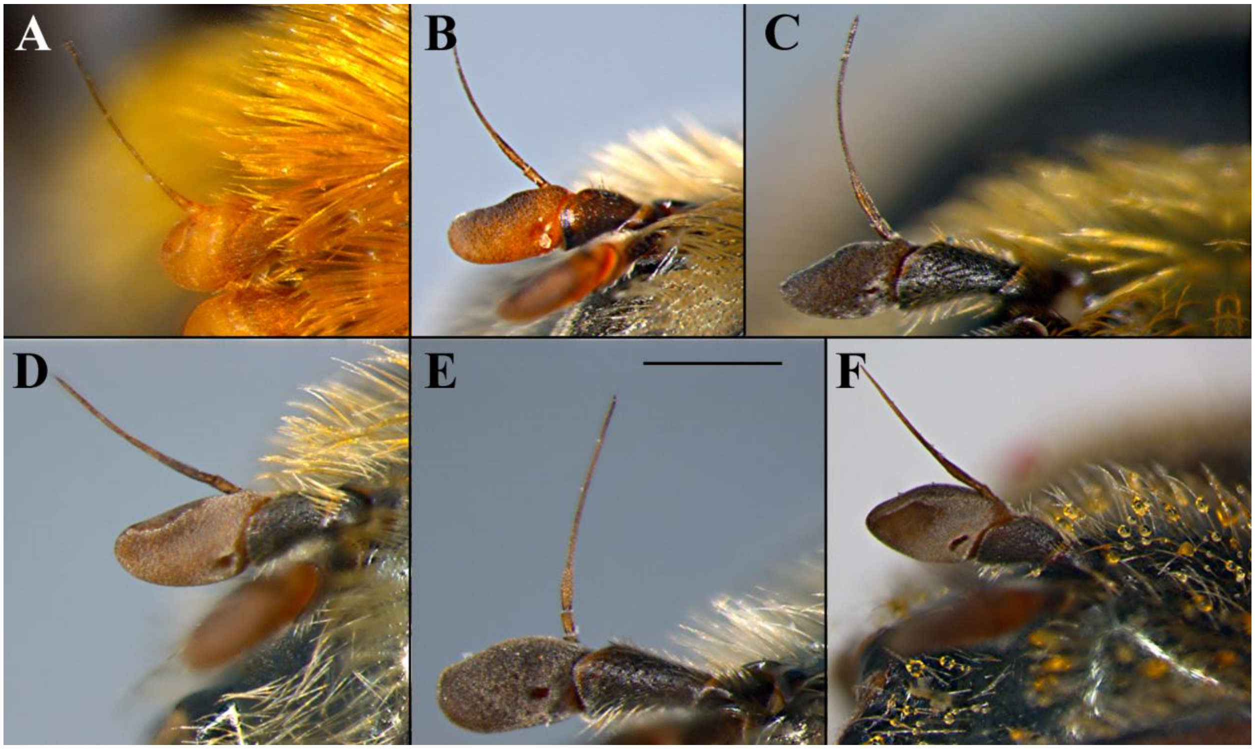

Figure 29. Antenna, lateral view. **A** *Merodon bombiformis*, male **B** *M. nanus*, male **C** *M. funestus*, male **D** *M. aureus*, male **E** *M. funestus*, female **F** *M. aureus*, female. Scale bar: 2 mm.

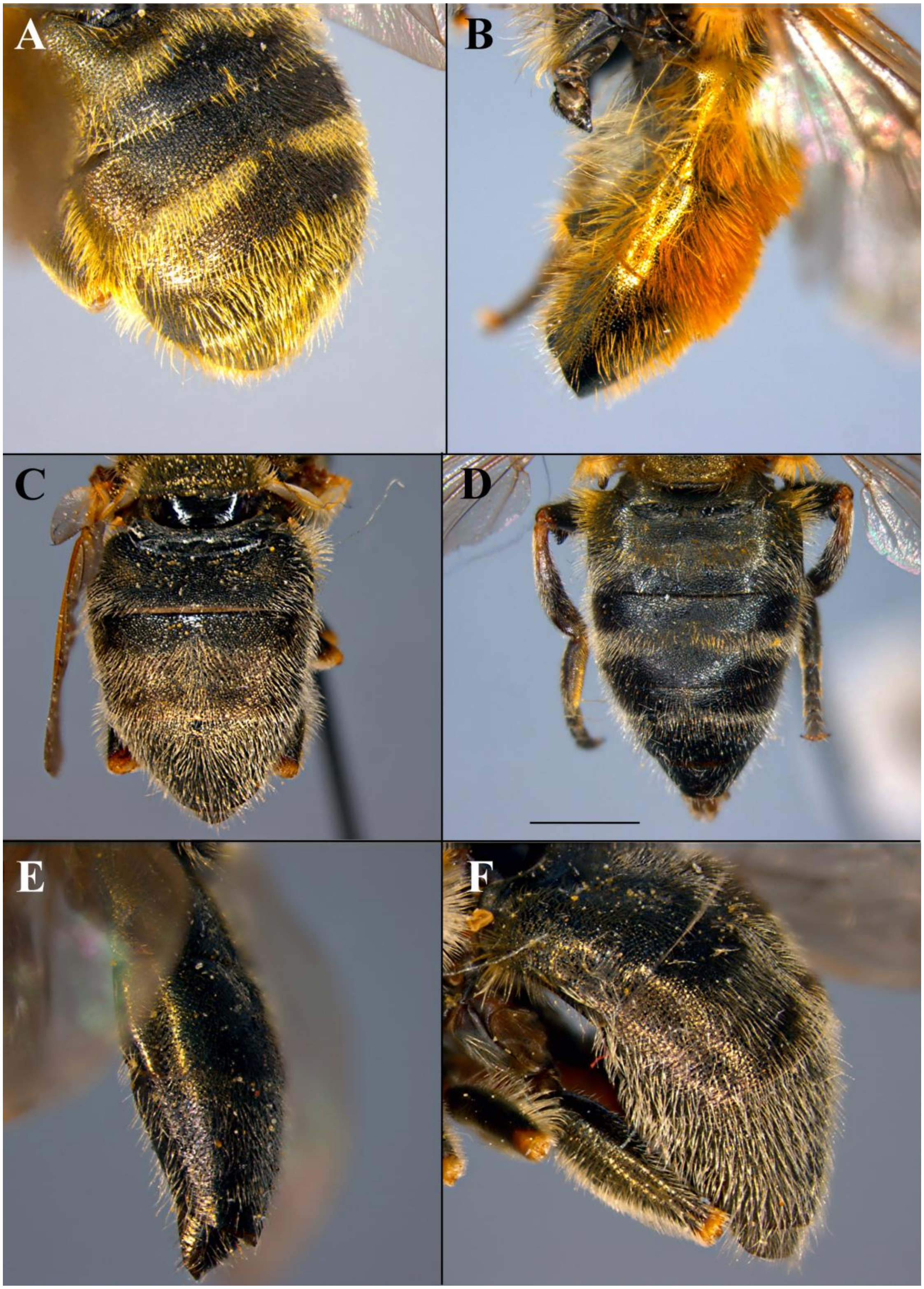

Figure 30. Abdomen. **A** *Merodon funestus*, male, dorsolateral view **B** *M. aureus*, male, lateral view **C** *M. nanus*, female, dorsal view **D** *M. aureus*, female, dorsal view **E** *M. spinitarsis*, female, lateral view **F** *M. nanus*, female, lateral view. Scale bar: 2 mm.

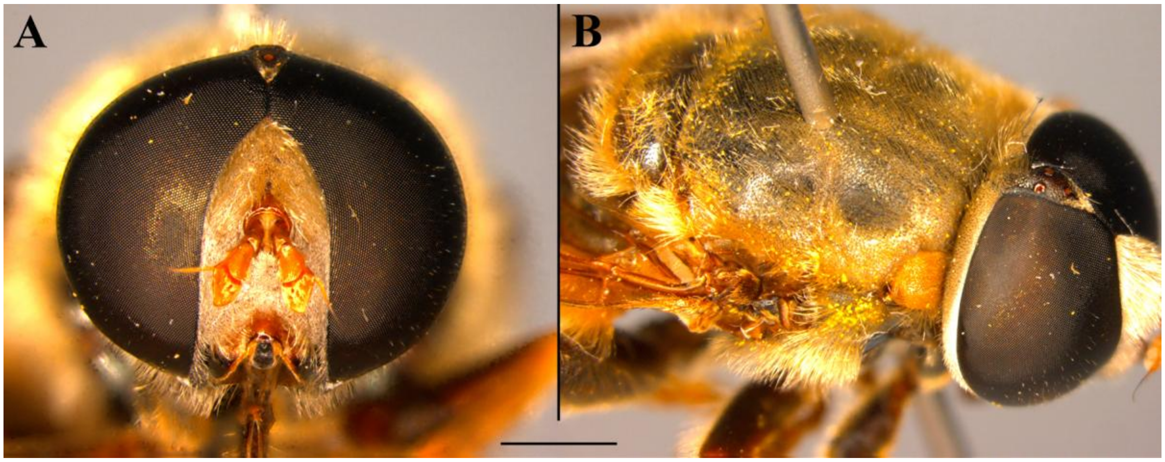

Figure 31. *Merodon luteihumerus*, male. **A** head, anterior view **B** thorax, dorsolateral view. Scale bar: 2 mm.

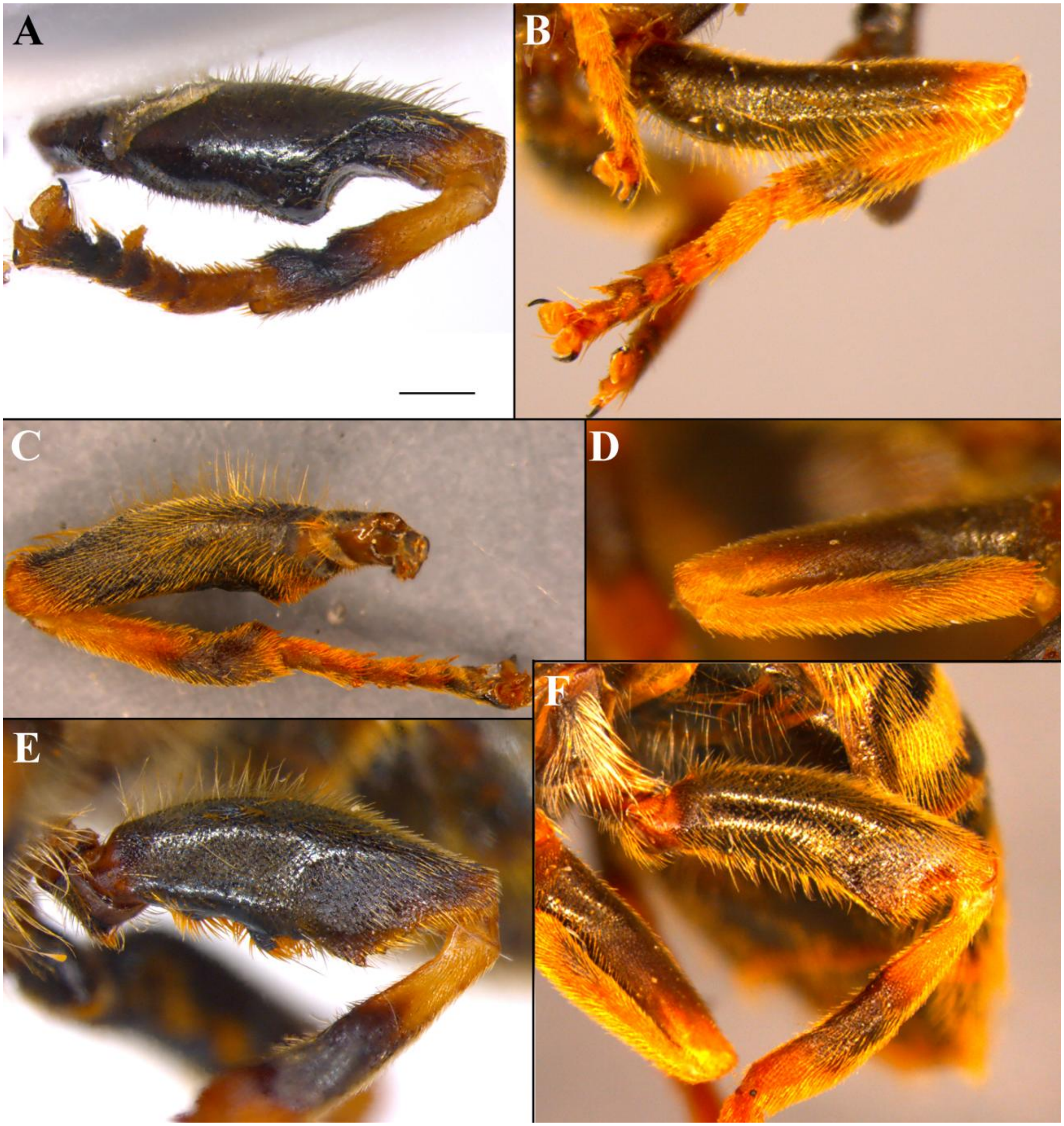

Figure 32. *Merodon mixtum*, legs, lateral view. **A** proleg, male **B** proleg, female **C** mesoleg, male **D** mesoleg, female **E** metaleg, male **F** metaleg, female. Scale bar: 1 mm.

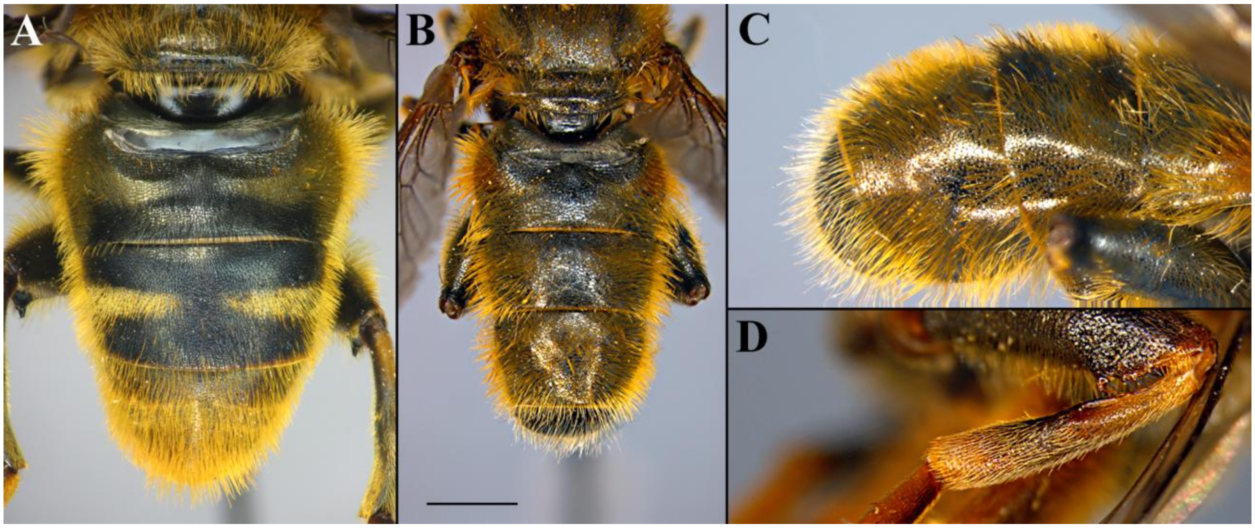

Figure 33. **A–C** Abdomen of male **D** Metatibia. **A** *Merodon gudaurensis* Portschinsky, 1877, dorsal view **B** *M. rufus*, dorsal view **C** *M. rufus*, lateral view **D** *M. albifrons*, lateral view. Scale bar: 2 mm.

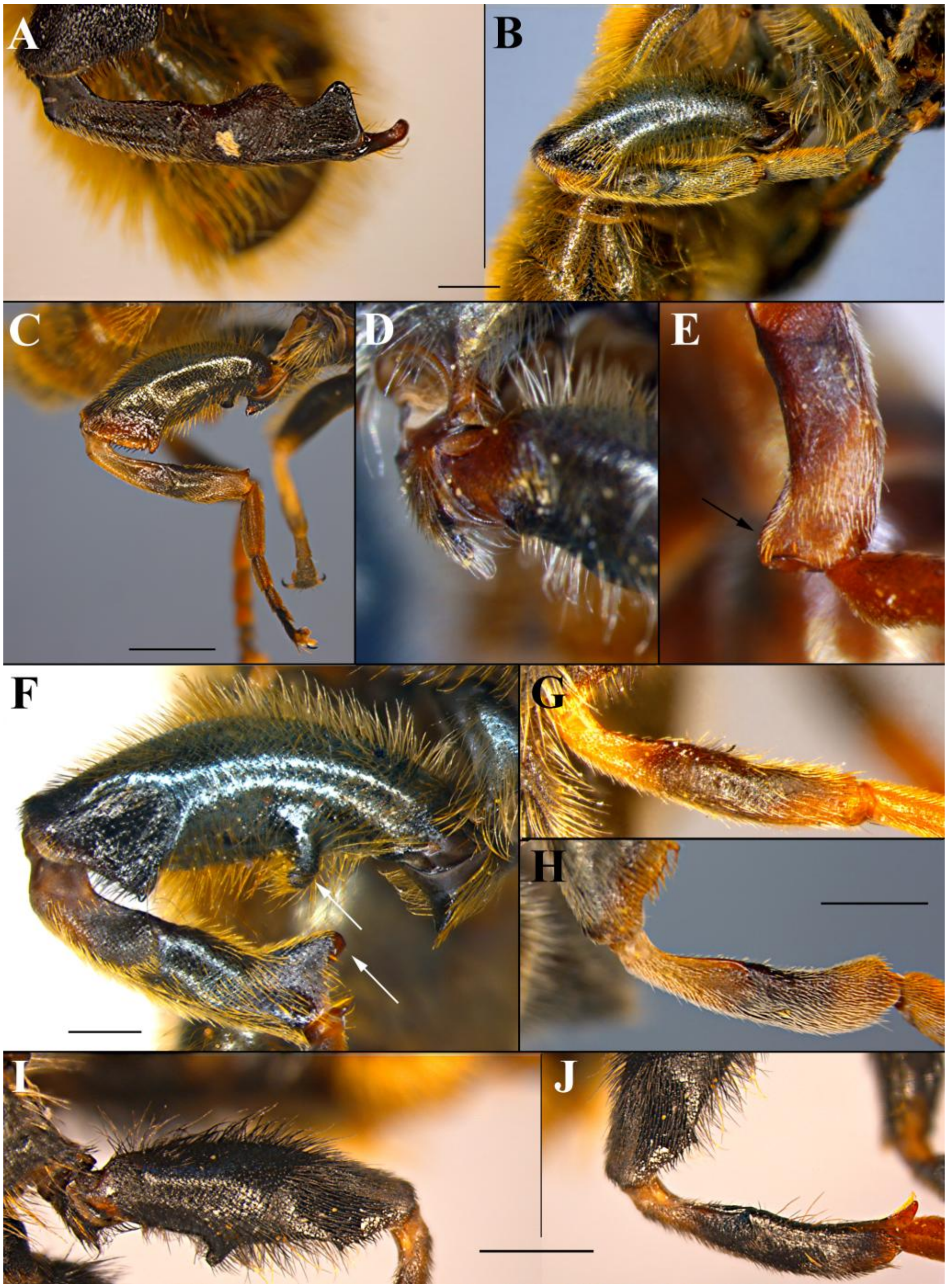

Figure 34. Parts of metaleg, lateral view. **A** *Merodon equestris*, male, metatibia **B** *M. rufus*, male, metaleg **C** *M. trochantericus*, male, metaleg **D** *M. albifasciatus*, male, metatrochanter **E** *M. albifasciatus*, male, metatibia **F** *M. trebevicensis* Strobl, 1900, male, metatrochanter, metafemur and metatibia **G** *M. ruficornis* Meigen, 1822, female, metatibia **H** *M. albifasciatus*, female, metatibia **I** *M. gudaurensis*, male, metafemur **J** *M. gudaurensis*, male, metatibia. **E** apicomedial carina marked with arrow **F** ventral tubercle on metafemur and apicolateral process on metatibia marked with arrow. Scale bar: 0.5 mm (**A–B**); 2 mm (**C**); 1 mm (**D–E**); 2 mm (**F**); 2 mm (**G–H**); 2 mm (**I-J**).

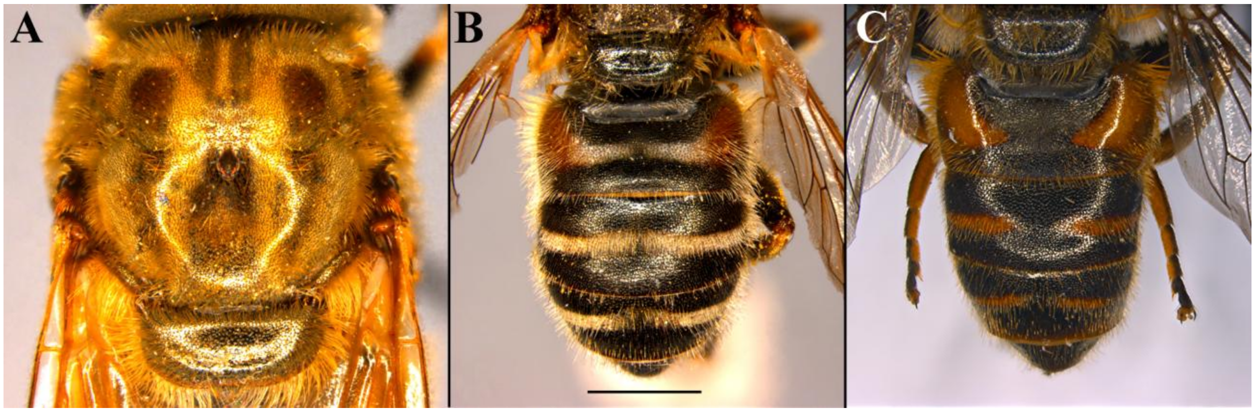

Figure 35. **A** *Merodon albifasciatus*, female, thorax, dorsal view **B** *M. albifasciatus*, female, abdomen, dorsal view **C** *M. albifrons*, female, abdomen, dorsal view. Scale bar: 2 mm.

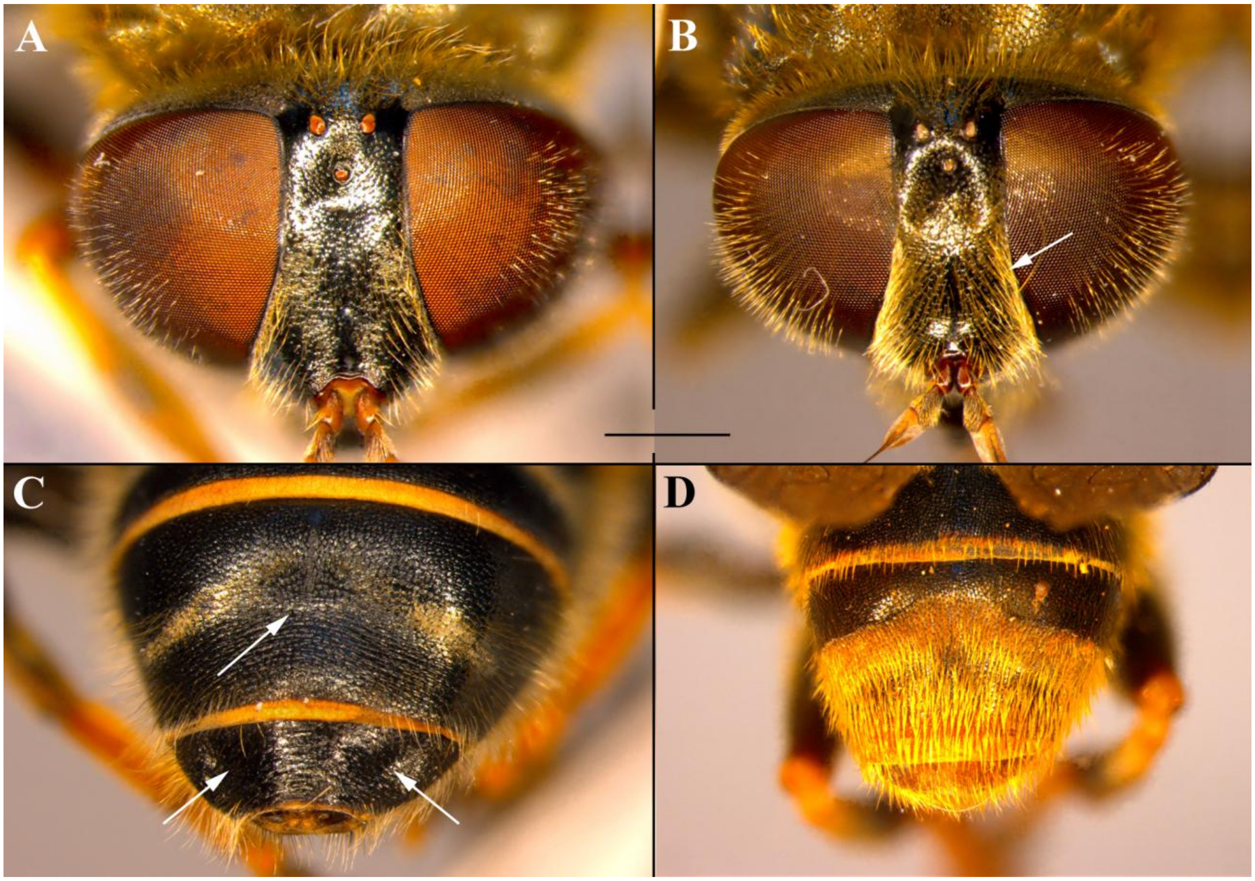

Figure 36. **A–B** Head of female, dorsal view **C–D** Tip of abdomen, dorsal view. **A** *Merodon ruficornis* **B** *M. rufus* **C** *M. ruficornis*, female **D** *M. mixtum*, male. **B** pollinosity along eye margin marked with arrow **C** transversal depression on tergum 4 and lateral depressions on tergum 5 marked with arrow. Scale bar: 2 mm.

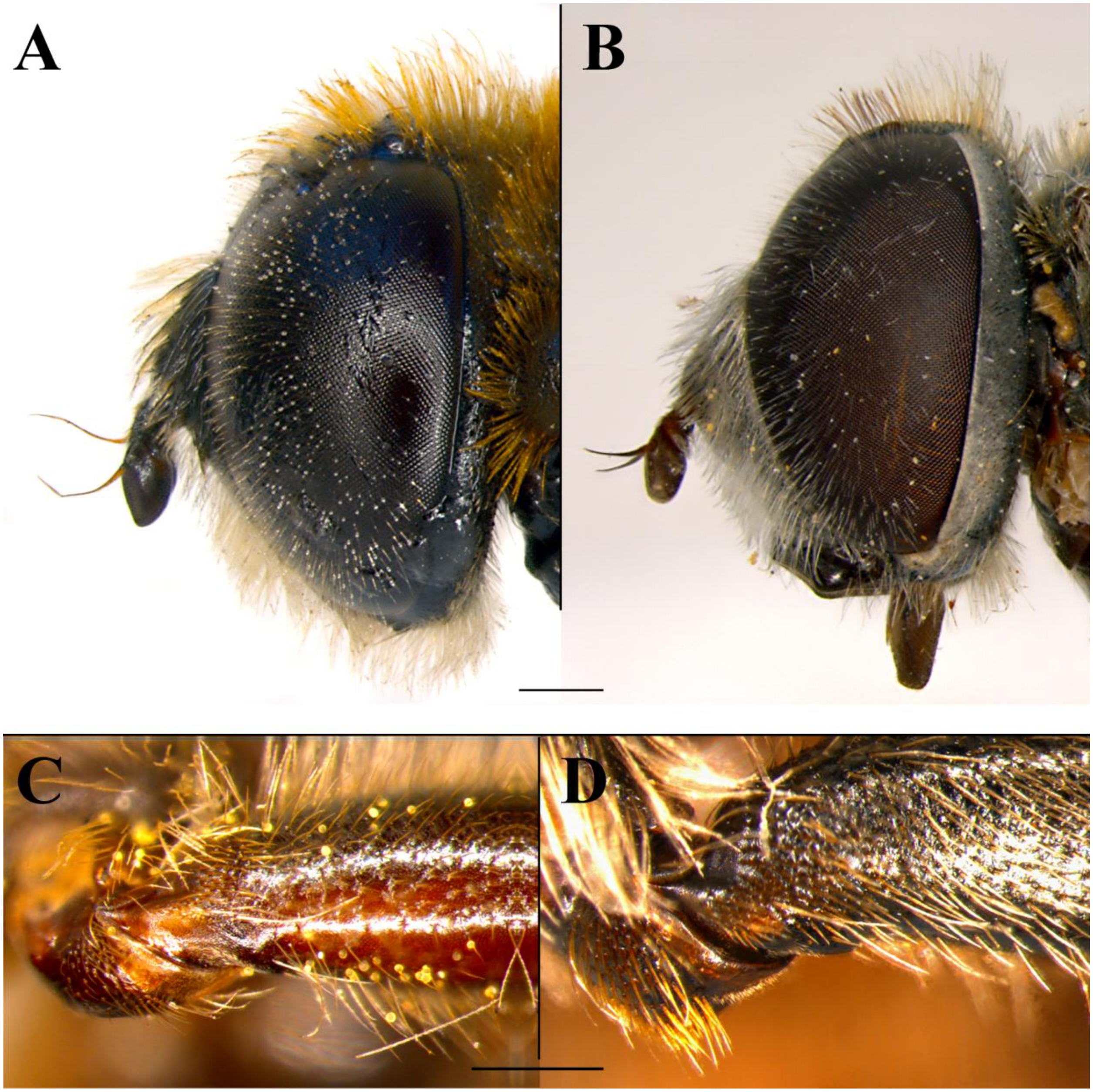

Figure 37. **A–B** Head of male, lateral view **C–D** Metatrochanter of male, lateral view. **A** *Merodon planifacies* **B** *M. neolydicus* **C** *M. desuturinus* **D** *M. draconis* Vujić & Radenković, 2018. Scale bar: 1 mm (**A–B**); 2 mm (**C–D**).
