# Supplementary material for: Taxonomic complexity in the genus Merodon Meigen, 1803 (Diptera, Syrphidae)
Source: Zookeys. 2021 Apr 14;1031:85–124. doi: 10.3897/zookeys.1031.62125 (PMC8060246; doi:10.3897/zookeys.1031.62125)
Supplement: Supplementary material 2 — Figures S1–S13: Figures of male genitalia [file zookeys-1031-085-s002.pdf]

Supplementary file S2: Figures of male genitalia

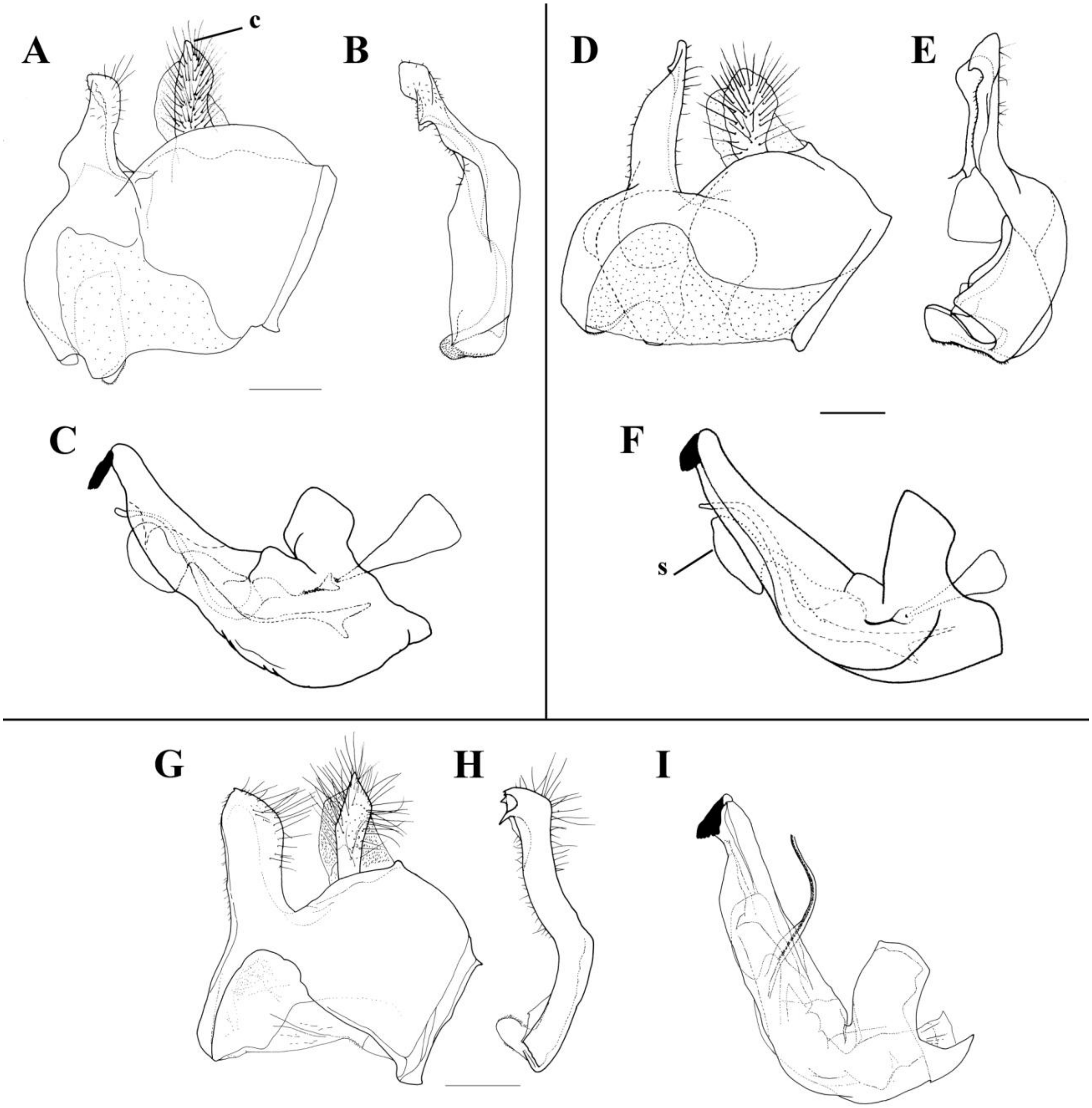

Figure 1. Male genitalia. **A–C** *Merodon constans* (Rossi, 1794) **D–F** *M. chrysotrichos* Vujić, Radenković & Likov, 2020 **G–I** *M. triangulum* Vujić, Radenković & Hurkmans, 2020. **A–B, D–E, G–H** epandrium **C, F, I** hypandrium. **A, C, D, F, G, I** lateral view **B, E, H** ventral view. Abbreviations: c-cercus, s-lateral sclerite of the aedeagus. Scale bar: 0.5 mm.

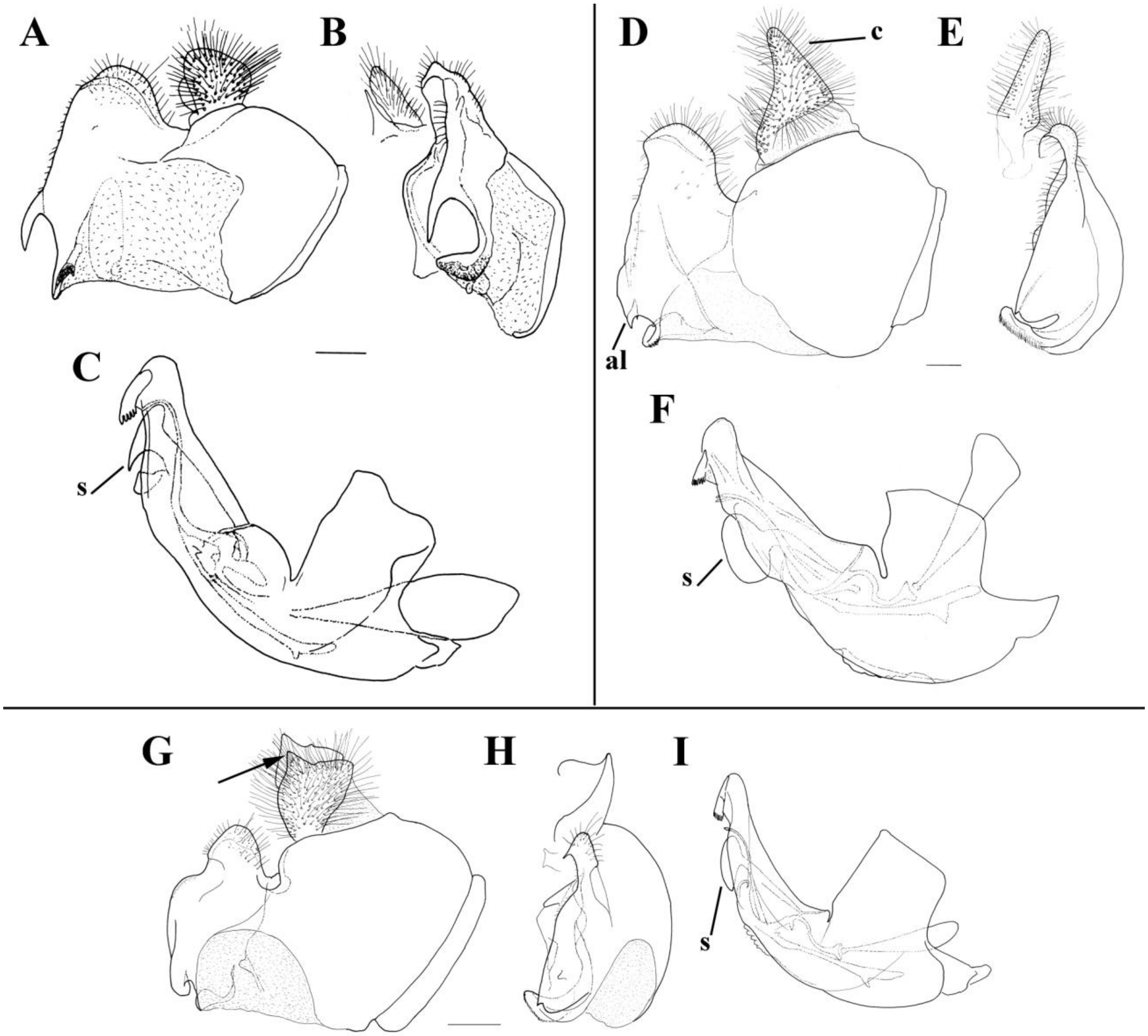

Figure 2. Male genitalia. **A–C** *Merodon albifrons* Meigen, 1822 **D–F** *M. equestris* **G–I** *M. albifasciatus*. **A–B, D–E, G–H** epandrium **C, F, I** hypandrium. **A, C, D, F, G, I** lateral view **B, E, H** ventral view. Abbreviations: al-anterior surstyle lobe, c-cercus, s-lateral sclerite of the aedeagus. Scale bar: 0.4 mm (**A–C**); 0.2 mm (**D–F**); 0.4 mm (**G–I**).

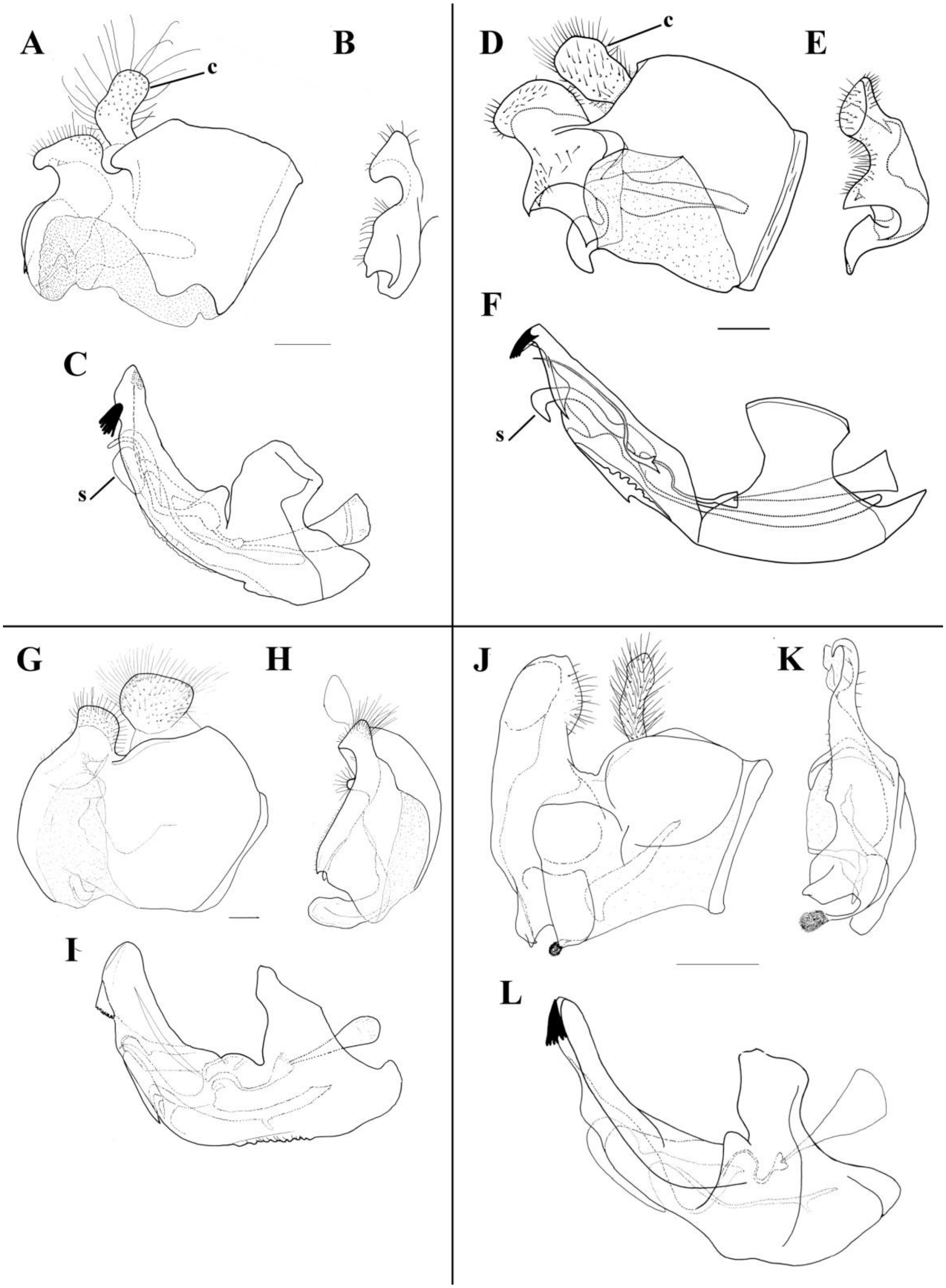

Figure 3. Male genitalia. **A**–**C** *Merodon ruficornis* Meigen, 1822 **D–F** *M. rufus* **G–I** *M. luteihumerus* **J–L** *M. mixtum*. **A–B, D**–**E, G**–**H, J–K** epandrium **C, F, I, L** hypandrium. **A, C, D, F, G, I, J, L** lateral view **B, E, H, K** ventral view. Abbreviations: c-cercus, s-lateral sclerite of the aedeagus. Scale bar: 0.4 mm (**A–C**); 0.25 mm (**D–F**); 0.2 mm (**G–I**); 0.5 mm (**J–L**).

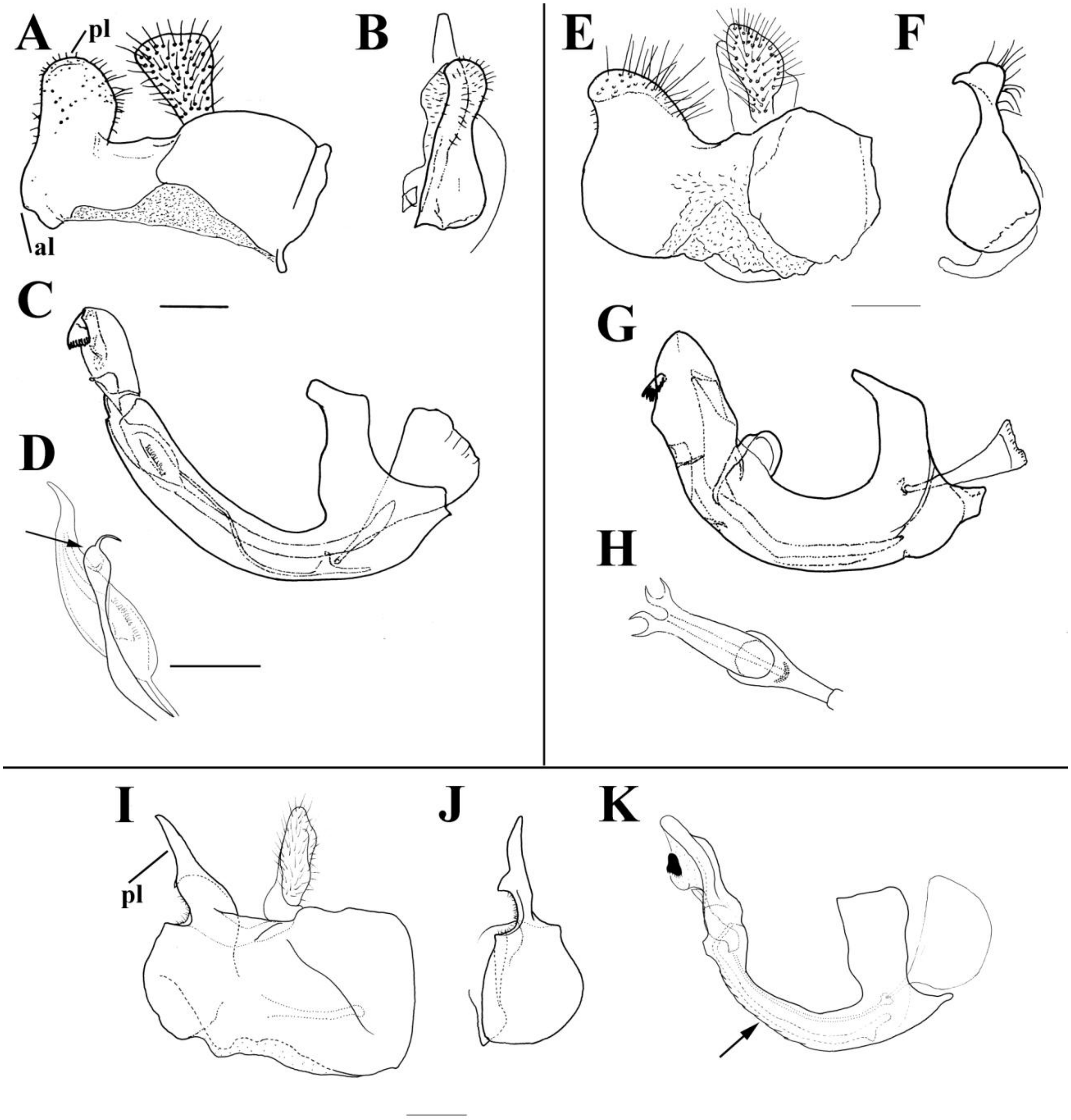

Figure 4. Male genitalia. **A–D** *Merodon aureus* Fabricius, 1805 **E–H** *M. nanus* (Sack, 1931) **I–K** *M. spinitarsis*. **A, B, E, F, I, J** epandrium **C, G, K** hypandrium **D, H** part of aedeagus. **A, C, D, E, G, I, K** lateral view **B, F, H, J** ventral view. Abbreviations: al-anterior surstyle lobe, pl-posterior surstyle lobe. **D** place of lateral sclerite of the aedeagus marked with arrow. Scale bar: 0.25 mm (**A–D**); 0.25 mm (**E–H**); 0.5 mm (**I–K**).

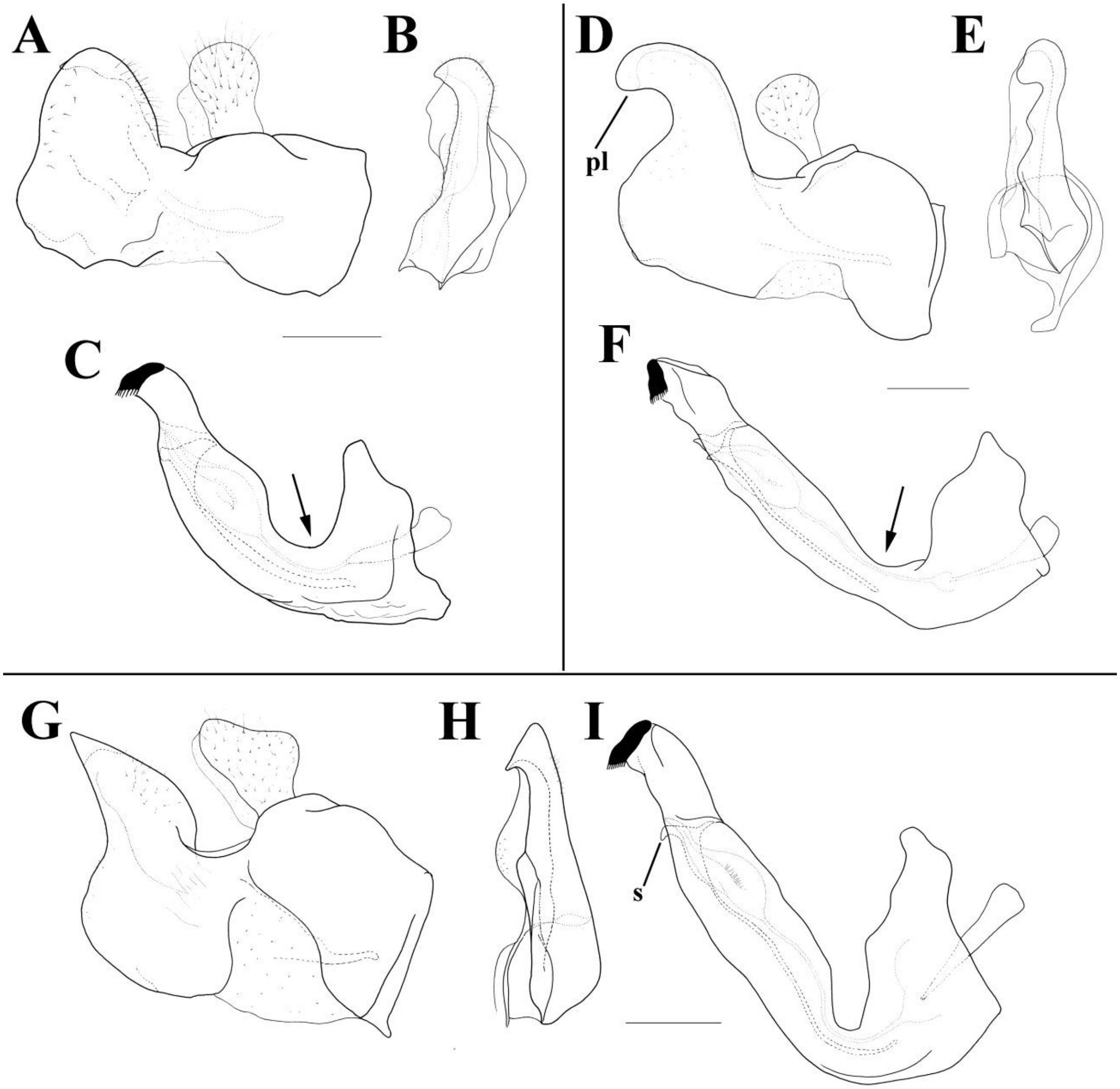

Figure 5. Male genitalia. **A–C** *Merodon bombiformis* **D–F** *M. nasicus* **G–I** *M. funestus*. **A–B, D–E, G–H** epandrium **C, F, I** hypandrium. **A, C, D, F, G, I** lateral view **B, E, H** ventral view. Abbreviations: pl-posterior surstyle lobe, s-lateral sclerite of the aedeagus. **C, F** medially narrowed hypandrium marked with arrow. Scale bar: 0.5 mm.

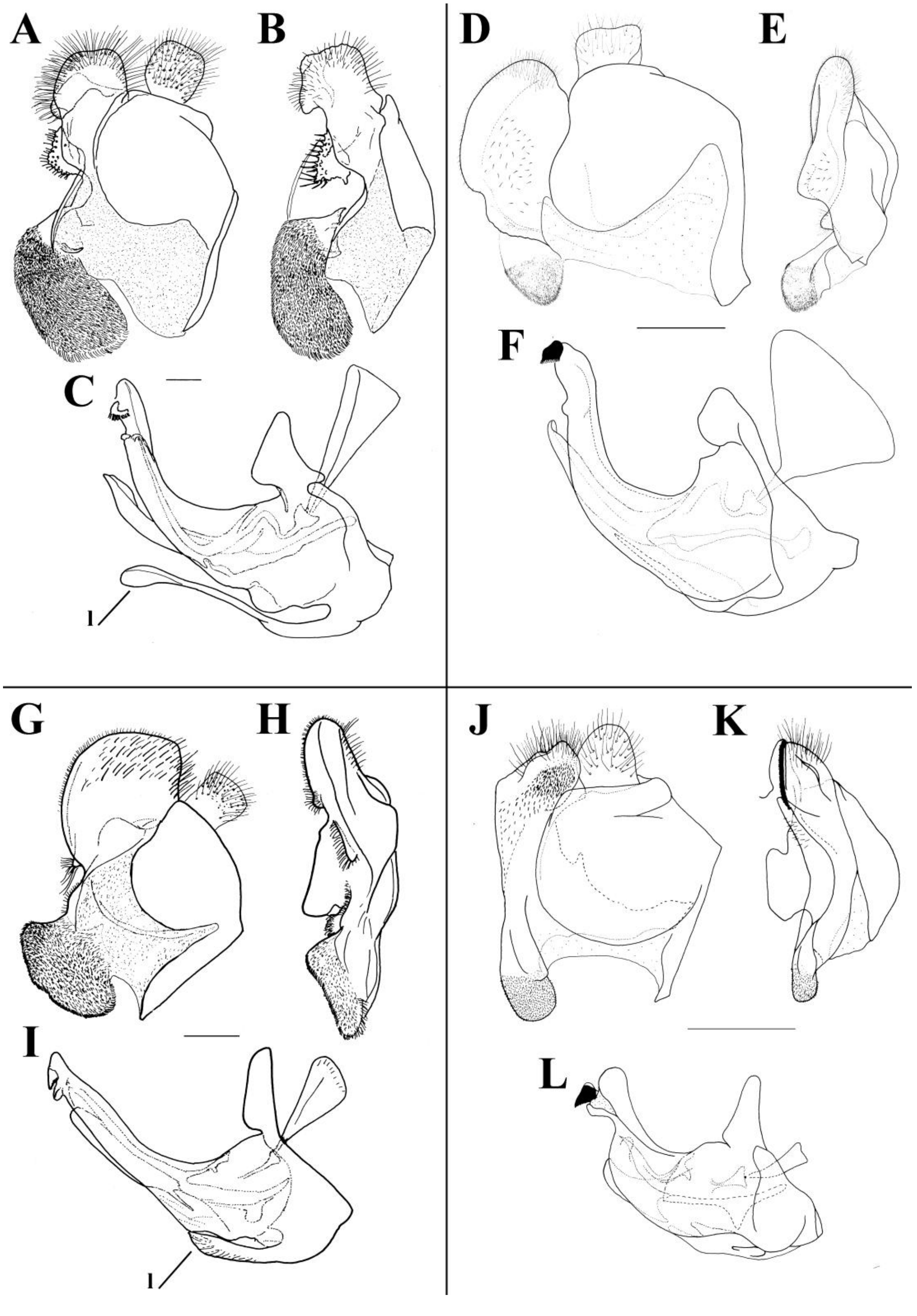

Figure 6. Male genitalia. **A–C** *Merodon aberrans* **D–F** *M. aurifer* **G–I** *M. avidus* (Rossi, 1790) **J–L** *M. rutitarsis*. **A–B, D–E, G–H, J–K** epandrium **C, F, I, L** hypandrium. **A, C, D, F, G, I, J, L** lateral view **B, E, H, K** ventral view. Abbreviations: l-lingula. Scale bar: 0.2 mm (**A–C, G–I**); 0.5 mm (**D–F, J–L**).

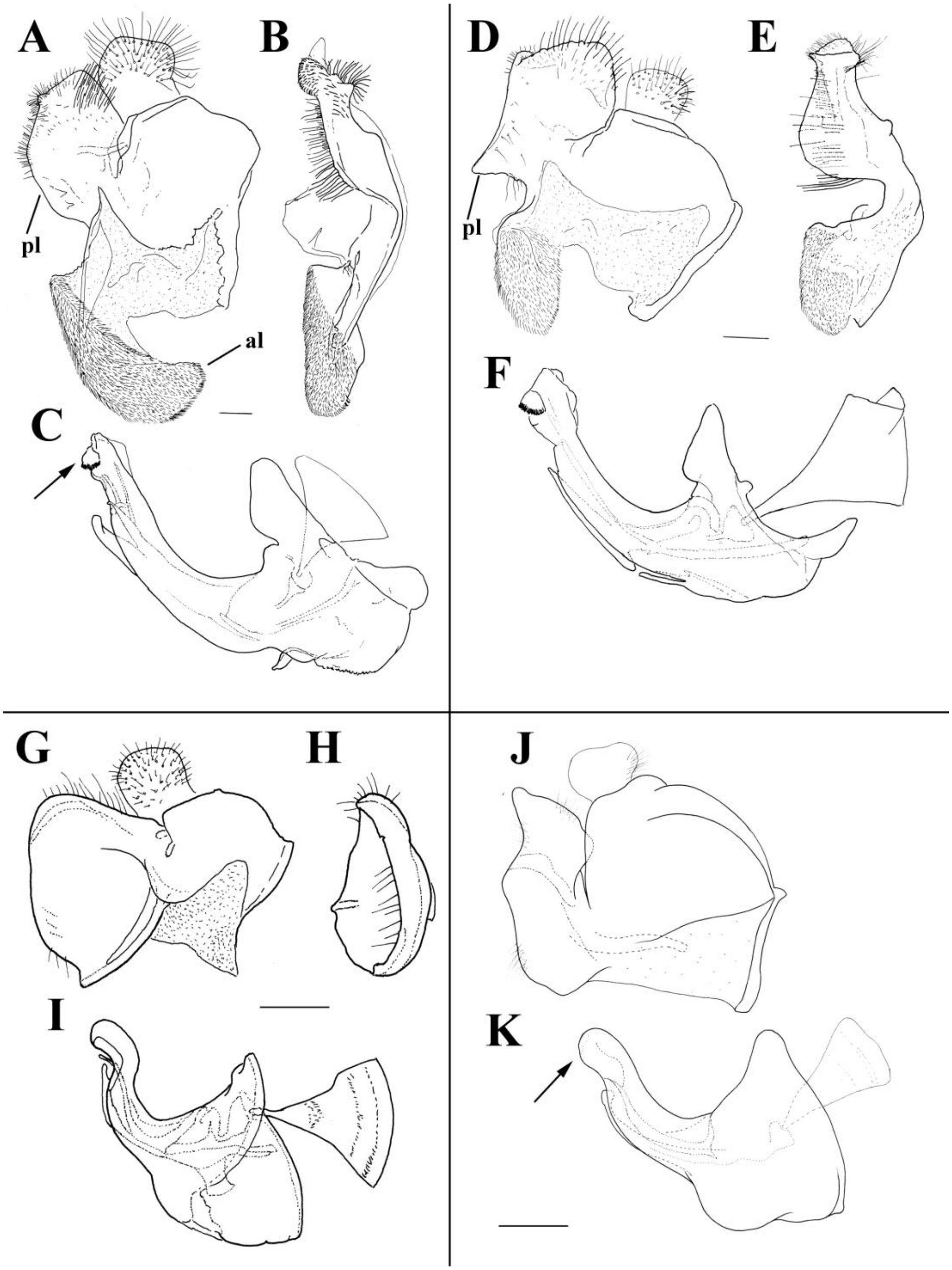

Figure 7. Male genitalia. **A–C** *Merodon clavipes* (Fabricius, 1781) **D–F** *M. italicus* **G–I** *M. dichopticus* **J–K** *M. fulcratus*. **A–B, D–E, G–H, J–K** epandrium **C, F, I, L** hypandrium. **A, C, D, F, G, I, J, L** lateral view **B, E, H, K** ventral view. Abbreviations: al-anterior surstyle lobe, pl-posterior surstyle lobe. **C, K** place of ctenidium marked with arrow. Scale bar: 0.2 mm (**A–F, J–L**); 0.5 mm (**G–I**).

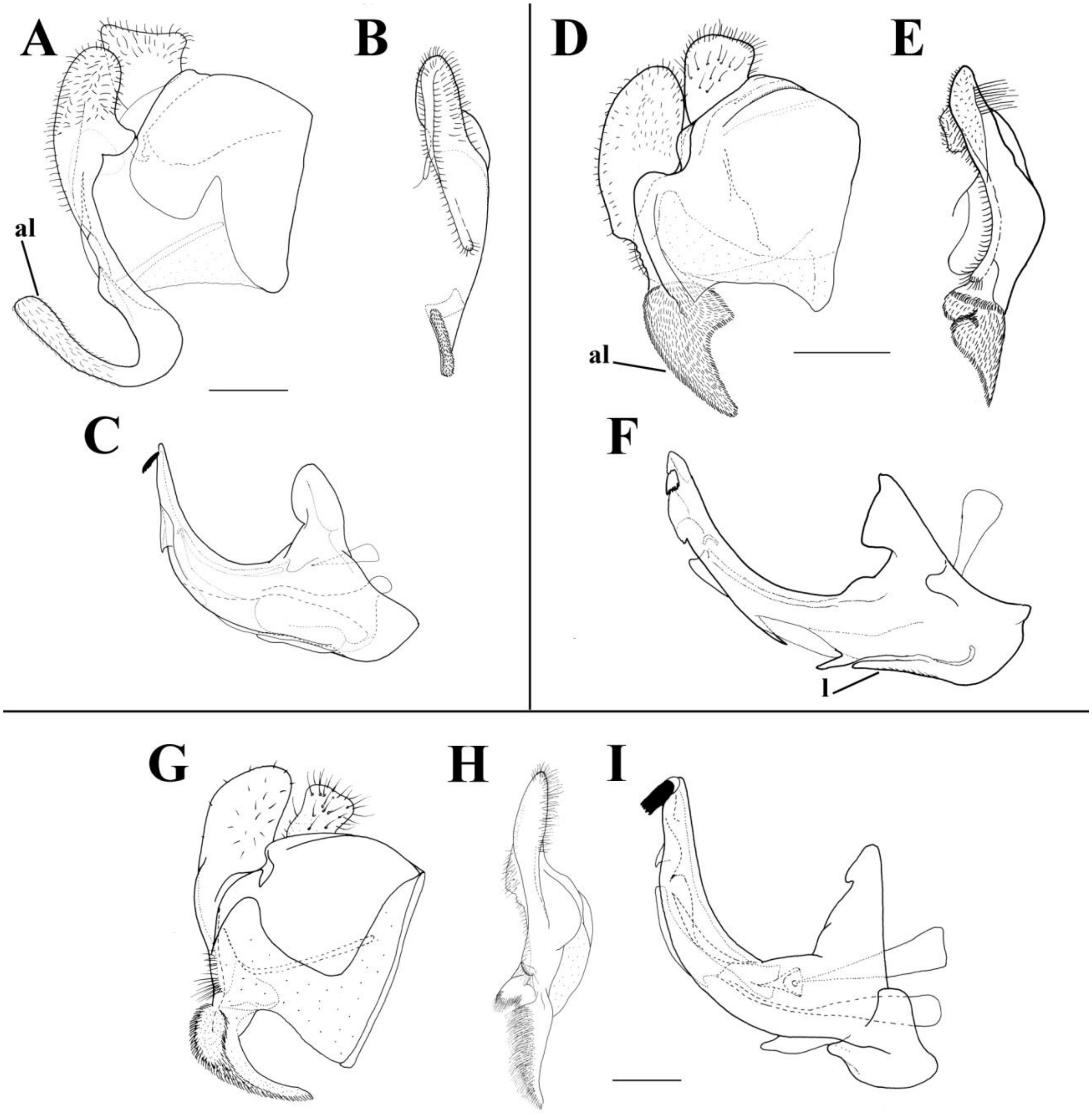

Figure 8. Male genitalia. **A–C** *Merodon alagoesicus* Paramonov, 1925 **D–F** *M. nigritarsis* Rondani, 1845 **G–I** *M. obstipus* Vujić, Radenković & Likov, 2020. **A–B, D–E, G–H** epandrium **C, F, I** hypandrium. **A, C, D, F, G, I** lateral view **B, E, H** ventral view. Abbreviations: al- anterior surstyle lobe. Scale bar: 0.5 mm.

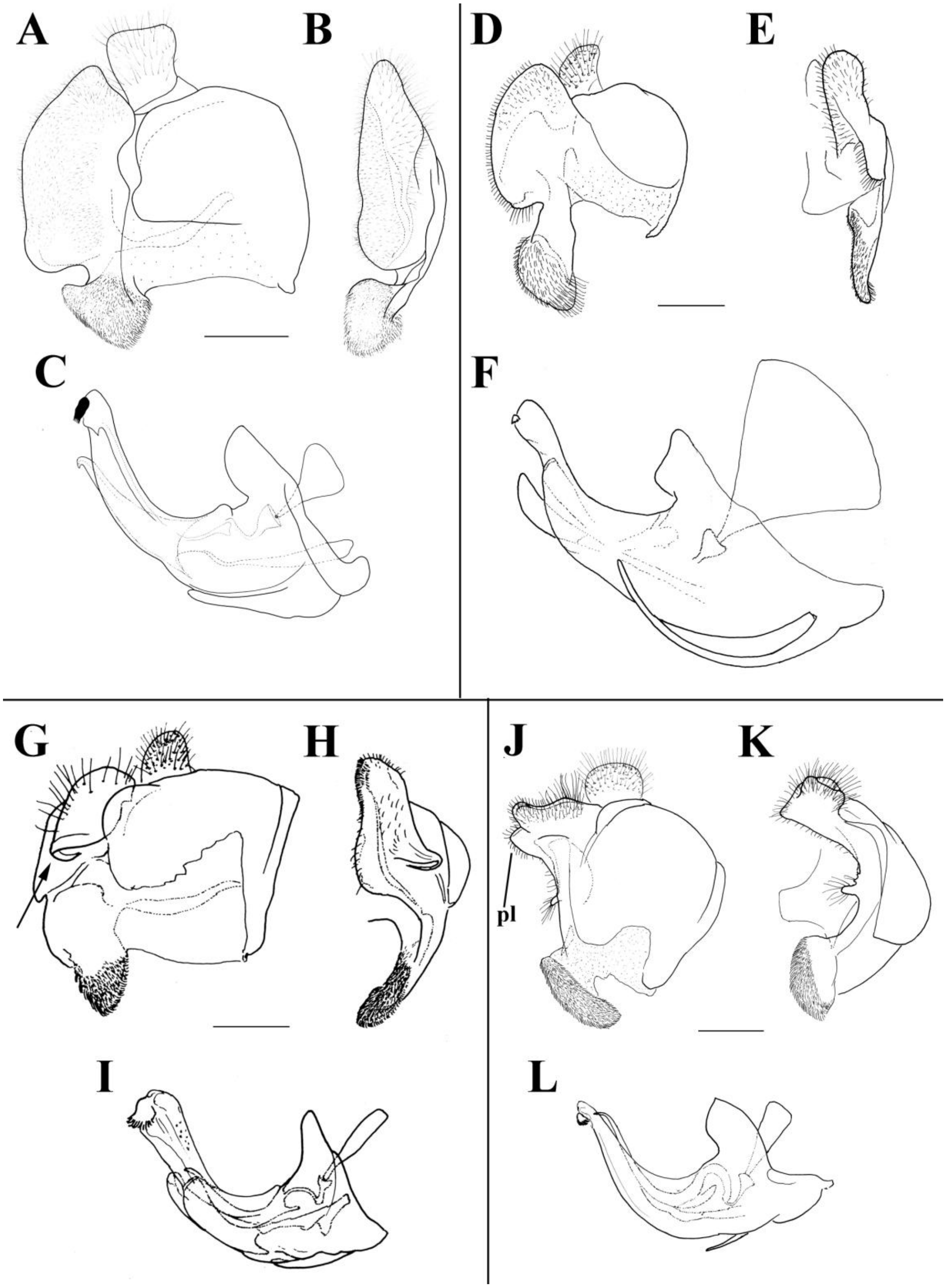

Figure 9. Male genitalia. **A–C** *Merodon pruni* **D–F** *M. hypochrysos* Hurkmans, 1993 **G–I** *M. serrulatus* **J–L** *M. hirtus*. **A–B, D–E, G–H, J–K** epandrium **C, F, I, L** hypandrium. **A, C, D, F, G, I, J, L** lateral view **B, E, H, K** ventral view. Abbreviations: pl-posterior surstyle lobe. **G** basolateral protrusion marked with arrow. Scale bar: 0.5 mm (**A–C**); 0.2 mm (**D–L**).

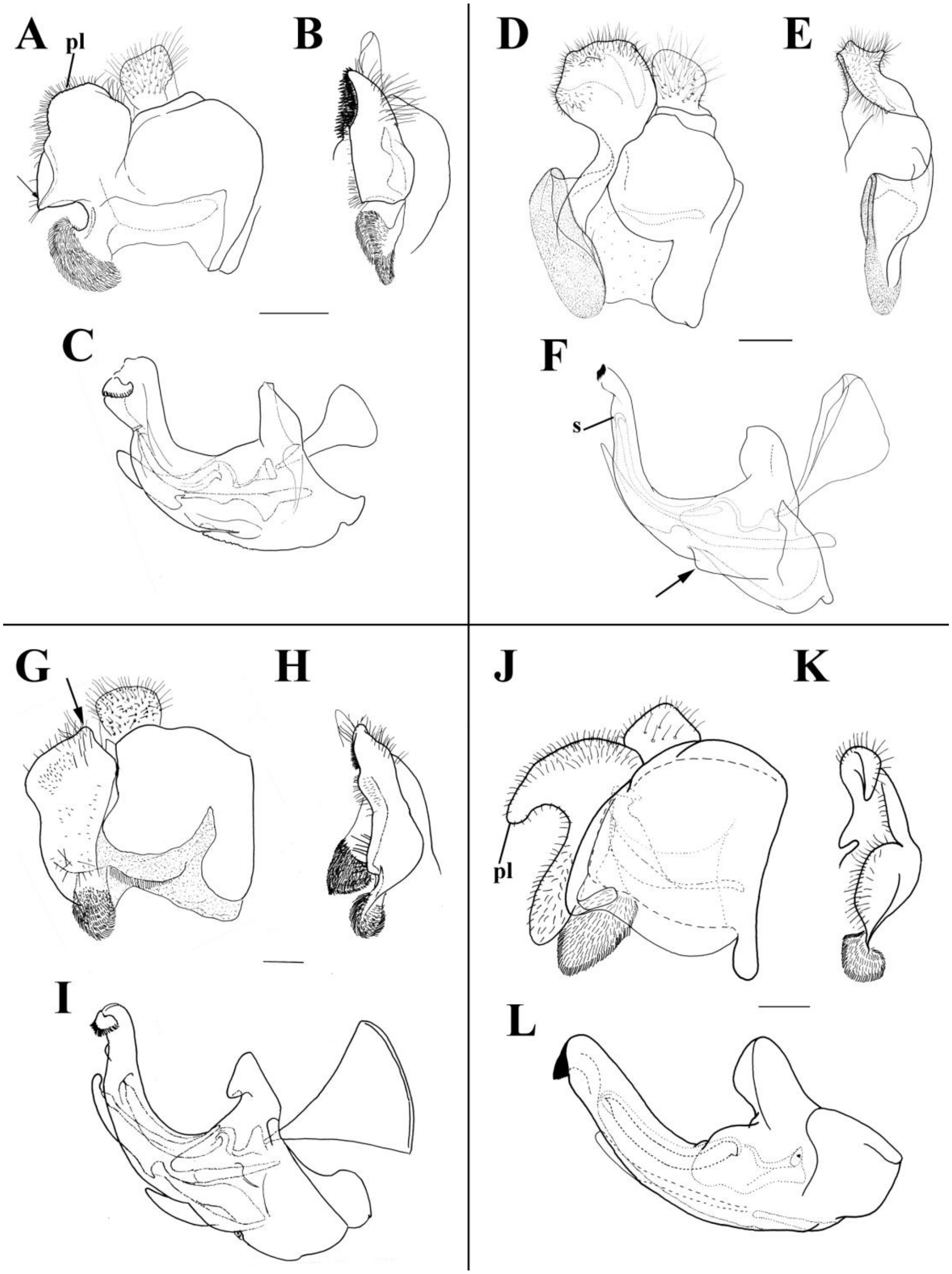

Figure 10. Male genitalia. **A–C** *Merodon auronitens* **D–F** *M. caudatus* **G–I** *M. clunipes* **J–L** *M. crassifemoris*. **A–B, D–E, G–H, J–K** epandrium **C, F, I, L** hypandrium. **A, C, D, F, G, I, J, L** lateral view **B, E, H, K** ventral view. Abbreviations: pl-posterior surstyle lobe, s-lateral sclerite of the aedeagus. **A** triangular basal extension marked with arrow **F** ventral processes of the hypandrium marked with arrow **G** apical hump marked with arrow. Scale bar: 0.2 mm (**A–C, G–L**); 0.5 mm (**D–F**).

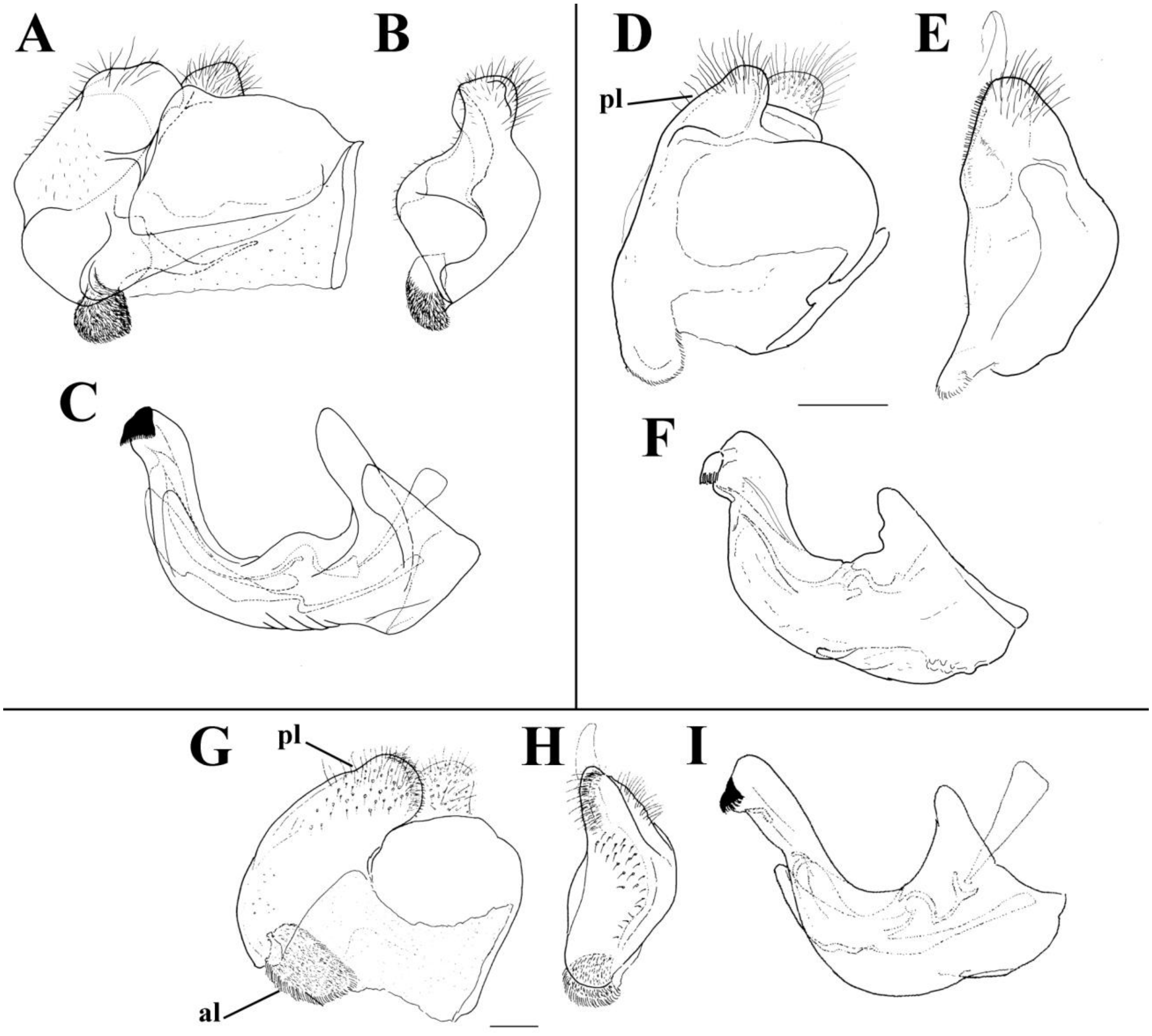

Figure 11. Male genitalia. **A–C** *Merodon eumerusi* **D–F** *M. murinus* **G–I** *M. ottomanus*. **A–B, D–E, G–H** epandrium **C, F, I** hypandrium. **A, C, D, F, G, I** lateral view **B, E, H** ventral view. Abbreviations: al-anterior surstyle lobe, pl-posterior surstyle lobe. Scale bar: 0.2 mm.

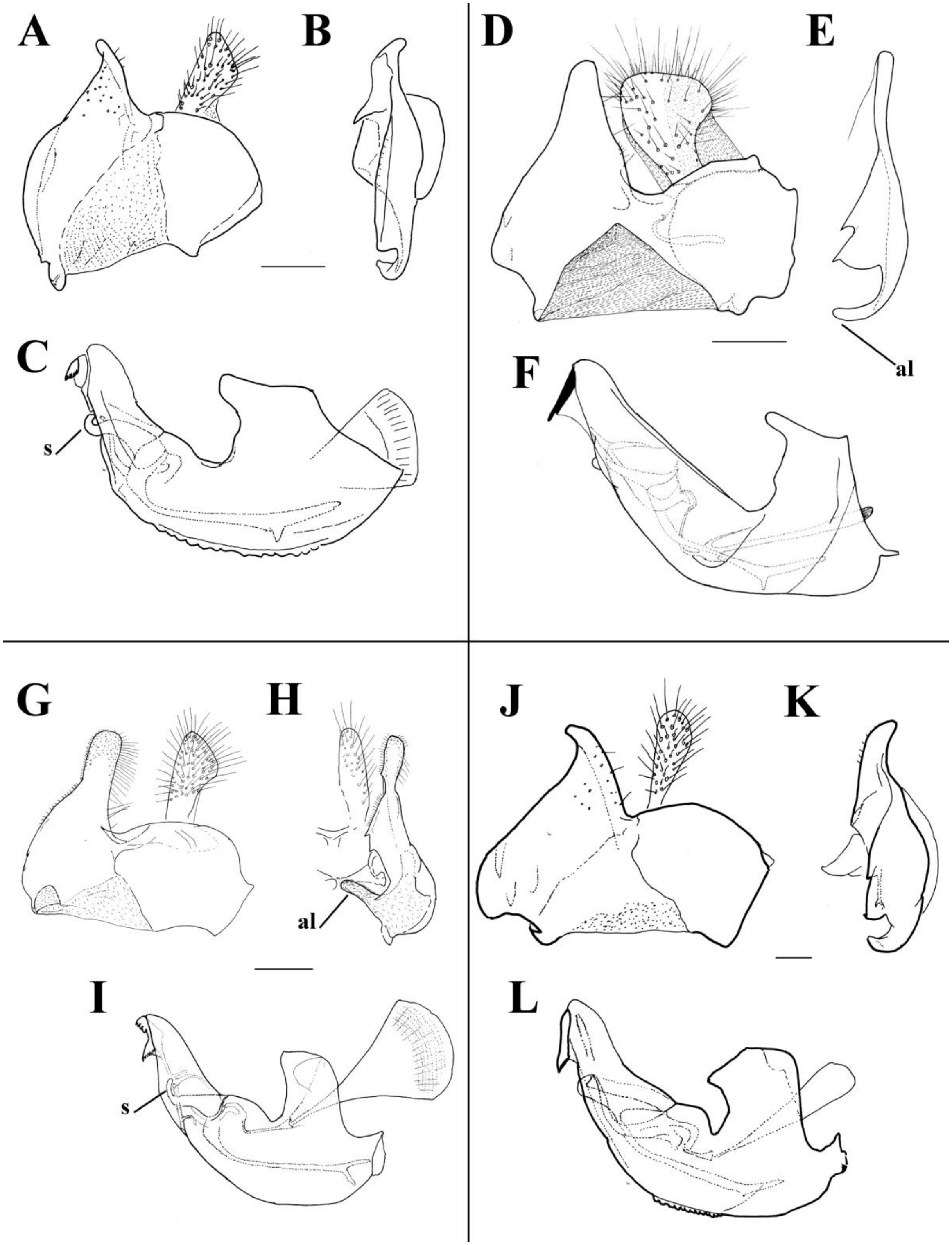

Figure 12. Male genitalia. **A–C** *Merodon capensis* **D–F** *M. desuturinus* **G–I** *M. neolydicus* **J–L** *M. planifacies*. **A–B, D–E, G–H, J–K** epandrium **C, F, I, L** hypandrium. **A, C, D, F, G, I, J, L** lateral view **B, E, H, K** ventral view. Abbreviations: al-anterior surstyle lobe, s-lateral sclerite of the aedeagus. Scale bar: 0.2 mm.

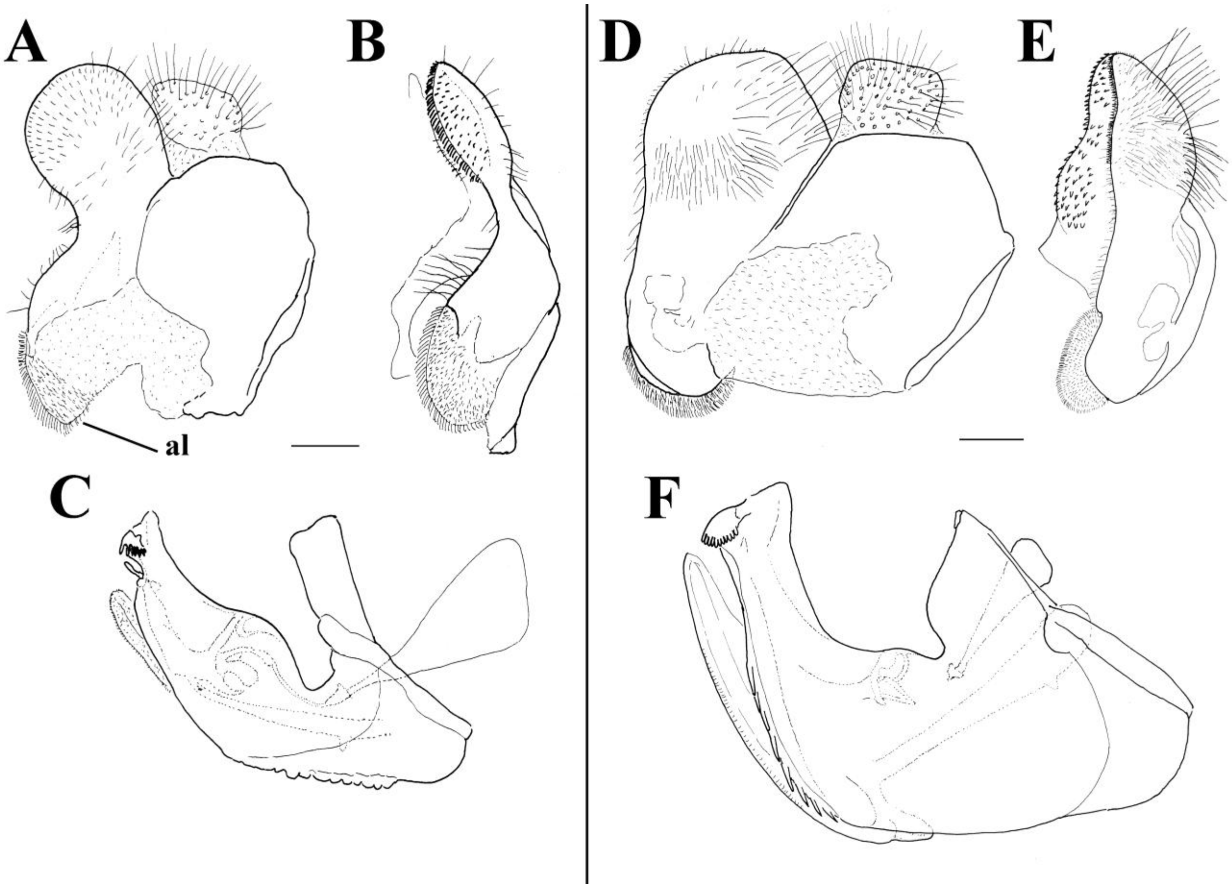

Figure 13. Male genitalia. **A–C** *Merodon natans* (Fabricius, 1794) **D–F** *M. segetum*. **A, B, D, E** epandrium **C, F** hypandrium. **A, C, D, F** lateral view **B, E** ventral view. Abbreviations: al- anterior surstyle lobe. Scale bar: 0.2 mm.
