# Supplementary material for: Taxonomic complexity in the genus Merodon Meigen, 1803 (Diptera, Syrphidae)
Source: Zookeys. 2021 Apr 14;1031:85–124. doi: 10.3897/zookeys.1031.62125 (PMC8060246; doi:10.3897/zookeys.1031.62125)
Supplement: Supplementary material 3 — Figures S1–S15: Figures of adults [file zookeys-1031-085-s003.pdf]

Supplementary file S3: Figures of adults

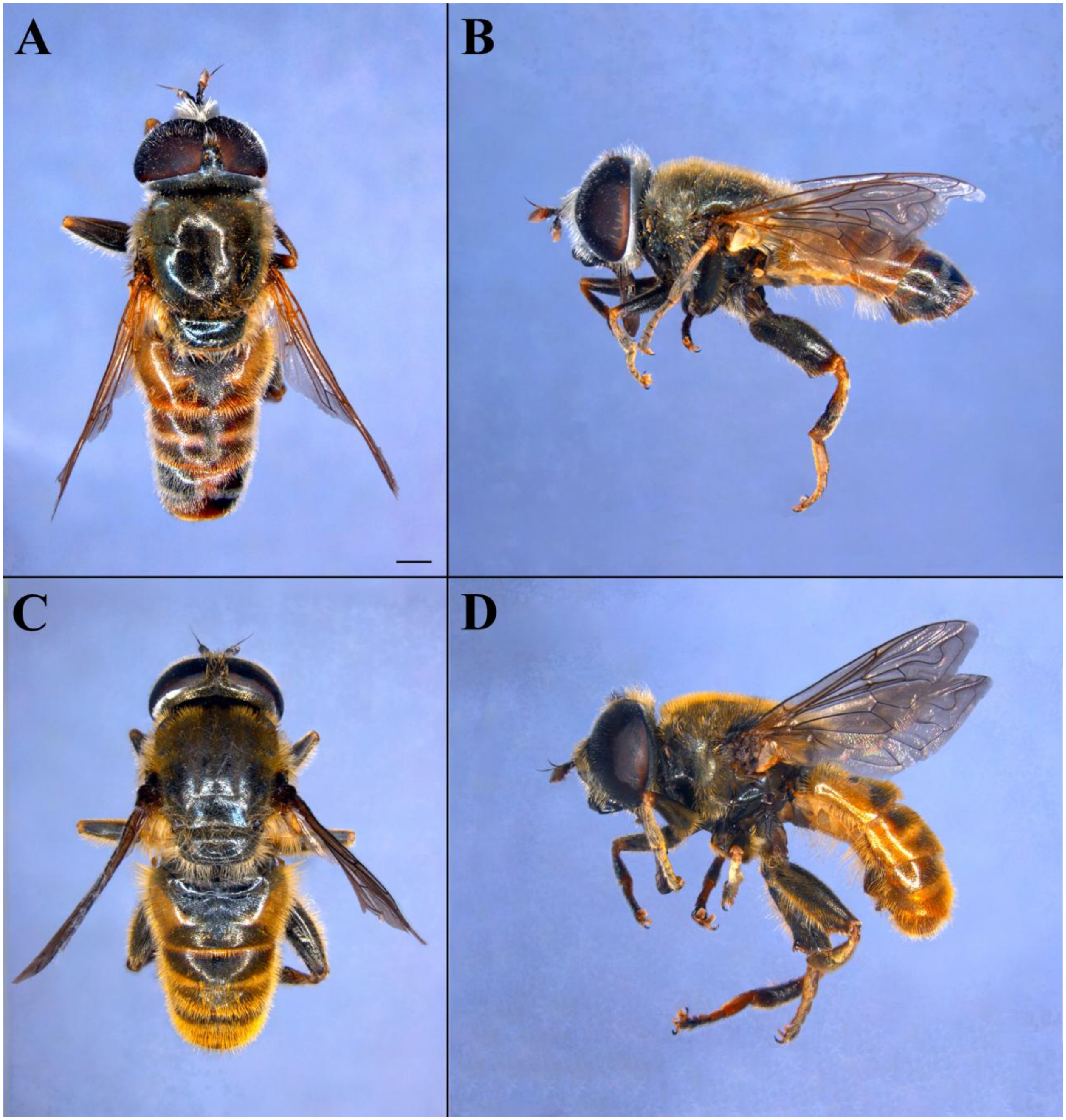

Figure 1. Body of male. **A–B** *Merodon albifrons* Meigen, 1822 **C–D** *M. constans* (Rossi, 1794). **A, C** dorsal view **B, D** lateral view. Scale bar: 1 mm.

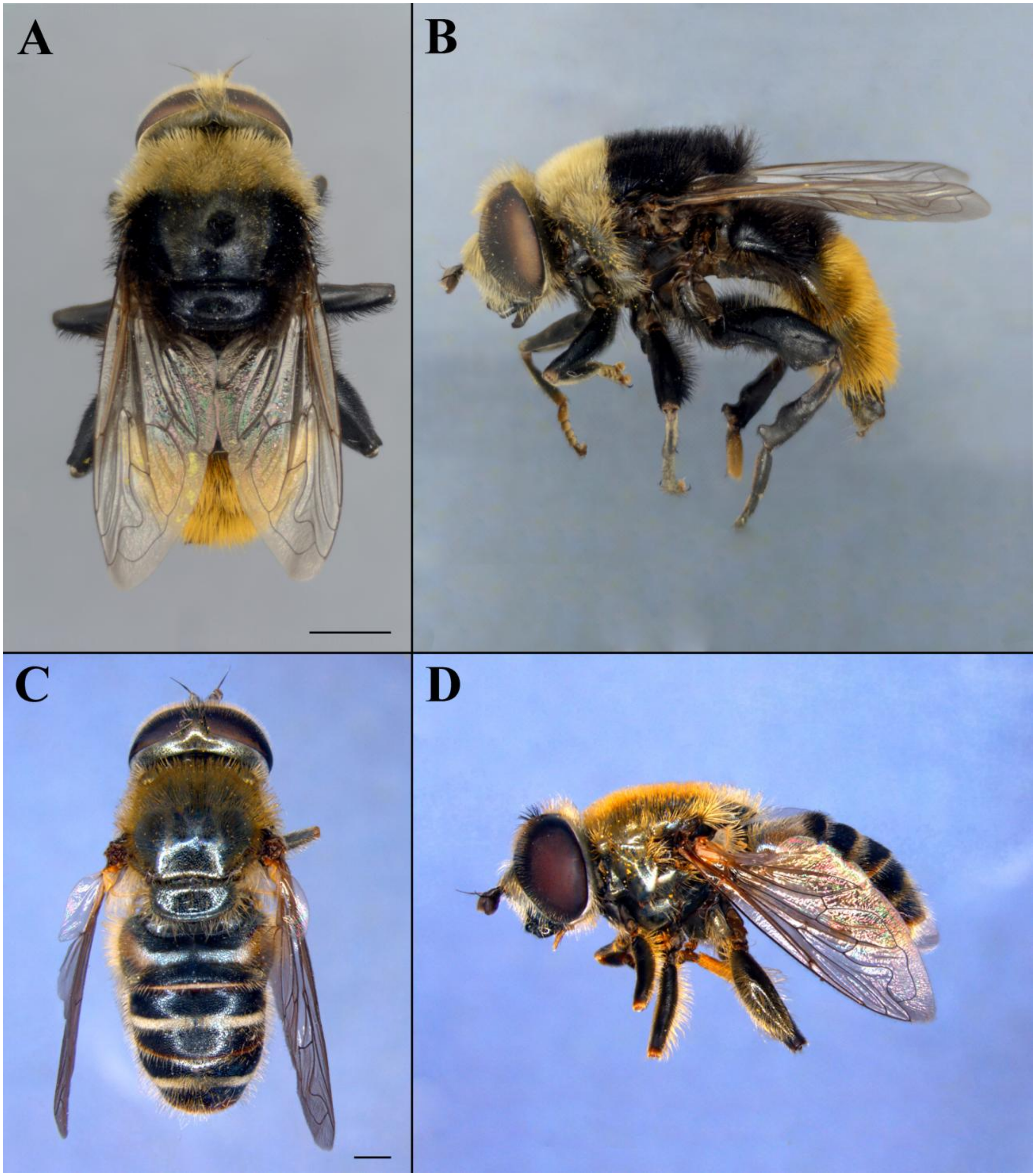

Figure 2. Body of male. **A–B** *Merodon equestris* **C–D** *M. geniculatus*. **A, C** dorsal view **B, D** lateral view. Scale bar: 3 mm (**A–B**); 1 mm (**C–D**).

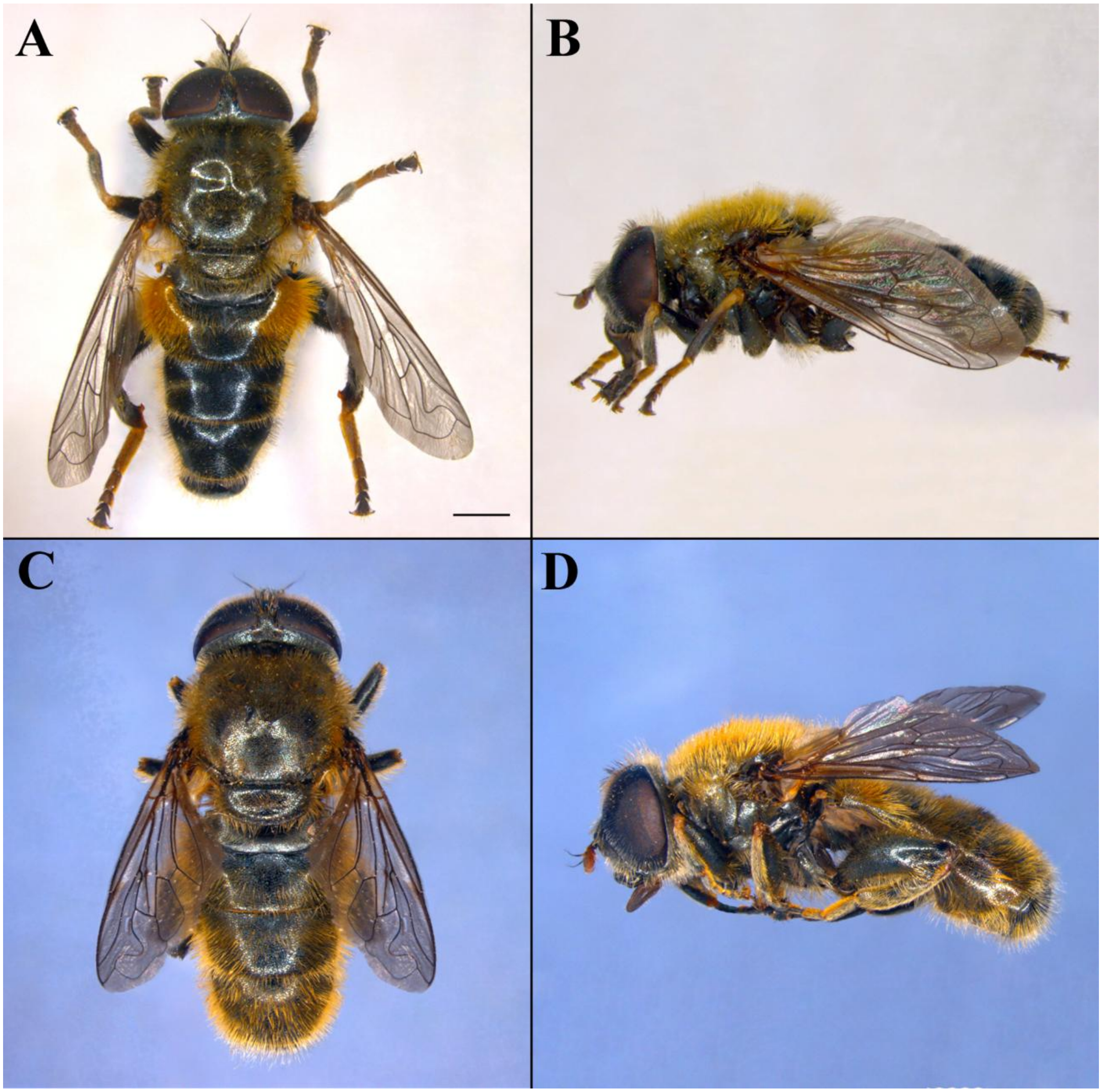

Figure 3. Body of male. **A–B** *Merodon ruficornis* Meigen, 1822 **C–D** *M. rufus*. **A, C** dorsal view **B, D** lateral view. Scale bar: 1.5 mm.

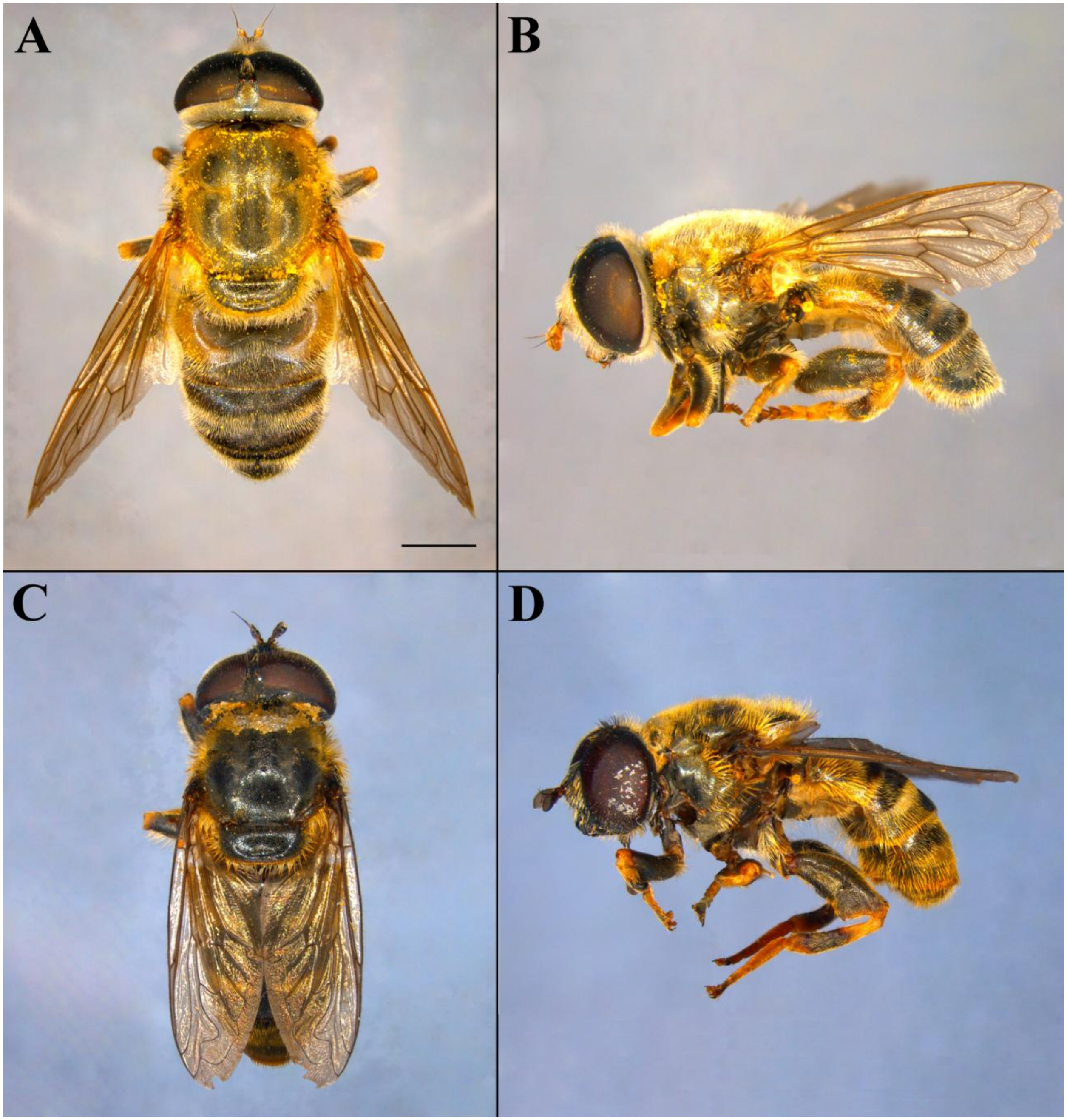

Figure 4. Body of male. **A–B** *Merodon luteihumerus* **C–D** *M. mixtum*. **A, C** dorsal view **B, D** lateral view. Scale bar: 2 mm.

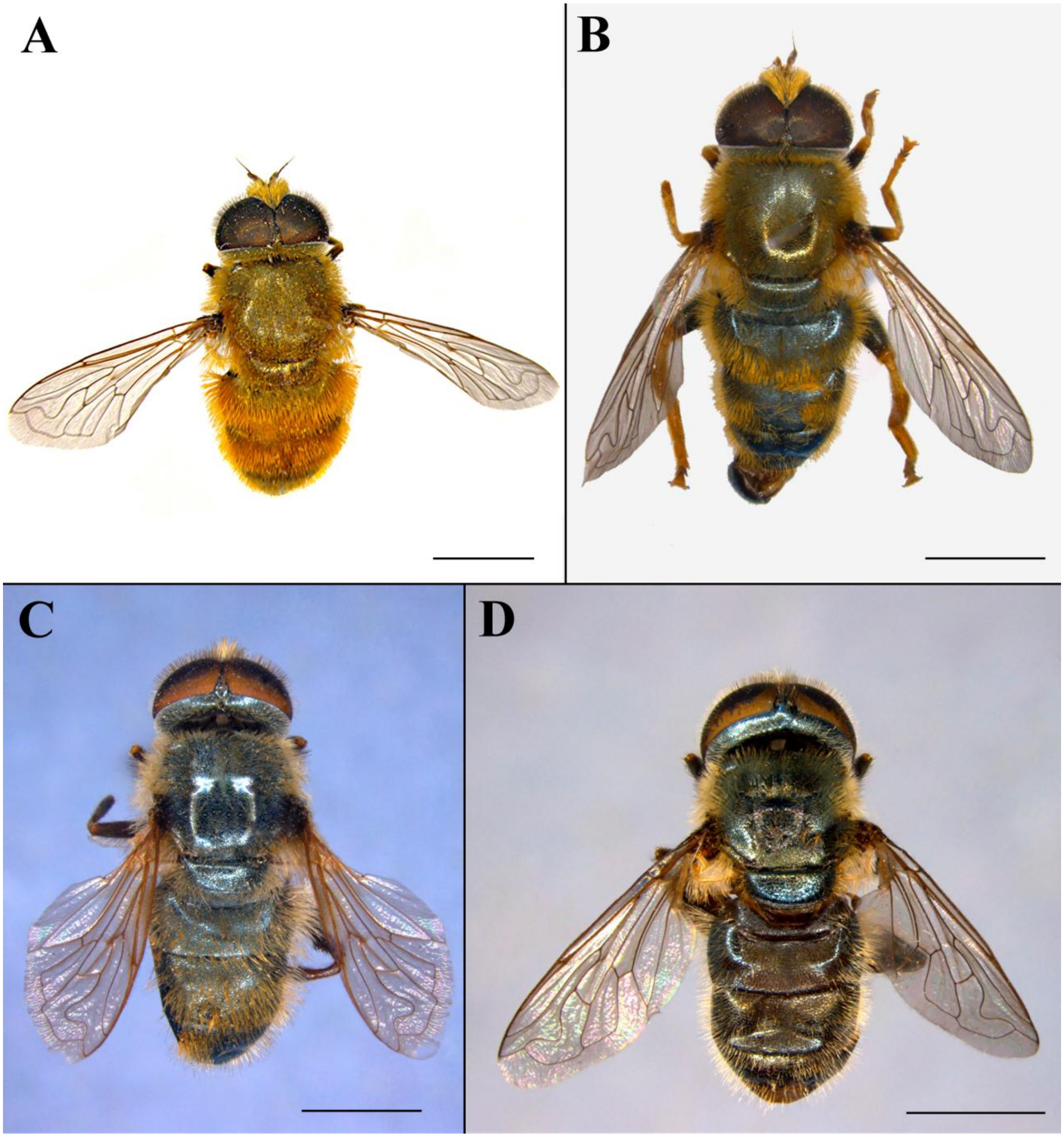

Figure 5. Body of male, dorsal view. **A** *Merodon aureus* Fabricius, 1805 **B** *M. bessarabicus* Paramonov, 1924 **C** *M. cinereus* (Fabricius, 1794) **D** *M. chalybeus* Wiedemann, 1822. Scale bar: 2 mm (**A–C**); 1.5 mm (**D**).

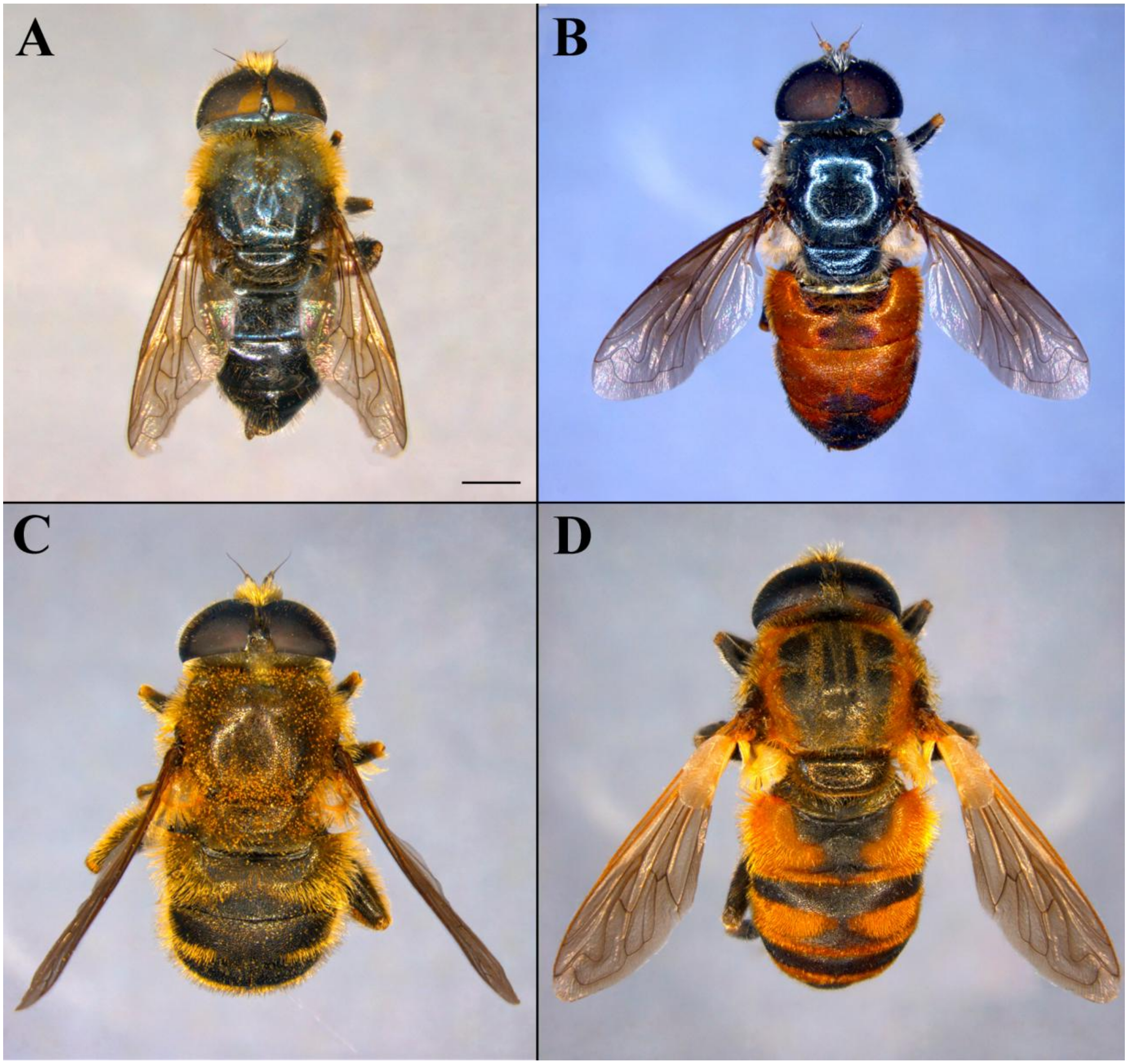

Figure 6. Body of male, dorsal view. **A** *Merodon caerulescens* Loew, 1869 **B** *M. dobrogensis* **C** *M. funestus* **D** *M. bombiformis*. Scale bar: 1.5 mm (**A–B**), 2 mm (**C–D**).

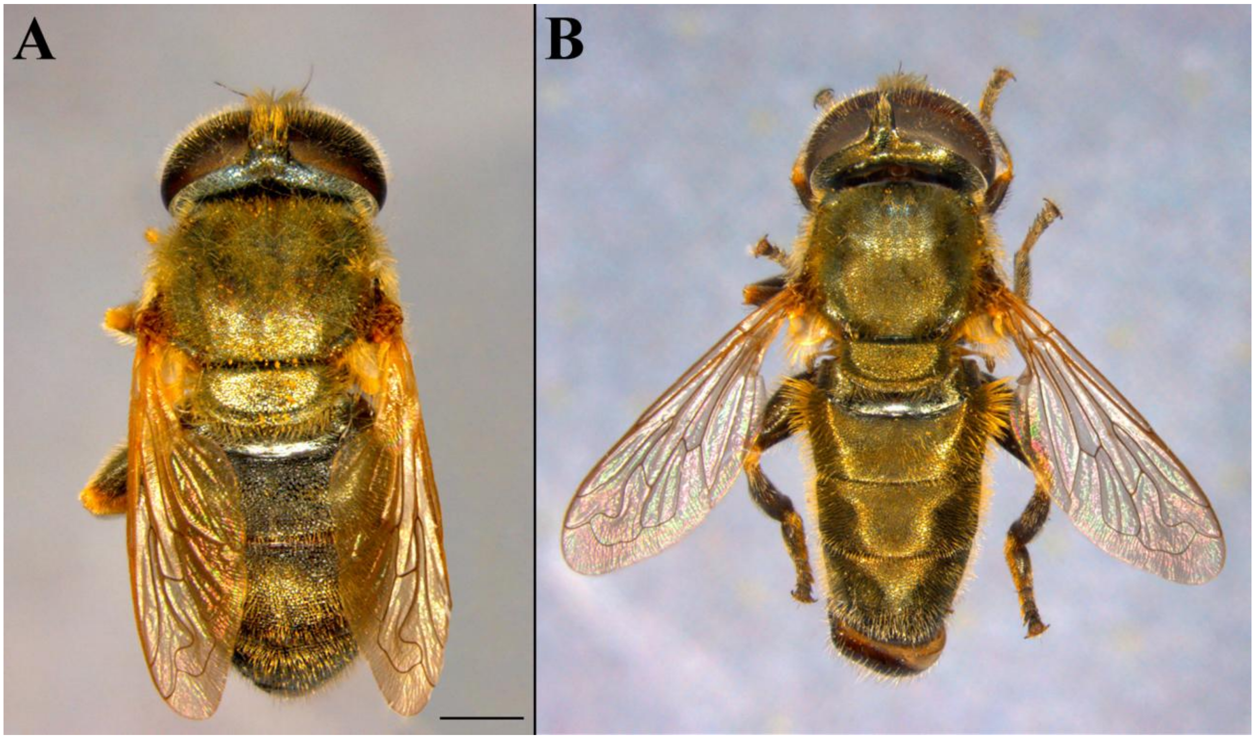

Figure 7. Body of male, dorsal view. **A** *Merodon spinitarsis* **B** *M. nanus* (Sack, 1931). Scale bar: 1 mm.

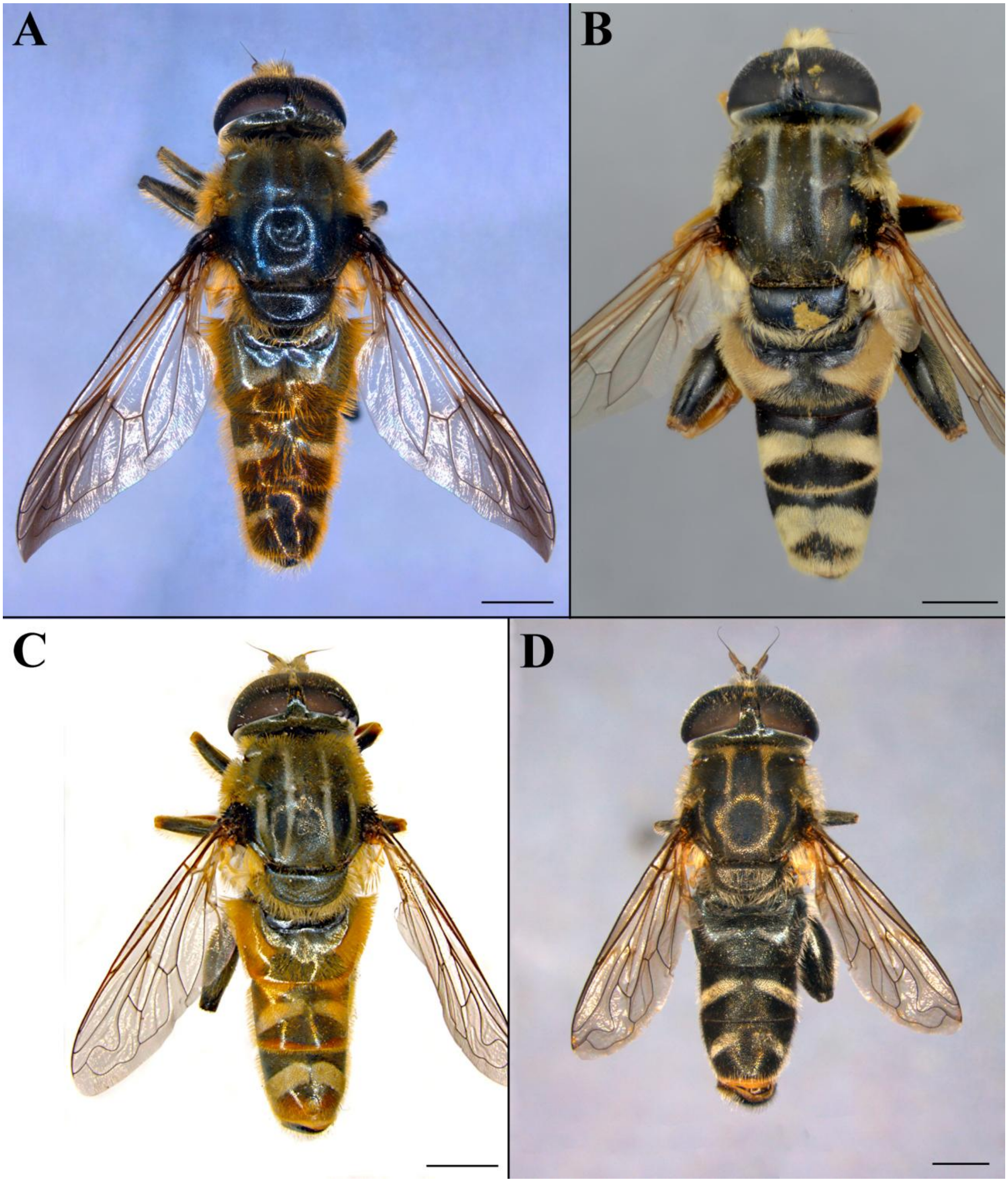

Figure 8. Body of male, dorsal view. **A** *Merodon aberrans* **B** *M. aurifer* **C** *M. avidus* (Rossi, 1790) **D** *M. italicus*. Scale bar: 2 mm (**A–C**); 1.5 mm (**D**).

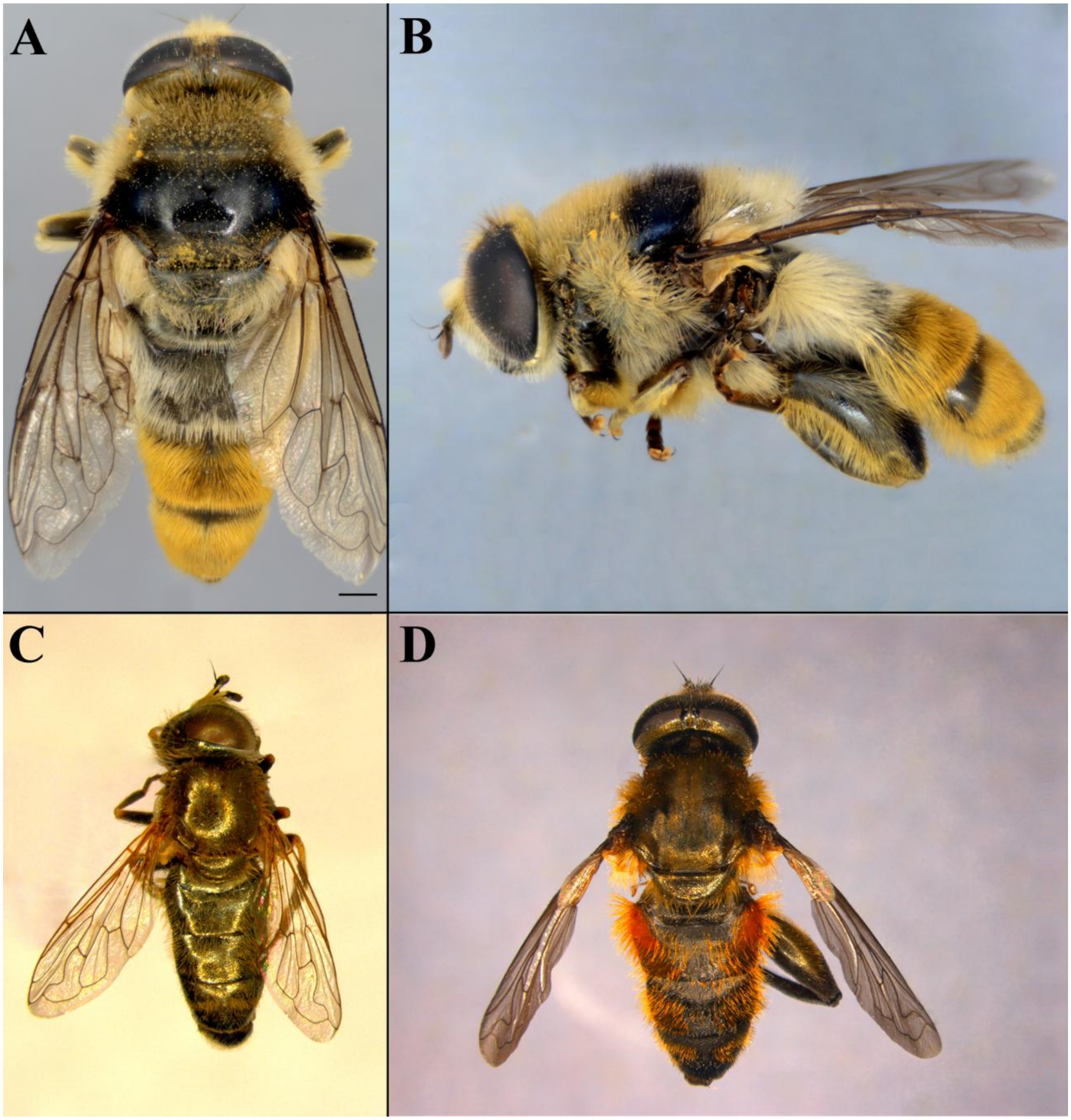

Figure 9. Body of male. **A–B** *Merodon clavipes* (Fabricius, 1781) **C** *M. fulcratus* **D** *M. serrulatus*. **A, C, D** dorsal view **B** lateral view. Scale bar: 1 mm.

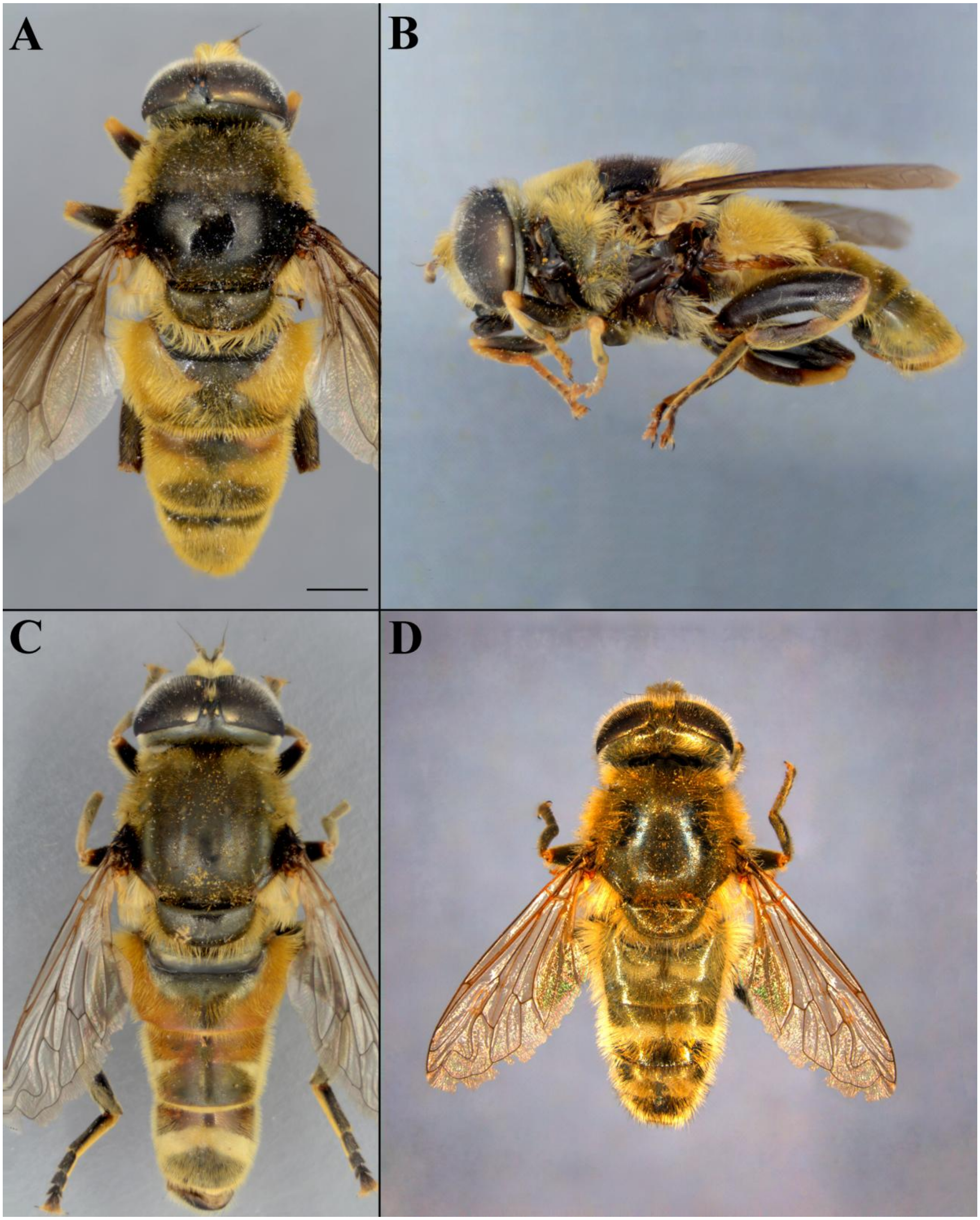

Figure 10. Body of male. **A–B** *Merodon pruni* **C** *M. nigritarsis* Rondani, 1845 **D** *M. auronitens*. **A, C, D** dorsal view **B** lateral view. Scale bar: 2 mm (**A–C**); 1.5 mm (**D**).

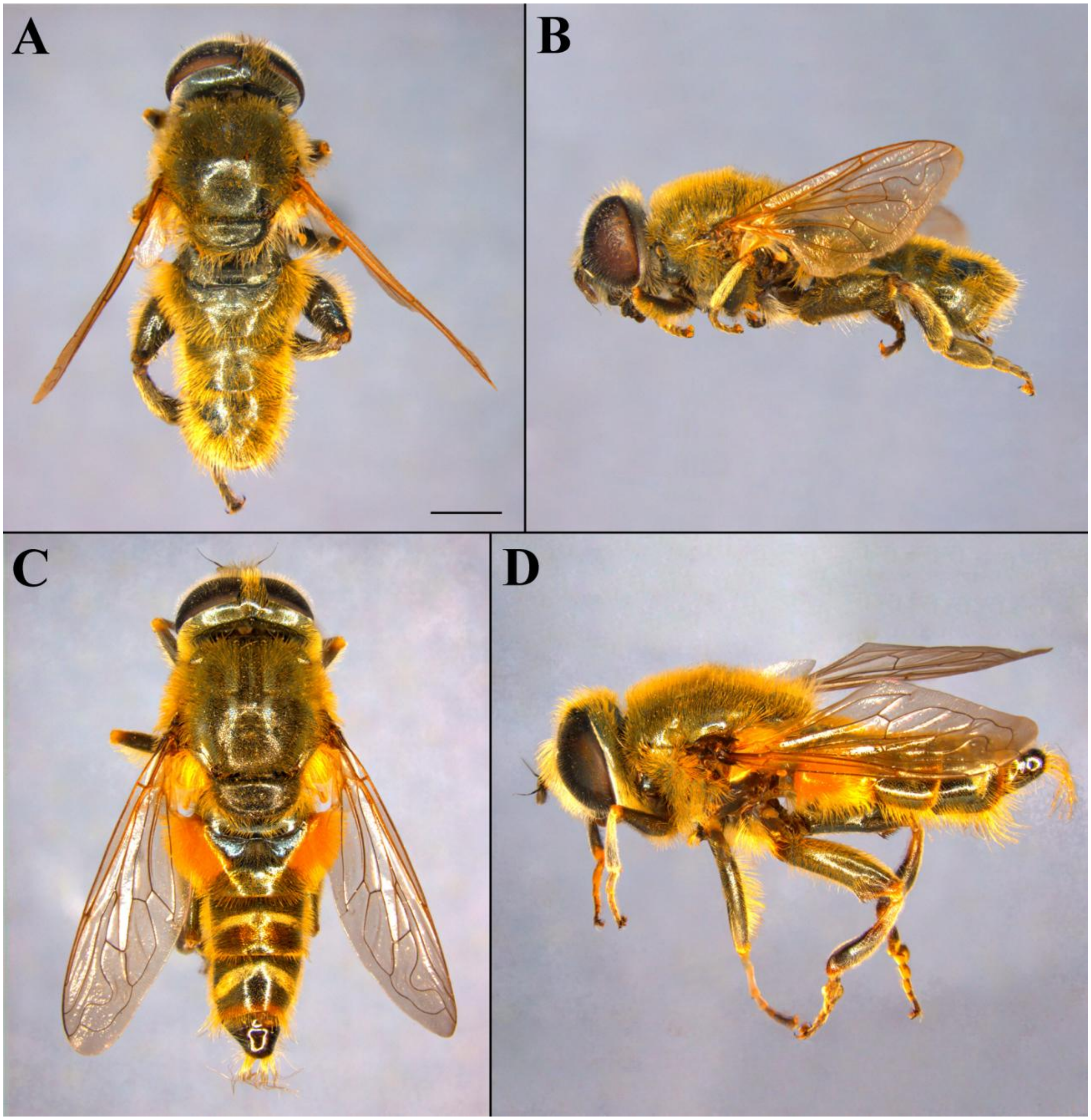

Figure 11. Body of male. **A–B** *Merodon tarsatus* **C–D** *M. caudatus*. **A, C** dorsal view **B, D** lateral view. Scale bar: 1.5 mm.

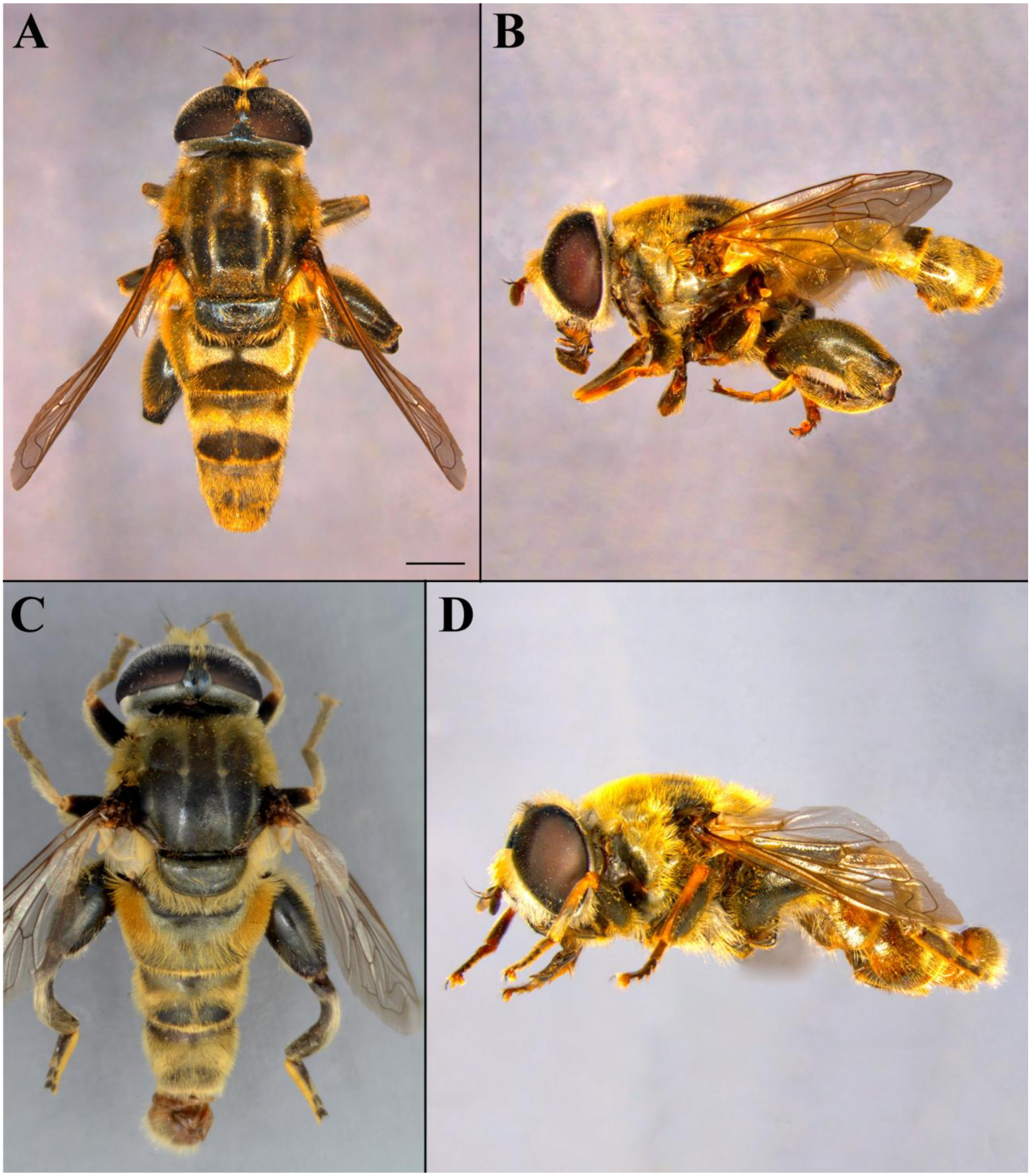

Figure 12. Body of male. **A–B** *Merodon clunipes* **C–D** *M. crassifemoris*. **A, C** dorsal view **B, D** lateral view. Scale bar: 2 mm.

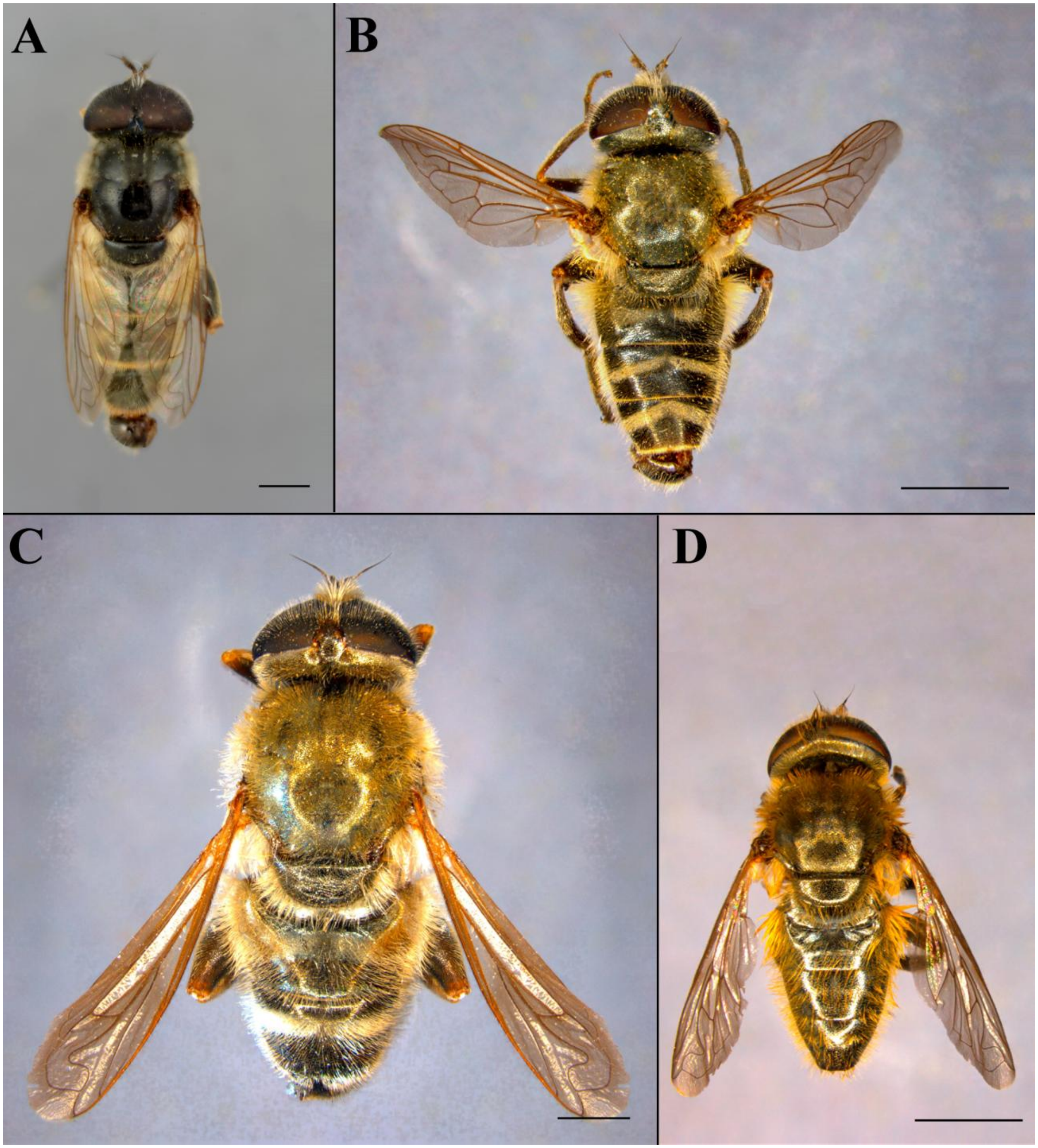

Figure 13. Body of male, dorsal view. **A** *Merodon eumerusi* **B** M. *hirtus* **C** *M. murinus* **D** *M. ottomanus*. Scale bar: 1.5 mm (**A, C**); 2 mm (**B, D**).

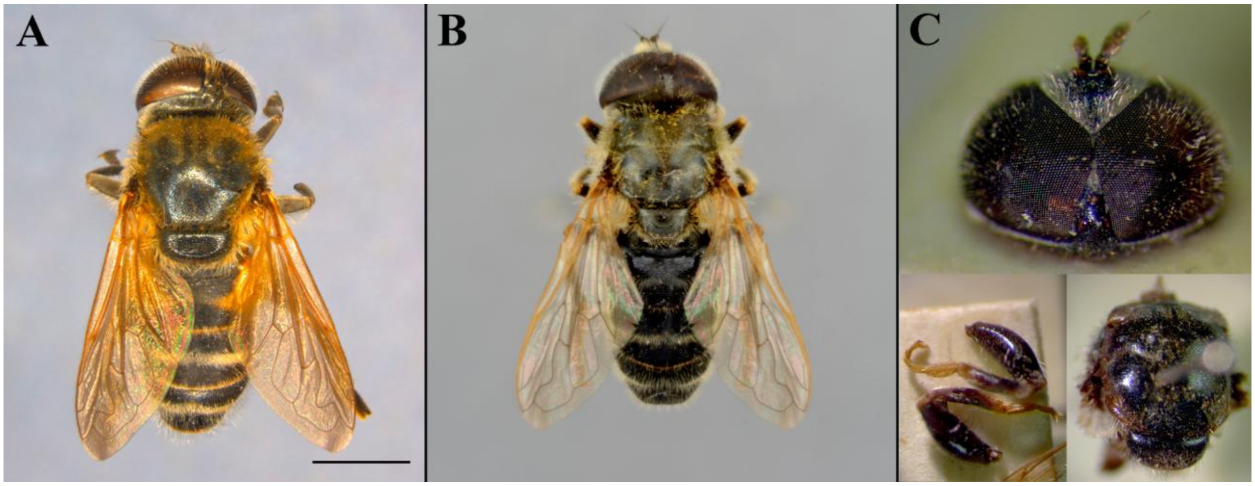

Figure 14. Body of male, dorsal view. **A** *Merodon melanocerus* **B** *M. desuturinus* **C** *M. cuthbertsoni*. Scale bar: 2 mm (**A–B**).

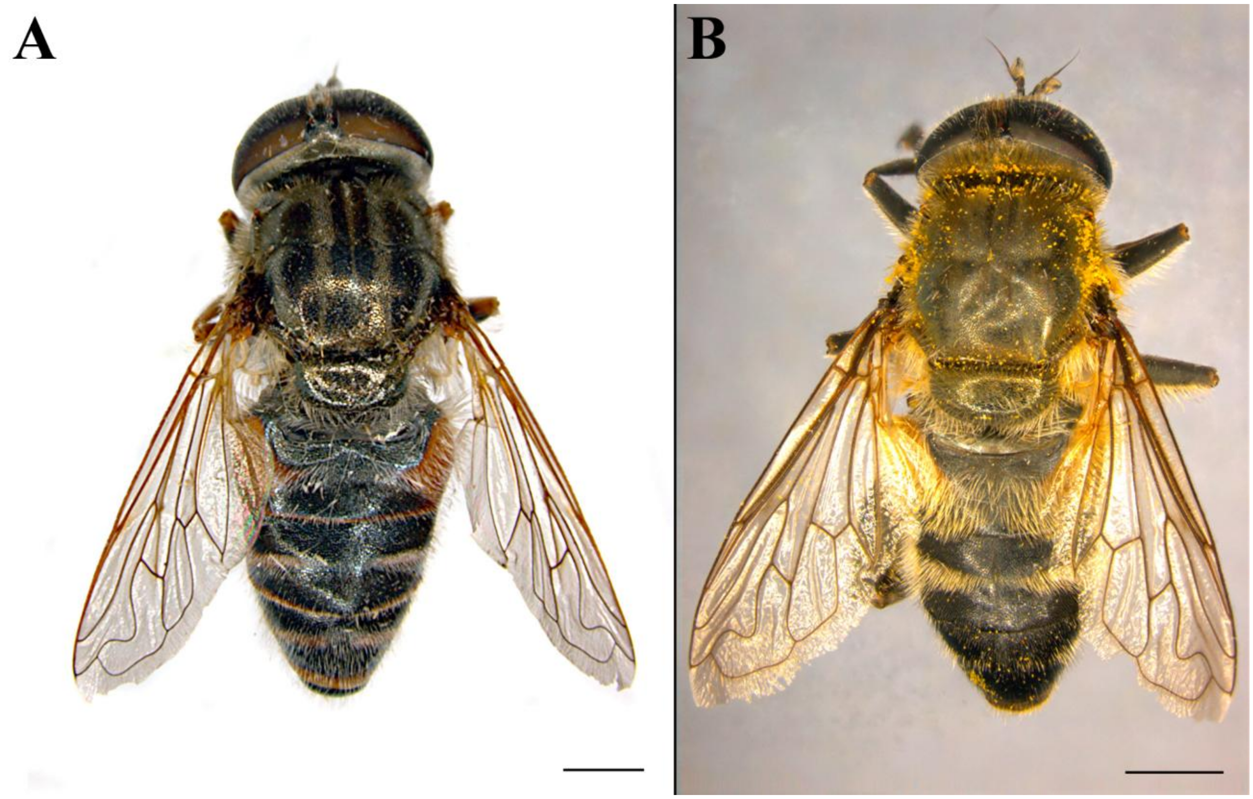

Figure 15. Body of male, dorsal view. **A** *Merodon natans* (Fabricius, 1794) **B** *M. segetum*. Scale bar: 1.5 mm (**A**); 2 mm (**B**).
