# Supplementary material for: Taxonomic complexity in the genus Merodon Meigen, 1803 (Diptera, Syrphidae)
Source: Zookeys. 2021 Apr 14;1031:85–124. doi: 10.3897/zookeys.1031.62125 (PMC8060246; doi:10.3897/zookeys.1031.62125)
Supplement: Supplementary material 4 — List of Merodon species grouped by lineage and species group [file zookeys-1031-085-s004.pdf]

Supplementary file S4. List of *Merodon* species grouped by lineage and species group (for each lineage, species group or subgroup, published revisionary works are cited in brackets).

Genus *Merodon* Meigen, 1803

albifrons lineage

albifrons group

1. *Merodon albifrons* Meigen, 1822
2. *Merodon ferrugineus* (Fabricius, 1805)
3. *Merodon flaviventris* (Sack, 1932)
4. *Merodon hurkmansi* Marcos-García, Vujić & Mengual, 2007
5. *Merodon obscuritarsis* Strobl *in* Czerny & Strobl, 1909
6. *Merodon parietum* Wiedemann *in* Meigen, 1822
7. *Merodon stukei* Hauser & Hurkmans, 1997
8. *Merodon trochantericus* Costa, 1884

constans group (Vujić et al. 2020a)

1. *Merodon analis* Meigen, 1822
2. *Merodon caucasicus* Portschinsky, 1877
3. *Merodon chrysotrichos* Vujić, Radenković & Likov *in* Vujić et al. 2020
4. *Merodon chrysurus* Hurkmans & Vujić *in* Vujić et al. 2020
5. *Merodon confinium* Šašić Zorić & Vujić *in* Vujić et al. 2020
6. *Merodon constans* (Rossi, 1794)
7. *Merodon dzhalitae* Paramonov, 1926
8. *Merodon gudaurensis* Portschinsky, 1877
9. *Merodon mishustini* Popov *in* Vujić et al. 2020
10. *Merodon nitens* Hurkmans & Vujić *in* Vujić et al. 2020
11. *Merodon rubidiventris* Costa, 1884
12. *Merodon solitarius* Vujić, Šašić Zorić & Likov *in* Vujić et al. 2020
13. *Merodon spineus* Vujić, Šašić Zorić & Likov *in* Vujić et al. 2020
14. *Merodon spinosus* Vujić, Radenković & Likov *in* Vujić et al. 2020
15. *Merodon triangulum* Vujić, Radenković & Hurkmans *in* Vujić et al. 2020

***equestris* group** (Marcos-García et al. 2011)

1. *Merodon confusus* Marcos-García, Vujić, Ricarte & Ståhls, 2011
2. *Merodon equestris* (Fabricius, 1794)
3. *Merodon flavus* Sack, 1913

***geniculatus* group** (Vujić et al. 2018a)

1. *Merodon albifasciatus* Macquart, 1842
2. *Merodon antonioi* Marcos-García, Vujić & Mengual, 2007
3. *Merodon chalybeatus* Sack, 1913
4. *Merodon crypticus* Marcos-García, Vujić & Mengual, 2007
5. *Merodon eques* (Fabricius, 1805)
6. *Merodon escorialensis* Strobl *in* Czerny & Strobl, 1909
7. *Merodon geniculatus* Strobl *in* Czerny & Strobl, 1909
8. *Merodon longispinus* Marcos-García, Vujić & Mengual, 2007
9. *Merodon luteofasciatus* Vujić, Radenković & Ståhls *in* Vujić et al, 2018
10. *Merodon neofasciatus* Ståhls & Vujić *in* Vujić et al. 2018
11. *Merodon teruelensis* (Van der Goot, 1996)

(undescribed taxa)

12. *Merodon* aff. *albifasciatus*
13. *Merodon* aff. *geniculatus* 1
14. *Merodon* aff. *geniculatus* 2
15. *Merodon* aff. *geniculatus* 3

***ruficornis* group** (Vujić et al. 2012)

1. *Merodon abruzzensis* (Van der Goot, 1964)
2. *Merodon alexandri* Popov, 2009
3. *Merodon armipes* Rondani, 1843
4. *Merodon auripes* Sack, 1913
5. *Merodon gallicus* Vujić & Radenković *in* Vujić et al. 2012
6. *Merodon hoplitis* Hurkmans *in* Vujić et al. 2012

7. *Merodon ilgazense* Vujić, Marcos-García, Sarıbıyık & Ricarte, 2011

8. *Merodon lamellatus* Vujić & Radenković *in* Vujić et al. 2012

9. *Merodon loewi* Van der Goot, 1964

10. *Merodon nigripodus* Vujić & Hayat *in* Vujić et al. 2012

11. *Merodon ovaloides* Vujić & Radenković *in* Vujić et al. 2012

12. *Merodon papillus* Vujić, Radenković & Pérez-Bañon *in* Vujić et al. 2007

13. *Merodon planiceps* Loew, 1862

14. *Merodon ponticus* Vujić & Radenković *in* Vujić et al. 2012

15. *Merodon portschinskyi* (Stackelberg, 1924)

16. *Merodon ruficornis* Meigen, 1822

17. *Merodon trebevicensis* Strobl, 1900

18. *Merodon turcicus* Vujić & Hayat *in* Vujić et al. 2012

***rufus* group** (Radenković et al. 2020)

1. *Merodon kozufensis* Radenković & Vujić *in* Radenković et al. 2020

2. *Merodon olympius* Vujić & Radenković *in* Radenković et al. 2020

3. *Merodon orjensis* Radenković & Vujić *in* Radenković et al. 2020

4. *Merodon rufus* Meigen, 1838

Unplaced species

1. *Merodon luteihumerus* Marcos-García, Vujić & Mengual, 2007

2. *Merodon mixtum* Vujić, Radenković & Likov *in* Vujić et al. 2019

***aureus* lineage**

***aureus* group** (Šašić et al. 2016, 2018, 2019; Veselić et al. 2017; Radenković et al. 2018b; Vujić et al. 2020c)

***aureus* subgroup**

1. *Merodon albidus* Šašić, Ačanski & Vujić *in* Šašić Zorić et al. 2020

2. *Merodon aureus* Fabricius, 1805

3. *Merodon calidus* Šašić, Ačanski & Vujić *in* Šašić Zorić et al. 2020

4. *Merodon ortus* Šašić, Ačanski & Vujić *in* Šašić Zorić et al. 2020

5. *Merodon pumilus* Macquart, 1849

6. *Merodon unicolor* Strobl, 1909

***bessarabicus* subgroup**

7. *Merodon adriaticus* Veselić, Vujić & Radenković, 2017

8. *Merodon andriotes* Vujić, Radenković & Šašić *in* Radenković et al. 2017

9. *Merodon ambiguus* Brădescu, 1986

10. *Merodon bessarabicus* Paramonov, 1924

11. *Merodon euri* Vujić & Radenković *in* Radenković et al. 2017

12. *Merodon erymanthius* Vujić, Ačanski & Šašić *in* Radenković et al. 2017

13. *Merodon flavicornis* Macquart, 1842

14. *Merodon hayati* Hurkmans, 1997

15. *Merodon kiritshenkoi* (Stackelberg, 1960)

16. *Merodon legionensis* Marcos-García, Vujić & Mengual, 2007

17. *Merodon luteomaculatus* Vujić, Ačanski & Šašić *in* Radenković et al. 2017

18. *Merodon naxius* Vujić & Šašić *in* Radenković et al. 2017

19. *Merodon nisi* Veselić, Vujić & Radenković, 2017

20. *Merodon peloponnesius* Vujić, Radenković, Ačanski & Šašić *in* Radenković et al. 2017

21. *Merodon quercetorum* Marcos-García, Vujić & Mengual, 2007

22. *Merodon rufipes* Sack, 1913

23. *Merodon sapphous* Vujić, Pérez-Bañon & Radenković *in* Vujić et al. 2007

(undescribed taxa)

24. *Merodon* aff. *ambiguus* 1

25. *Merodon* aff. *ambiguus* 2

26. *Merodon* aff. *ambiguus* 3

27. *Merodon* aff. *bessarabicus*

28. *Merodon* aff. *sapphous* 1

29. *Merodon* aff. *sapphous* 2

30. *Merodon* aff. *sapphous* 3

31. *Merodon* aff. *sapphous* 4

***cinereus* subgroup**

32. *Merodon aerarius* Rondani, 1857

33. *Merodon atratus* Oldenberg, 1919

34. *Merodon balkanicus* Šašić, Ačanski & Vujić *in* Šašić et al. 2016

35. *Merodon cinereus* (Fabricius, 1794)

36. *Merodon virgatus* Vujić & Radenković *in* Šašić et al. 2016

***chalybeus* subgroup**

37. *Merodon chalybeus* Wiedemann *in* Meigen, 1822

38. *Merodon minutus* Strobl, 1893

39. *Merodon robustus* Veselić, Vujić & Radenković, 2017

***caerulescens* subgroup**

40. *Merodon atricapillatus* Šašić, Ačanski & Vujić *in* Šašić Zorić et al. 2018

41. *Merodon caerulescens* Loew, 1869

***dobrogensis* subgroup**

42. *Merodon dobrogensis* Bradescu,1982

43. *Merodon puniceus* Vujić, Radenković & Pérez-Bañón *in* Radenković et al. 2011

44. *Merodon rojoi* Radenković & Vujić *in* Šašić Zorić et al. 2019

Unplaced species

45. *Merodon unguicornis* Strobl *in* Czerny & Strobl, 1909

***bombiformis* group**

1. *Merodon bombifornis* Hull, 1944
2. *Merodon multifasciatus* Curran, 1939
3. *Merodon nasicus* Bezzi, 1915

(undescribed species)

4. *Merodon* aff. *multifasciatus* 1
5. *Merodon* aff. *multifasciatus* 2
6. *Merodon* aff. *nasicus*

***funestus* group**

1. *Merodon funestus* (Fabricius, 1794)

(undescribed species)

2. *Merodon* aff. *funestus*

***nanus* group** (Vujić et al. 2015; Kočiš Tubić et al. 2018)

1. *Merodon kopensis* Vujić & Hayat *in* Vujić et al. 2015
2. *Merodon nanus* (Sack, 1931)
3. *Merodon neonanus* Vujić & Taylor *in* Vujić et al. 2015
4. *Merodon rasicus* Vujić & Radenković *in* Vujić et al. 2015
5. *Merodon telmateia* Hurkmans, 1987
6. *Merodon vladimiri* Vujić & Kočiš Tubić *in* Kočiš Tubić et al. 2018

***spinitarsis* group**

1. *Merodon spinitarsis* Paramonov, 1929

(undescribed species)

2. *Merodon* aff. *spinitarsis*

avidus-nigritarsis lineage

***aberrans* group**

1. *Merodon aberrans* Egger, 1860
2. *Merodon brevis* Paramonov, 1925
3. *Merodon flavitibius* Paramonov, 1926
4. *Merodon hamifer* Sack, 1913

(undescribed species)

5. *Merodon* aff. *aberrans* 1
6. *Merodon* aff. *aberrans* 2
7. *Merodon* aff. *aberrans* 3
8. *Merodon* aff. *aberrans* 4

***aurifer* group**

1. *Merodon aurifer* Loew, 1862

(undescribed species)

2. *Merodon* aff. *aurifer*

***avidus* group** (Popović et al. 2015; Ačanski et al. 2016b; Likov et al. 2020)

1. *Merodon avidus* (Rossi, 1790)
2. *Merodon femoratus* Sack, 1913
3. *Merodon ibericus* Vujić *in* Popović et al. 2015
4. *Merodon megavidus* Vujić & Radenković *in* Ačanski et al. 2016
5. *Merodon moenium* Wiedeman *in* Meigen, 1822
6. *Merodon rutitarsis* Likov, Vujić & Radenković *in* Likov et al. 2020

***clavipes* group**

1. *Merodon clavipes* (Fabricius, 1781)
2. *Merodon quadrinotatus* (Sack, 1931)
3. *Merodon vandergooti* Hurkmans, 1993
4. *Merodon velox* Loew, 1869

***fulcratus* group**

1. *Merodon dichopticus* Stackelberg, 1968
2. *Merodon fulcratus* (Becker, 1913)

***italicus* group**

1. *Merodon italicus* Rondani, 1845
2. *Merodon erivanicus* Paramonov, 1925

***nigritarsis* group** (Vujić et al. 2013; Likov et al. 2020)

***alagoezicus* subgroup**

1. *Merodon alagoezicus* Paramonov, 1925
2. *Merodon hakkariensis* Vujić & Radenković *in* Vujić et al. 2013
3. *Merodon lucasi* Hurkmans, 1993
4. *Merodon nitidifrons* Hurkmans, 1993
5. *Merodon satgadensis* Hurkmans, 1993
6. *Merodon schachti* Hurkmans, 1993

***nigritarsis* subgroup**

7. *Merodon angustus* Vujić & Radenković *in* Vujić et al. 2013
8. *Merodon cohurnus* Vujić, Likov & Radenković *in* Likov et al. 2020
9. *Merodon femoratoides* Paramonov, 1925
10. *Merodon latifemoris* Radenković & Vujić *in* Radenković et al. 2011
11. *Merodon longisetus* Vujić, Radenković & Likov *in* Likov et al. 2020
12. *Merodon nigritarsis* Rondani, 1845
13. *Merodon obstipus* Vujić, Radenković & Likov *in* Likov et al. 2020
14. *Merodon quadraticus* Vujić & Radenković *in* Vujić et al. 2013
15. *Merodon taniniensis* Hurkmans, 1993
16. *Merodon testaceus* Sack,1913
17. *Merodon toscanus* Hurkmans, 1993

***pruni* group**

1. *Merodon cupreus* Hurkmans, 1993
2. *Merodon pallidus* Macquart, 1842
3. *Merodon pruni* (Rossi, 1790)

(undescribed species)

4. *Merodon* aff. *pruni*

***serrulatus* group** (Vujić et al. 2020b)

1. *Merodon bequaerti* Hurkmans, 1993
2. *Merodon defectus* Vujić, Likov & Radenković *in* Vujić et al. 2020
3. *Merodon disjunctus* Vujić, Likov & Radenković *in* Vujić et al. 2020
4. *Merodon hirsutus* Sack, 1913
5. *Merodon kawamurai* Matsumura, 1916
6. *Merodon medium* Vujić, Likov & Radenković *in* Vujić et al. 2020
7. *Merodon nigrocapillatus* Vujić, Likov & Radenković *in* Vujić et al. 2020
8. *Merodon nigropunctum* Vujić, Likov & Radenković *in* Vujić et al. 2020
9. *Merodon opacus* Vujić, Likov & Radenković *in* Vujić et al. 2020
10. *Merodon sacki* (Paramonov, 1936)
11. *Merodon serrulatus* Wiedemann *in* Meigen, 1822

12. *Merodon sophron* Hurkmans, 1993

13. *Merodon trianguloculus* Vujić, Likov & Radenković *in* Vujić et al. 2020

***tarsatus* group**

1. *Merodon hypochrysos* Hurkmans, 1993
2. *Merodon marginicornis* Hurkmans, 1993
3. *Merodon oidipous* Hurkmans, 1993
4. *Merodon rufitarsis* Sack, 1913
5. *Merodon smirnovi* Paramonov, 1927
6. *Merodon tarsatus* Sack, 1913
7. *Merodon turkmestanicus* Paramonov, 1926

(undescribed species)

8. *Merodon* aff. *tarsatus* 1
9. *Merodon* aff. *tarsatus* 2
10. *Merodon* aff. *tarsatus* 3
11. *Merodon* aff. *tarsatus* 4
12. *Merodon* aff. *tarsatus* 5
13. *Merodon* aff. *tarsatus* 6

Unplaced species

1. *Merodon auronitens* Hurkmans, 1993
2. *Merodon caudatus* Sack, 1913
3. *Merodon clunipes* Sack, 1913
4. *Merodon crassifemoris* Paramonov, 1925
5. *Merodon eumerusi* Vujić, Radenković & Likov *in* Vujić et al. 2019
6. *Merodon hirtus* (Sack, 1932)
7. *Merodon murinus* Sack, 1913
8. *Merodon ottomanus* Hurkmans, 1993

desuturinus lineage

***melanocerus* group (Afrotropical Region)** (Radenković et al. 2018a; Djan et al. 2020)

***melanocerus* subgroup**

1. *Merodon capensis* Hurkmans 2018 *in* Radenković et al. 2018
2. *Merodon commutabilis* Radenković & Vujić *in* Radenković et al. 2018
3. *Merodon drakonis* Vujić & Radenković *in* Radenković et al. 2018
4. *Merodon flavocerus* Hurkmans 2018 *in* Radenković et al. 2018
5. *Merodon melanocerus* Bezzi, 1915

***planifacies* subgroup**

6. *Merodon capi* Vujić & Radenković *in* Djan et al. 2020
7. *Merodon planifacies* Bezzi, 1915
8. *Merodon roni* Radenković & Vujić *in* Djan et al. 2020
9. *Merodon stevensoni* Curran, 1939

(undescribed species)

10. *Merodon* aff. *capi* 1
11. *Merodon* aff. *capi* 2
12. *Merodon* aff. *capi* 3
13. *Merodon* aff. *planifacies* 1
14. *Merodon* aff. *planifacies* 2
15. *Merodon* aff. *planifacies* 3
16. *Merodon* aff. *planifacies* 4
17. *Merodon* aff. *planifacies* 5
18. *Merodon* aff. *planifacies* 6
19. *Merodon* aff. *planifacies* 7

Unplaced species

4. *Merodon cuthbertsoni* Curran, 1939

***murorum* group (Palaearctic Region)** (Vujić et al. 2018b)

1. *Merodon cabanerensis* Marcos-García, Vujić & Mengual, 2007
2. *Merodon desuturinus* Vujić, Šimić & Radenković, 1995

3. *Merodon murorum* (Fabricius, 1794)

4. *Merodon neolydicus* Vujić *in* Vujić et al. 2018

***natans* lineage**

***natans* group**

1. *Merodon calcaratus* (Fabricius, 1794)

2. *Merodon natans* (Fabricius, 1794)

3. *Merodon pulveris* Vujić & Radenković *in* Radenković et al. 2011

(undescribed species)

4. *Merodon* aff. *natans*

Unplaced species

1. *Merodon segetum* (Fabricius 1794)
