# Supplementary material for: Taxonomic complexity in the genus Merodon Meigen, 1803 (Diptera, Syrphidae)
Source: Zookeys. 2021 Apr 14;1031:85–124. doi: 10.3897/zookeys.1031.62125 (PMC8060246; doi:10.3897/zookeys.1031.62125)
Supplement: Supplementary material 5 — Table S1. Classification of the genus Merodon in lineages and species groups, including an actual number of species within each [file zookeys-1031-085-s005.pdf]

Supplementary file S5. Table 1 Classification of the genus *Merodon* in lineages and species groups, including an actual number of species within each (Ds Number of described species; Un Number of undescribed species).

lineage	group	subgroup	unplaced species	Ds	Un	Total
albifrons	*albifrons*			8		8
	constans			15		15
	equestris			3		3
	geniculatus			11	4	15
	ruficornis			18		18
	rufus			4		4
			M. luteihumerus	1		1
			M. mixtum	1		1
				**61**	**4**	**65**
aureus	*aureus*	*aureus*		6		6
		bessarabicus		17	8	25
		cinereus		5		5
		chalybeus		3		3
		caerulescens		2		2

		dobrogensis		3		3
			M. unguicornis	1		1
	funestus			1	1	2
	nanus			6		6
	spinitarsis			1	1	2
	bombiformis			3	3	6
				**48**	**13**	**61**
avidus-nigritarsis	*aberrans*			4	4	8
	aurifer			1	1	2
	avidus			6		6
	clavipes			4		4
	fulcratus			2		2
	italicus			2		2
	nigritarsis	*alagoezicus*		6		6
		nigritarsis		11		11
	pruni			3	1	4
	serrulatus			13		13
	tarsatus			7	6	13

			M. auronitens	1		1
			M. caudatus	1		1
			M. clunipes	1		1
			M. crassifemoris	1		1
			M. eumerusi	1		1
			M. hirtus	1		1
			M. murinus	1		1
			M. ottomanus	1		1
				**67**	**12**	**79**
desuturinus	*melanocerus* (Afrotropical)	*melanocerus*		5		5
		planifacies		4	10	14
			cuthbetsoni	1		1
	murorum (Palaearctic)			4		4
				**14**	**10**	**24**
natans	*natans*			3	1	4
			M. segetum	1		1
				**4**	**1**	**5**
Total				**194**	**40**	**234**
